# Developing, delivering and evaluating primary mental health care: the co-production of a new complex intervention

**DOI:** 10.1186/s12913-016-1726-6

**Published:** 2016-09-06

**Authors:** Joanne Reeve, Lucy Cooper, Sean Harrington, Peter Rosbottom, Jane Watkins

**Affiliations:** 1 Warwick Medical School, Warwick University, Coventry, CV4 7AL UK; 2 Division of Health Sciences Research, Liverpool University, Liverpool, L69 3GL UK; 3 AiW Health, 38-44 Woodside Business Park, Birkenhead, Wirral CH41 1EL UK; 4 http://www2.warwick.ac.uk/fac/med/staff/jreeve

**Keywords:** Normalisation Process Theory (NPT), Complex intervention, Practice-based evidence, Flipped care, Translational research, Mental health

## Abstract

**Background:**

Health services face the challenges created by complex problems, and so need complex intervention solutions. However they also experience ongoing difficulties in translating findings from research in this area in to quality improvement changes on the ground. BounceBack was a service development innovation project which sought to examine this issue through the implementation and evaluation in a primary care setting of a novel complex intervention.

**Methods:**

The project was a collaboration between a local mental health charity, an academic unit, and GP practices. The aim was to translate the charity’s model of care into practice-based evidence describing delivery and impact. Normalisation Process Theory (NPT) was used to support the implementation of the new model of primary mental health care into six GP practices. An integrated process evaluation evaluated the process and impact of care.

**Results:**

Implementation quickly stalled as we identified problems with the described model of care when applied in a changing and variable primary care context. The team therefore switched to using the NPT framework to support the systematic identification and modification of the components of the complex intervention: including the core components that made it distinct (the consultation approach) and the variable components (organisational issues) that made it work in practice. The extra work significantly reduced the time available for outcome evaluation. However findings demonstrated moderately successful implementation of the model and a suggestion of hypothesised changes in outcomes.

**Conclusions:**

The BounceBack project demonstrates the development of a complex intervention from practice. It highlights the use of Normalisation Process Theory to support development, and not just implementation, of a complex intervention; and describes the use of the research process in the generation of practice-based evidence. Implications for future translational complex intervention research supporting practice change through scholarship are discussed.

**Electronic supplementary material:**

The online version of this article (doi:10.1186/s12913-016-1726-6) contains supplementary material, which is available to authorized users.

## Complex problems: developing the complex intervention solutions

The problems facing our health systems have changed rapidly in the last hundred years. Health problems are increasingly characterised by chronicity [1], and complexity (the co-existence of multiple, interacting components [2]). A growing proportion of our population live with the variable and varying impacts not only of multimorbidity (multiple long term conditions) [3], but also treatment burden [4–6], problematic polypharmacy [7], distress [8], and social inequalities [9]. Complex problems need complex solutions.

In a world of evidence-based practice and policy, this has created a new challenge for the scientific community. An intervention is defined as complex (rather than complicated [10]) because it consists of a number of *interacting* components [11]. Uncontrolled and uncontrollable variation is therefore inevitable; the ‘active ingredient(s)’ may vary in different contexts and for different people. Both elements create problems for traditional clinical evaluation (especially trial) designs. The Medical Research Council has responded with a series of guidance documents to support the process of translating an idea for a complex intervention into the evidence-based practice needed to support implementation [11, 12].

The guidance recognises the stages of complex interventions research from development (including piloting and feasibility) through to evaluation (including process evaluation [13]) and implementation. Two components to the development process are advocated: theory driven development of the intervention, shaped by a formative evaluation to assess participant engagement with, and contextual impact on, the (new) ideas [14].

The current guidance is based on what has been described as a pipeline model of evidence-based practice and knowledge translation [15] (for an illustration, see Green cited in [15]). The pipeline model derives from our current understanding of the best way to produce legitimate knowledge for practice. In the pipeline model, knowledge is (best) produced within the ‘objective’ space of scientific study and then transferred in to the applied context in which it is used.

The pipeline model [15] of developing evidence-based practice has stood us in good stead, particularly in the management of chronic disease. A growing wealth of clinical trial evidence tells us how we can better control and manage risk related to a range of chronic diseases including diabetes, cancer and cardiovascular problems [16–18]. Passed down the pipeline, this knowledge supports the generation of new interventions to identify and address disease risk. This work contributed, for example, to a 40 % drop in cardiovascular mortality in the last 10 years [19].

**Fig. 1:**
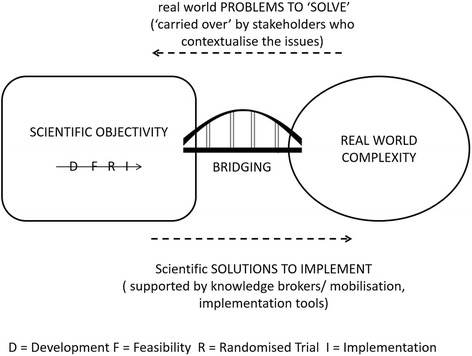
Assumptions behind the pipeline model

But alone, the pipeline model may not be adequate in the new complex world in which practitioners find themselves. The inefficiency of the model has been recognised for some time. In 2000, it was reported that it takes 17 years to translate 14 % of clinical research down the pipeline and into front-line practice [20]. In response we saw a growth in translational research – ways to ‘plug the leaks’ in the pipeline and so improve the rate and process of transfer. A range of initiatives have emerged including greater stakeholder involvement in the design and undertaking of research (what enters the pipeline); the emergence of knowledge brokers and knowledge mobilisation techniques and roles (to support movement along the pipeline); and most recently, a new implementation science [21]. Implementation science recognises that excellent clinical trials can provide evidence of what clinicians could do, but that does not necessarily change their practice [19]. The development of implementation solutions has seen a growth of decision aids, consultation templates, and educational models. All work by extending the pipeline in order to bridge the gap between science and practice (Fig. 1).

The pipeline model assumes that the primary challenge we face in using research to drive quality improvement is one of improving efficiency in implementing scientific knowledge in practice. But scientific trials, even of complex interventions, produce knowledge that is only one small part of the knowledge needed in the complex decision making process faced on a daily basis by patients and clinicians. Medical science delivers an incomplete evidence base, and we need to recognise this as part of the problem. A key gap in quality improvement is a lack of knowledge [22]. Clinicians and patients therefore face a daily task of creating ‘practical’ knowledge to fill the gaps in our scientific knowledge [22]. Green proposed that we should better understand this experiential, often tacit, knowledge, and use it to develop so-called practice-based evidence [15]. Practice-based contextual knowledge offers a potentially valuable, and as yet underexplored, resource in the development of innovative solutions to the new problems facing modern health systems [23]. Introducing a two-way flow of knowledge between practice and evidence requires a rethink of the pipeline model of driving quality improvement through research. It requires a shift in our approach in moving from translating research output into practice, to “optimising health care through [a process of] research and quality improvement” [22].

This paper describes work to develop and evaluate a primary mental health care complex intervention. The work – essentially a formative evaluation [14] - started as a pipeline model, but finished as a ‘co-production’ model [24] with a two-way flow of knowledge into practice. We use the experience to consider the implications for updating our understanding of developing and evaluating complex interventions in order to drive quality improvement.

## Introducing the BounceBack project

BounceBack aimed to challenge and change current thinking about how to assess and manage mental health and wellbeing in a primary care setting. The innovation project was run as a partnership between AIW Health and Liverpool University. AIW Health is a UK mental health charity offering an alternative ‘flipped model’ [25] (Table 1) of mental health care to local residents experiencing mild to moderate distress. Client feedback from 20 years’ practical experience of delivering care suggests that the model is effective but as yet, no formal evidence exists to support a change in health care systems to this way of thinking. The BounceBack project therefore aimed to translate the AIW Health care model into practice-based evidence that, if appropriate, could support wider roll out of the approach.

**Table 1:** Describing a flipped model of mental health care

The AIW Health approach to understanding and addressing mental health need flips the traditional medical model on its head. Current UK medical mental health care starts with a health professional assessing whether an individual meets diagnostic criteria for mental illness. Appropriate medical treatment is initiated, and the individual may also be referred on, if appropriate, for help with practical concerns that might limit healing, for example debt advice. In terms of a biopsychosocial model of care, it is the ‘biopsycho’ element that is dominant, with ‘psychosocial’ components seen as a backup.
Care at AIW Health takes the reverse approach. Care starts with a non-biomedical, whole person assessment of experiences of distress undertaken by an AiW case worker. Practitioner and patient work to identify and address the practical and social issues contributing to distress. Only if mental health issues remain is a biomedical approach employed (through referral on to NHS care). Service users and members of the public have both reported that the psychosocial-dominant AIW Health approach describes a service they would want to use. It provides a service that addresses *their* needs (recognition), works with them to deal with problems (reciprocity), and leaves them better able to manage issues in the future (resilience). (See http://www.primarycarehub.org.uk/projects/bounceback) Anecdotal evidence from the charity therefore suggests that this ‘flipped’ approach [25] could address the highlighted concerns about access and inequalities.

To support the generation of this practice-based evidence, we conceptualised the flipped model as a complex intervention. This allowed us to apply the principles of scientific enquiry – including the use of Normalisation Process Theory, NPT [26] (Table 2) – to support implementation and evaluation. During the process, we experienced a number of problems which required us to amend our understanding of both the intervention and the generation of practice-based evidence. Here, we describe the work of the BounceBack project in order to critically consider the implications for future complex intervention development and the generation of practice-based evidence.

**Table 2:** Describing complex interventions and Normalisation Process Theory

Normalisation Process Theory (NPT) [26] predicts that successful implementation of a complex intervention needs continuous work in four areas which, for the purposes of discussion with our stakeholders, we described as: Sense making, Engagement, Action and Monitoring
SENSE MAKING: people must individually and collectively understand what the new way of working is; how it is different from what went before; and why it matters.
ENGAGEMENT: people must agree to start doing the new model of care, and continue working at it.
ACTION: people need to have the resources to work in the new way.
MONITORING: people need to get feedback that reinforces the new way of working.
The NPT toolkit [26] is designed to support the implementation of complex interventions by helping practitioners examine the nature and extent of the implementation work being undertaken in each of the four domains. Questions explored include [27]:
Sense making: How is a practice understood by participants, and compared with others? Engagement: How do participants come to take part in a practice, and stay motivated?
Actions: How do participants make it work? How are their activities organised and structured?
Monitoring: How do participants evaluate a practice? How does this change over time and what are its effects?

## Background to the BounceBack project

### The problem: the need to improve access to appropriate primary mental health care

Depression is a leading global cause of disability [28], and one which is inequitably distributed within society. Inequalities in care relate, in part, to problems with access to care; where access problems arise from the *nature* and not just the availability of care [9]. Overreliance on a biomedical disease-focused account of mental illness contributes to inequalities through access problems that go beyond availability. Kovandžić [9] described these as candidacy, concordance and recursivity (see Table 3). Authors have called for a need to recognise going beyond a medical approach to understanding mental health problems in order to address inequalities and improve care [9, 29]. The BounceBack project sought to address this challenge.

**Table 3:** Understanding the concept of Access

Improving equitable access to appropriate mental health care needs services which adequately address three elements:
• RECOGNITION: (referred to by Kovandžić [9] as candidacy) whether the individual recognises themselves as eligible/suitable for the service, and the service as suitable for them
▪ RECIPROCITY: (referred to by Kovandžić as concordance) whether the individual is successfully able to work with the service to address their health problems (including whether the service offered matches needs)
▪ RESILIENCE: (referred to by Kovandžić as recursivity) whether the service leaves the individual with (an enhanced) capacity to deal with similar problems in the future

### The ‘flipped care’ approach

AiW Health recognises that practical problems are often the primary factor(s) in many people’s mental health problems. In their flipped care [25] approach, practitioners first work to address practical, social, and related psychological concerns (a socio-psycho approach); only turning to a medical (bio-psycho) approach if distress persists. The AiW Health practice-based model resonates with an academic account developed by Reeve [30] - the Self Integrity Model (SIM). The SIM recognises disabling distress as resulting from an imbalance between the demands on, and resources available to, an individual in maintaining daily living. The goal of care is therefore to identify and address the causes of the imbalance. Patient and practitioner work together to recognise (potential) disruptions to daily living, to mutually identify and address modifiable areas, and in so doing leave individuals better able to adapt to any future changes.

AiW Health’s care model is an empirical one, built from practical experience. SIM is a theoretical one, built from empirical research. Discussions between SH (AIW Health) and JR (Liverpool University) recognised the overlap between the two models, and their potential to support a service redesign which addresses the highlighted issues about access. With both theoretical and practical support for a ‘flipped model’ of care, we successfully bid for Department of Health Innovation Excellence and Strategic Development funding [31] to support the introduction of this new model of care into a primary care setting. Our proposition being that a flipped model might improve both mental health and care through addressing access issues related to recognition, reciprocity and resilience [9]. The essential elements of the BounceBack model at the outset of the project are shown in Table 4.

**Table 4:** BB1 - the original BounceBack Intervention

An integration of the Self Integrity Model [30]* with the AiW Health approach**
Approach
▪ Adopts a person centred understanding of distress, resulting from an imbalance between resources and demands*
▪ Imbalance is explored and understood through open conversation focused on the patients experience*^,^**
▪ Identifying potentially remediable gaps in (practical) support in order to identify action points^**^
Delivery^**^
▪ Delivered by AIW Health case workers embedded into the primary healthcare team
▪ First assessment visit supports formulation of an action plan
▪ Follow up until practical problems limiting daily living and engagement with meaningful occupation addressed
▪ Resilience/forward planning meeting once immediate issues resolved, to consolidate learning (dealing with future problems), action plan for maintenance, and future contact route if needed.
▪ Recorded in the practice records to support integration with the clinical team

* indicates areas of the BounceBack intervention developed from the Self Integrity Model; ** indicates elements taken from the AiW Health Approach

## Aims

Our overall research question asks, can implementation of a flipped model of mental health care improve access to, and outcomes from, primary mental health care? The aims for this project were to *integrate* the BounceBack model in a primary care (general practice) setting (Phase 1); and then to *deliver care* including an integrated evaluation to determine merit and worth [32] (Phase 2).

## Phase 1: integration

AiW Health had previous experience of delivering commissioned mental healthcare services (for example debt advice) in the primary care context. Our original implementation proposal therefore described a 4-month plan to integrate the flipped care model in to the primary care setting. The service delivery arm was to be led and delivered by AIW Health. In parallel, Liverpool University research staff would use the NPT toolkit [26] to transparently evaluate the implementation of the service within the new primary care setting.

## Methods

The planned formative evaluation of implementation of our flipped model used a modification of the NPT toolkit [26] to assess the presence/absence and success of work in each of the four described NPT domains (Table 3), for each of three identified stakeholder groups (patients, practitioners, policy makers).

### Data collection

Data collection was via a number of sources including:

- observation of meetings between AIW Health and (potential) participating practices, between case workers and patients, and of the AIW team (LC/JR - weekly AIW team meetings, monthly practice team meetings, LC undertook 4 case worker-patient observations);
- mini interviews with staff and patients during the observation stage (six site visits by LC, feedback from case workers on discussions);
- review of service database (numbers of patients seen by the service - held by AIW administration staff) (weekly activity sheets submitted by JW/PR; discussed at monthly team meetings);
- minutes of two meetings between the project team and local commissioners.

### Analysis

We used a framework approach [35] to manage data and support analysis. The full data set was reviewed and coded to identify data chunks that illustrated work (successful or otherwise) in each of the four NPT domains and for the three stakeholder groups. Table 5 shows the framework used to support coding of the dataset (undertaken by JR, LC).

**Table 5:** Data template used in the evaluation of Phase 1 Implementation stage

Review date:	April 2013			May 2013			June 2013			July 2013		
	Pt	Prac	Pol	Pt	Prac	Pol	Pt	Prac	Pol	Pt	Prac	Pol
Sense Making^a^												
Engagement^a^												
Action^a^												
Monitoring^a^												

*Pt* patient, *Prac* practitioner (primary care and AIW Health), *Pol* policy makers and commissioners

^a^As described/defined in Table 2

We then used a traffic lights system to record the analysis of the data and so monitor progress over time. Within each of the framework cells, we (JR, LC) used a constant comparison approach [35] to assess the data in each cell. Researchers examined whether the various data sources supported a consistent view of continuous work by each stakeholder group.

Where data pointed consistently to work being done, we coded this ‘green’. Where data highlighted no activity at all, we coded this ‘red’. Where data was ambivalent or contradictory, we coded as amber. At monthly team meetings, we fed back the status of our traffic lights to the full team to assess progress towards successful implementation (i.e. ‘green’ in all four areas of work for all stakeholders).

## Results

Table 6 summarises the progression of our traffic lights over the first year of the project. Table 7 (available as Additional file 1) provides examples of data used to support coding decisions of red/amber/green and so the progress of our implementation.

**Table 6:**
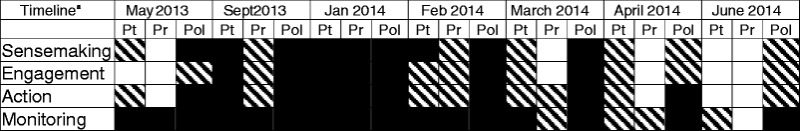
Showing the timeline of progress during the implementation phase

^a^Note the timeline shown is not continuous

*Pt* patient, *Pr* practitioner (both GP and team, and AIW team), *Pol* local policy makers and commissioners

Traffic light Key:  = red  = amber  = green

**Table 7:** Comparing a pipeline (Fig. 1) and incubator (Fig. 2) approach to generating complex intervention evidence

	Pipeline model	Incubator model
Approach	Linear	Circular
Research team	Uses distinct communities and bridges between them	Blurs the boundaries between clinical and academic communities
Outputs	Focused on a study end point, described in terms of statistical certainty of impact	Continuous/evolving output, described in terms of merit and worth of emerging options
Favoured academic model to support the approach	Distinct academic units with methodological expertise	Dispersed academic capacity integrated into the applied context

### Identifying implementation problems

As shown in Table 6, within 3 months of the start of the project, it became apparent that we were hitting implementation problems within all four domains of work and across all stakeholders. Both primary care staff and patients did not have a clear understanding of the new model of practice, and so did not understand how BounceBack differed from usual primary mental health care. BounceBack was seen as an extension of capacity to deliver (existing) care, rather than a new model of care. As we started to scale up service delivery, we also found that AiW Health caseworkers were struggling to understand their role too. Caseworkers were offering practical support, for example in managing debt, but struggled to support patients and practice staff to recognise a different (flipped) approach to understanding mental health need and so potentially build resilience. At the same time, a new (separate) debt advice service was introduced in to participating practices as part of a separate research study. The effect was to introduce further uncertainty and so inhibit engagement with the new BounceBack service. With very few patients coming in to the service, it was impossible to develop feedback or monitoring processes which supported the continued delivery of the model. Our traffic lights turned to red in all domains. We identified a need for a rapid change of plan.

### Findings solutions: a shift to co-production and development through implementation

With a lack of understanding of, engagement with, and ability to deliver (roll out) the service, it was clear that we needed to refine our description of the BounceBack complex intervention. Our evaluation data gave us insights in to both where change was changed, and what we might do.

We therefore started to use the Normalization Process Theory framework [26] to support a rethink and redesign of the intervention informed by a ‘trial and see’ approach on the ground. We shifted our approach to a process of development through, rather than evaluation of, implementation of the intervention. We refocused our work in a single practice, working closely with the practice team to better define and describe both the core and variable components of the intervention from the whole practice perspective. Data collection continued as described, using the traffic lights analysis framework. The whole process involved a blurring [24] of the previously described boundaries between implementation and evaluation. The University evaluators became part of the service development/integration team and vice versa.

Drawing on a rich data set from observations of our first months in practice, we revised the description of the core component of the intervention (the consultation) to more clearly describe a difference from current alternative care models. We described a specific biographical focus (understanding disruption in the context of the work of daily living), using an unstructured assessment (a narrative based approach rather than the use of symptom/condition measurement tools – to contrast it with the approach used in local Improving Access to Psychological Therapies (IAPT) care), and with a focus on identification of modifiable change (scope for psychosocial actions to support change) (See Table 8).

**Table 8:** Revised Bounce Back Intervention (BB2)

The consultation (core) component of BounceBack ▪ Biographical focus (on the story of disruption) through a narrative (Unstructured) initial assessment (no tools/questionnaires) ▪ Help client explore and understand imbalance of resources and demands (both patient and practitioner) contributing to experienced distress ▪ Support client to identify opportunities for change and support them to do	The organisational components supporting delivery ▪ Sense making: use targeted resources to ensure all parties understand the service ▪ Engagement: Allow direct access (self-referral) and flexible referral patterns to enable patients and staff to engage with the service ▪ Action: Have trained and supported case workers in the practice to deliver the model of care ▪ Monitoring: Feedback the process and impact of care to practice (e.g. case reports and monthly staff meetings)

Based on our revised description of the BounceBack intervention, we also produced a service delivery manual for practices – available from the authors

For the organisational components (needed to support delivery of the consultation), we developed (with the aid of a social enterprise marketing company) and tested a set of resources targeted at each stakeholder group to explain the project and the service (including leaflets, postcards, practice advertising materials, materials for press release). We reviewed and revised our referral (engagement) processes. With case workers now actively involved in the NPT evaluation, this work effectively functioned as a form of training in the model of care, augmented through the additional introduction of clinical supervision. We instituted regular feedback within the BounceBack team, between the team and the practice, and with patients (through continuity of care) (Table 8).

Fifteen months (instead of the planned four) in to the project, we had a service established within seven practices and so recognised the end of Phase one.

## Phase 2: evaluation of delivery

The revised implementation stage left only 6 months for the phase 2 delivery arm of the project. This required us to scale back the planned evaluation of impact and outcomes.

### Methods

Our initial aim was to systematically assess both the process and outcomes of delivery of care using the methods described below.

#### Process evaluation

The purpose of the process evaluation was to describe what was delivered, how and why; in order to support the interpretation of emerging outcome data. We used the case study method described by Yin [36] to assess five elements: context, reach, dose delivered, dose received, acute impact. (Longer term impact could not be assessed given the reduced time for impact evaluation). We considered two levels of delivery: at the patient-practitioner level, and the whole practice level.

### Data collection

Data collection included observation and mini interviews of patients and case workers (four cases for each case worker); review of practice meeting discussion (4 × 1 hour monthly meetings); collection of routine data describing practice demographics and service structure (see appendix G, full case report (http://primarycarehub.org.uk/images/Projects/BB.pdf); numbers of patients referred, attending first and follow up appointments [(http://primarycarehub.org.uk/images/Projects/BB.pdf), page 23].

### Analysis

JR/LC used Yin’s case study approach [36] to describe an analysis framework with which to examine the data. We developed project-specific descriptors for each of Yin’s headings: Context/Reach (who we delivered the service to); Dose Delivered (what service was delivered - did the practice deliver the service and did the caseworkers deliver the described Bounce Back model to clients?); Dose Received (what service patient and practice perceive they had received); Early Impact (what impact did the patient report)? JR/LC then applied the constant comparison method as described within the framework approach [35] to mine the data set for evidence under each of these headings. JR and LC each coded the full data set (including observations, mini interviews, meeting notes – as described above). Given the previously stated limitations of this phase 2 evaluation, both researchers focused on identifying data that described the process of service delivery. We were unable to complete a more in-depth explanatory/exploratory analysis that could explain the observations due to a lack of data. PR and JW were not involved in the analysis of data related to dose delivered/received in order to maintain objectivity, but did help with the analysis of contextual data. The emerging findings were presented at a meeting of the full team to discuss whether/how the findings fitted with the experience of the team delivering the service on the ground. No changes to the analysis were made following this meeting. Rather the discussions informed the emerging recommendations.

#### Impact of service delivery

Our hypothesis was that our new model of care could support improvement in mental health, capacity for daily living (resilience and reduced fatigue) and so engagement with meaningful occupation. We therefore aimed to assess impact using three measures collected at baseline and final consultation: Warwick-Edinburgh Mental Wellbeing Scale (WEMWEBS) [37] – because it was widely used in local service provision so would potentially support between service comparison; the Meaningful Activity Participation Assessment (MAPA) [38] – because enhancing meaningful activity was a local and national priority; Exhaustion – the revised Clinical Interview Schedule CISR20 [39]– because our theoretical model proposed that exhaustion was a risk factor for distress (being a consequence of and/or a contributor to an imbalance between demands and resources [30]).

However problems with data collection (a misunderstanding compounded by the mixing of roles, and a lack of resource) meant we were only able to collect an incomplete data set. The data can be found in the full project report (http://primarycarehub.org.uk/images/Projects/BB.pdf) but are not reported here.

## Results

The full data set is shown in the final project report, available at (http://primarycarehub.org.uk/images/Projects/BB.pdf). Here we present a summary of the key findings.

### Who we delivered care to (reach and context)

Practices were all inner City GP practices, located in areas with moderate to high levels of socioeconomic deprivation. Practices were mixed sizes (from 4200 to 8800 registered patients), with between four and seven GPs working at the practice, and all scoring highly on the Quality Outcomes Framework (pay for performance) General Practice quality measures.

In total, 247 patients were referred in to the service (about half the numbers we had anticipated at the outset). First appointment attendance rate was relatively high (69 %). However, observation data highlighted that patients continued to arrive at their first appointment with only a limited understanding of what to expect from the service. This data also suggested that attendance reflected high levels of need – patients being willing to try anything, including a new service. We saw an expected higher percentage of women than men using the service. But we also noted that a high proportion of our service users (40 %) were men, suggesting that men (a traditionally hard to reach group) recognised our service as appropriate for them. Service users from across a full age range, evenly distributed, were noted to use BounceBack. Patients who did not attend (DNA) their first appointment had similar gender characteristics to those who attended (male patients made up 40 % of first appointment attendees and 36 % of DNA’s; women made up 60 % of first appointment attendees and 64 % of DNAs), supporting our interpretation that the service was recognised as appropriate by both sexes, and all age groups.

### What service was delivered (dose delivered)

At the practice level, two out of the final seven practices referred less than five patients in to the service. This was despite extensive input from the case workers seeking to engage with the practices, explain the service and modify the model to fit with their needs. Staff and patients at these practices expressed ongoing interest in using the service, but this did not translate in to referrals. External factors were observed to play a part in this. For example, practices were upgrading to a new version of the practice software during this time. This was a significant service change which limited capacity for engagement, although the change was happening in all seven practices but only had an inhibitory effect in some. Many changes were happening in primary care at the time of the study and different practices had different capacity and priorities for engagement. In the remaining five practices, referral rates increased over the 6-month period.

At the individual level, the average number of appointments per patient was 2.6. Analysis of case observation data suggested that we were partially delivering the described Bounceback intervention (Table 4). Case workers were good at using a biographical (unstructured assessment) approach to explore the (im)balance of resources and demands on patients. However, case workers less consistently demonstrated supporting the patient in identifying opportunities for change. They found it difficult when patients arrived at the service with a strong medical narrative to explain their distress – a belief that only medical intervention could make a difference to their health concerns. This observation highlighted the importance of patient expectations, and the need to train case workers in managing expectations. It also demonstrated a need to address consistency of approach across a full primary care team (including clinicians referring in to the service) in order to support successful change.

Caseworkers were observed to struggle to go beyond trying to ‘fix’ problems for their clients – to offer more than the traditional AIW model of practical problem solving and instead see their role as helping patients understand their problems differently. This improved over time as case workers developed their understanding of the BounceBack model through its application in practice; enhanced through general critical reflection within the project teams (including as part of the evaluation). In light of feedback from clients and case workers, we also developed targeted resources (leaflets) for use beyond the consultation to help prepare people for, and also reinforce learning from, the BounceBack approach.

### Perceptions of service/care received (dose received) and impact of care

Patients described experiencing the core components of the BounceBack approach – the narrative approach focused on understanding distress and seeking modifiable elements. They described that the service helped by providing support, helping them make sense of things, being prompt and accessible, offering practical solutions, but also helping people develop strategies for doing things differently. The described impact was in helping people make progress, feel better. For example, one participant emailed feedback on their experience of the service:

“I was feeling overwhelmed by the demands made on me…I wanted the service to provide an alternative to medication that would improve the way I felt and my ability to cope…The most useful thing about the service was offering strategies to change thought processes, see things differently and taking some practical action to improve my situation… I would recommend the service because it achieved its aim for me of offering an alternative and practical solution to medication.”

Further examples can be found in the full project report (http://primarycarehub.org.uk/images/Projects/BB.pdf).

Our data demonstrated that for the individuals seen as part of this service development work, we had addressed our goal to help people understand their distress differently, work collaboratively to identify barriers and difficulties in daily living, and so support them to overcome the difficulties. People acquired understanding through engagement with and use of the service. Case study data demonstrates that patients recognised the service as relevant for them and themselves for the service; were able to work with the service (reciprocity) to manage their mental health. There was limited, but early suggestion of potential development of resilience.

## Discussions

In a review of published evaluations of complex interventions, Datta & Pettigrew comment that whilst there is a growing literature describing challenges of complex interventions research, the literature is “thin on practical advice on how these should be dealt with” [14]. Our study offers one contribution to addressing that concern. Our work describes the use of formative evaluation in shaping the development of a complex intervention, highlighting the value of co-production of the emerging intervention through the collaborative effort of researchers and clinician [24].

### Developing and delivering BounceBack – a new complex intervention

Our project described the development of a new complex intervention which we were able to partially integrate into practice. Data suggested that we had moderate success in offering the model of care to patients involved in the project, with some positive impact on their recognition of the service as being useful, their capacity to engage with this way of working (reciprocity), and possibly with developing resilience. We have early signals that we have a new intervention associated with positive outcomes that may be attributed at least in part to the intervention.

Our findings confirmed the NPT predicted need for work across all four domains in order to implement a complex intervention, but went further in showing the need for this work to also refine the development of the intervention. Early and extensive input across the whole service about the nature and purpose of the service was crucial to support sense making. Contextual changes significantly impact on capacity to engage: introduction of a new service needs to be managed as a whole systems change (integrated with external policy and performance management as well as internal practice systems). Resources for delivery were needed across all stakeholders to support continuity of approach across a whole practice team. Monitoring and feedback was crucial to support the refinement as well as the implementation of the intervention. Importantly, our findings highlighted the importance of *continuous* investment – a factor described in NPT but not often highlighted in reports of other studies that have used NPT. The varied and variable context in which we were delivering care demanded flexibility and adaptability. Continuous investment was needed to respond to a changing context but also to develop and evolve a quality intervention.

We set out to contribute to improving primary mental health care using a pipeline approach to translate a research-based complex intervention in to the practice setting. We had assumed a pipeline would work because we had a clear intervention, derived from theory and practice. The pipeline had worked in the past to deliver a new service (for example in the introduction of a Cognitive Behaviour Therapy service run by AiW Health in the primary care setting). However, based on our initial findings, if we had continued to use a pipeline approach in the BounceBack project we would likely have received few referrals, experienced high non-attendance rates and so limited impact. We might have assumed that the model was ineffective.

We finished using scientific method to drive improvement-in-practice-in-context through the generation of practice-based evidence, with researchers and clinicians working together to co-construct and evaluate a new account of practice. Individually tailored care, such as that described by BounceBack, is a complex intervention - one with many parts, and where those parts interact with each other in multiple, and often unpredictable, ways [2]. To respond and intervene in such a dynamic system needs a responsive and adaptive/flexible process. Rather than a pipeline passing knowledge or evidence down the system to be implemented, we need a process that can support generation of knowledge within (and through) adaptation and change. The variability associated with complexity does require a clear vision outlining boundaries of care (what is distinct about the complex care), but also flexibility in developing and applying that model in changing circumstances.

In our BounceBack project, that flexibility came from the capacity to co-create the intervention using the clinical experience of the clinical team with the critical capacity of the academic team to support rigorous knowledge development. The generation of this practice- based evidence involved a blurring [24] rather than a bridging (Fig. 1) between academic and applied contexts in order to support the flexibility needed to respond to changing individual and practice needs. We suggest that the flexible adaptive approach we used is aligned to the model of knowledge utilisation described within a co-production account of complex decision making [40].

BounceBack was an example of what Solberg described as “optimising health and health care through research and quality improvement” [22]. It involved “researchers work[ing] in partnership with practising physicians… on problems identified in practice, using the methods of both practical research and quality improvement” [41]. The result is the generation of practice-based evidence (PBE) – a process of “engaged scholarship” [41] that sees all members of the team contributing to quality improvement.

## Limitations of the study

As previously stated, the evaluation findings related to the delivery and impact of the BounceBack model of care were not intended to be generalised beyond the local setting. This was a service development project. Our understanding of the impact and utility of a new flipped model of primary mental health care delivery can only be achieved by future research including a feasibility study (to assess scalability), and a randomised clinical trial (to assess impact).

However, the findings offer a rich description of the success and problems in using a collaborative approach to formative evaluation as a methodology to support development of a complex intervention. We have highlighted the value (and indeed necessity) of ‘blurring’ the boundaries between clinical and academic staff in developing and implementing the intervention. However, this approach created practical, ethical and interpretive challenges within the second phase of the evaluation. Case workers were asked to distribute quantitative survey tools to patients using the service at key time points. However, they struggled to do this task leading to low response rates and a data set that could not be analysed. We recommend that someone from the research team be responsible for this task in future studies.

Clinical and academic teams had a ‘shared stake’ in the evaluation findings. This raised ethical questions for us as to whether BounceBack case workers were able to give informed consent or dissent to observation. The questions were resolved through discussion, but for future studies we recommend having a separate evaluation team when evaluation moves from a formative to a summative assessment.

Clinical and academic teams had become colleagues over the 15 months of the first phase of the project. We discussed whether in these circumstances, an objective assessment of colleagues’ performance was possible. The observation field work was undertaken by LC who is an experienced mental health worker, as well as a researcher. Observer and observes are used to clinical appraisal of their work by colleagues. We note that our observations did highlight limitations, as well as positive elements, of service delivery. However, again we suggest that future summative evaluation could be strengthened by using an ‘external’ evaluator.

## Implications for future co-production of complex interventions: from pipeline to incubator

Our work highlights the potential to use the complex interventions framework to support the co-production of practice-based evidence through the formative evaluation of the process, merit and worth [32] of innovation and practice. In this study, co-production employed the distinct expertise of both the academic and clinical members of the team. The academic team brought expertise in process of development and interpretation of trustworthy knowledge. The clinical team brought expertise in the process of clinical practice. Together they co-created trustworthy practical knowledge. Both teams worked together in a blurring [24] of traditional boundaries between practice and academia. In this case, our clinical team included the patient perspective since AIW is a patient-led mental health charity. AIW shared the project progress with their user group to invite comment. However, we recognise that co-production could be enhanced in future studies by having a distinct (and separate) patient group as a partner in the process.

**Fig. 2:**
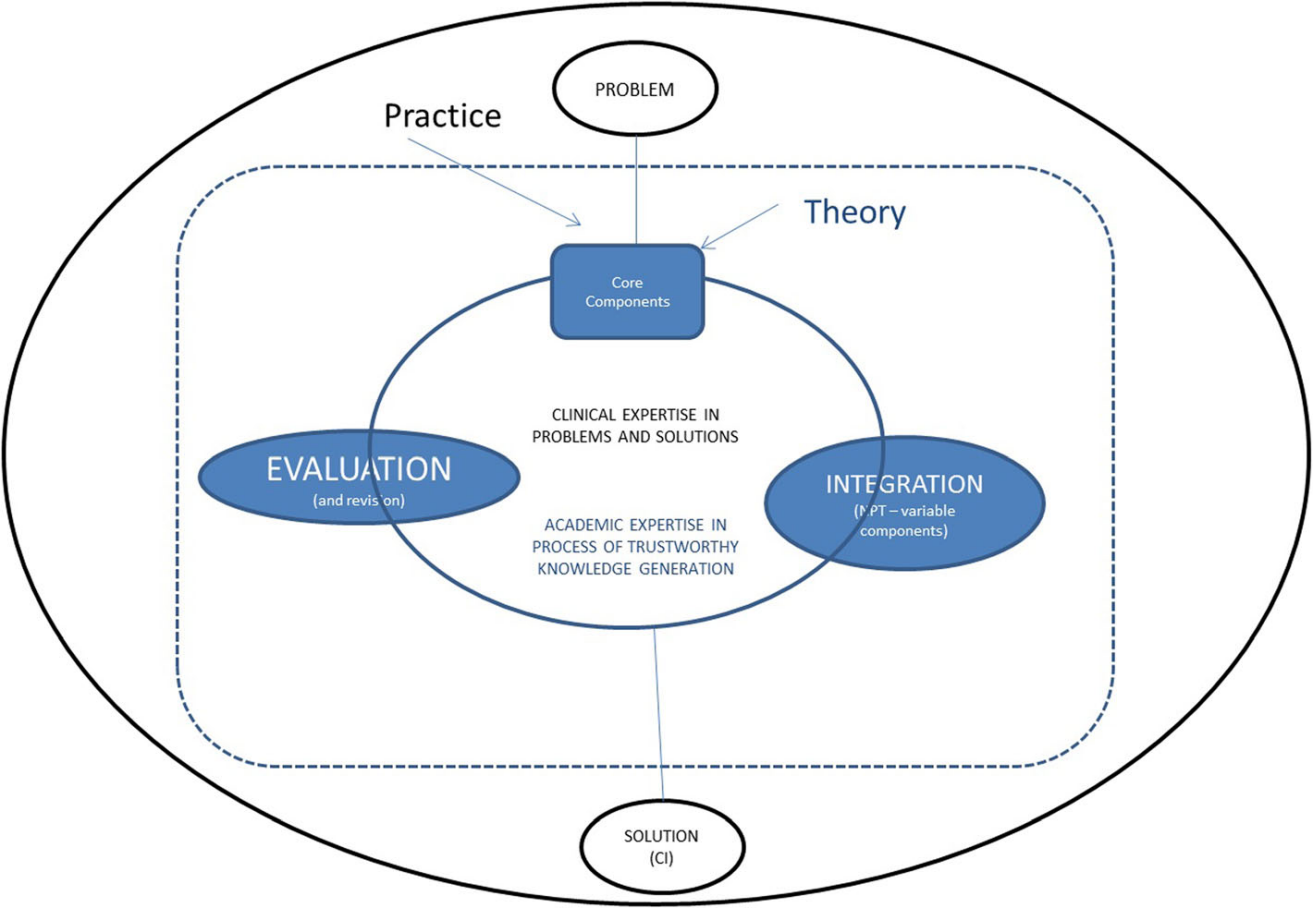
The incubator (co-production) complex interventions model

Our experiences of the added benefit of a co-production approach lead us to propose a change from the pipeline to an incubator (Fig. 2) model for complex intervention generation. Our approach is potentially efficient in making use of all available knowledge (scientific and ‘practical’); and potentially effective in being grounded in the reality and complexity of applied practice. Generating practice based evidence provides a trustworthy account of a practice based view of a ‘way forward’ (options rather than definitive solutions).

We are already using this approach in the development of practice based evidence for generalist management of multimorbidity [42], and with new projects in development on problematic polypharmacy [43] and acute hospital care. But we recognise that an incubator approach to knowledge/evidence generation differs from the traditional pipeline model (Table 7). Solberg also highlights the limitations to this approach in terms of recognition of the different outputs produced, and resources required [22]. Marshall has recognised that co-production of research, with blurring of roles, leads to what some might regard as a less rigorous output [21]. In our case, the loss of rigour was – at least in part – due to a lack of capacity resulting from the extended roles undertaken by academic members of the team. Co-production, including its learning-from-action approach, is also a resource intensive approach. As such, the method itself needs to be critically examined. Co-production of policy decision making has been shown to enhance satisfaction of decisions [40, 44]. We now propose an extension of the model used to analyse satisfaction and quality of decisions and outputs from co-production of knowledge generation through research; in order to critically examine the merit and worth of our proposed model of complex intervention generation.

## Conclusion

Our paper describes the practice-based development of a new complex intervention, BounceBack. We have highlighted the use of Normalisation Process Theory to support development, and not just implementation, of a complex intervention; and described the use of the research process in the generation of practice-based evidence. Our work supports provides a model of supporting practice change through scholarship, and so contributes to future translational complex intervention research.

## Additional files

Additional file 1: Table S4. Showing examples from each of the identified domains and stakeholder groups which illustrate transitions from red to green (August 2013-August 2014). (DOCX 17 kb) Additional file 2: Full evaluation report – includes the full primary data set for the project. (DOCX 10152 kb)

## Abbreviations

NPT: Normalisation process theory
PBE: Practice-based evidence
SIM: Self integrity model
