# Supplementary material for: Developing, delivering and evaluating primary mental health care: the co-production of a new complex intervention
Source: BMC Health Serv Res. 2016 Sep 6;16(1):470. doi: 10.1186/s12913-016-1726-6 (PMC5012043; doi:10.1186/s12913-016-1726-6)
Supplement: Additional file 1: Table S4. — Showing examples from each of the identified domains and stakeholder groups which illustrate transitions from red to green (August 2013-August 2014). (DOCX 17 kb) [file 12913_2016_1726_MOESM1_ESM.docx]

Additional file 1: Table S4: Showing examples from each of the identified domains and stakeholder groups which illustrate transitions from red to green (August 2013-August 2014)

|  | **Stake-holder** | **RED – problems identified** | **AMBER – actions and review** | **GREEN - outcome** |
| --- | --- | --- | --- | --- |
| SENSE MAKING | AIW | Nov-13: AIW case workers still report being focussed on practical case work – seeing their roles as service delivery rather than leading a change in approach/service (*LC observation of case workers in practice)* | Dec-13: AIW staff are actively involved in preparing ‘marketing’ materials – writing the text for study leaflet, podcast, press release. Text that succinctly describes what BB Is and why it is distinct. *(project team meetings)* | May-14: marketing materials done, website finalised. New case worker in post. Team have a clearer sense of what the project aims to do, and what their role is within it. *(project team meetings)* |
|  | Practice Team | Nov-13: Very limited patient referral – staff report (to LC and case workers) that they don’t understand how BB is distinct from usual mental health care, don’t want to disrupt continuity for patients. *Observation of BB in practice, feedback at project meetings* | Jan-14: still very limited referrals *(Project data base, team meeting reviews)*  May-14: referral process in place, but practice staff still confused how BB differs from another 3^rd^ sector service introduced during the project time period (PCAL). | Aug-14: project now functioning in 5 practices (*project data base, team meetings, observation LC)* |
|  | Patients | Nov-13: patients arriving at appointments not knowing why they are there (*case worker report, LC observation)* | Jan-14: working with [a local social enterprise marketing company] to produce a range of ‘marketing materials’ to explain to potential and current service users what the service is doing. Working with User group at AIW to suggest changes | Aug-14: reported findings from case workers that patients arriving with clearer sense of the service. Patients reported that the leaflets (especially the ‘how BB helped me’ leaflet – *available from the authors* - were helpful) |
|  | Policy | Nov-13: meeting with CCG postponed (again) | May-14: meetings with CCG ongoing. Recognition of shared areas of interest, no firm plan for integration as yet | Sept-14: CCG report interest in hearing about our work, notably when have output data |
| ENGAGEMENT | AIW | Nov-13: case workers leading the project, driving forward. But resistance from wider AIW team (don’t understand how BB fits within broader portfolio of work at the charity (including PCAL project). BB seen as a threat) | Sept-14: wider AIW team have better understanding of the BB project, although resistance appears transiently (linked especially to funding changes and insecurity about other projects) | Sept-14: AIW case workers leading the service delivery and the evaluation side (blurring of roles) |
|  | Practice Team | Nov-13: lead GP engaged, other GPs enthusiastic in meetings but not referring patients. Like the idea but not the practice? | Jan-14: identified a local champion (receptionist) within the practice team to drive forward the service  Aug14: now in 6 practices – getting referrals from 5. Some confusion re PCAL still | Sept-14: In 6 practices (though little engagement from one. But given the evaluative nature of the project, decided to keep in the project to learn from) |
|  | Patients | Nov-13: no self referrals, much uncertainty when patients arrive at appointments about why they are there, what service is and is expected of them. | Aug-14: no self referrals, but growing interest from patients, understanding when attend | Sept-14: project is live with steady stream of patients being seen |
|  |  | *LC observation of case workers in practice including mini interviews with staff. Project meetings* | *Reported by case workers at project meetings* | *Project data base* |
| ACTION | AIW | Nov-13: staff training needs identified from LC observation - not offering distinct intervention, rather AIW care. Need for ongoing clinical supervision identified  Dec-13: loss of one case worker, urgently need replacement | April-14: have recruited new case worker. Have started regular supervision for case workers. Case workers engaged in evaluation work – in defining/describing the intervention (supporting own practice) | Sept-14: case workers ready to deliver training to others at planned GP event (was postponed to January 2015) |
|  | Practice Team | Sept-13: GPs not recognising that there is a role for them in doing things differently | May-14: have produced practice/professional and patient leaflets that help people in using BB approach  Identified need for GP training event being planned for Sept 14 | Case workers report that GPs starting to recognise an alternative approach. *To follow in to phase 2* |
|  | Patients | Sept-13: patients arriving with little understanding of their role in care process |  | Case workers report patients working well with the BB model. *To formally evaluate in phase 2 (case studies)* |
|  |  | *LC observation including Case Studies. Project team meetings including with practice staff* | | |
| MONITORING | AIW | Not really happening yet except through project meetings | Project meetings: feedback of NPT traffic lights | *For phase 2* |
|  | Practice Team |  | Case workers feeding back at practice meetings | *For phase 2* |
|  | Patients |  | Patients reflecting back during follow up appointments | *For phase 2* |
|  |  | *Project meetings and the NPT traffic lights* | |  |

Key

*In italics = data source*

BB = BounceBack.

PCAL = another AIW Health commissioned service to provide debt advice in a GP setting
