# Supplementary material for: Developing, delivering and evaluating primary mental health care: the co-production of a new complex intervention
Source: BMC Health Serv Res. 2016 Sep 6;16(1):470. doi: 10.1186/s12913-016-1726-6 (PMC5012043; doi:10.1186/s12913-016-1726-6)
Supplement: Additional file 2: — Full evaluation report – includes the full primary data set for the project. (DOCX 10152 kb) [file 12913_2016_1726_MOESM2_ESM.docx]

**NIHR Innovation fund:
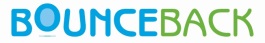
 Project.**

**Evaluation Report, March 2015**

The**
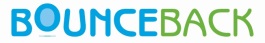
**project was formed by a collaboration between AiW Health (a Mersey-based mental health charity) and the University of Liverpool. The project ran from April 2013 – March 2015 and was funded by an NIHR IESDF grant (2532287, co-applicants: Harrington, AiW Health, and Reeve, Liverpool University). The team consisted of the grant holders plus two case workers employed by AiW Health (Watkins and Rosbottom), and a University research fellow (Cooper). At the outset, AiW Health was the lead partner in delivering the service and Liverpool University in evaluating the service. As described in the report, these roles changed over the duration of the project. Our evaluation report describes the three main phases of the Innovation project: set up, delivery, and dissemination; and considers the implications for future work.

**Overview**

**
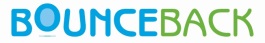
** aims to challenge and change current thinking about how to assess mental health and wellbeing in a primary care setting through the (re-)introduction of a generalist, person-centred model of care.

Current primary care mental health services have been designed to offer a disease-focused (specialist) model of mental health needs assessment. Initial assessment considers does this person meet the criteria for a diagnosis of depression. A severity score (assessed using standardised measures) determines what help they will receive (e.g. talking treatments or medication). Help may include practical support to address problems that can limit or slow recovery, but practical support is effectively a second line intervention.

Twenty years’ experience at AIW Health suggests a different model of care can be helpful for some. The AiW Health approach focuses on addressing practical issues first, with medicalisation used as a back- up if needed, or if additional risks are identified. Research evidence also highlights concerns that an overly medicalised approach to understanding distress results in overdiagnosis of some but under recognition of need in others. The ‘flipped model’ approach used by AIW Health (whereby a medical model is use as a 2^nd^ line) is supported in principle by a wider body of research, but there is limited formal evidence to support a shift in health policy and practice.

The **
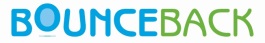
**project used a complex interventions approach to develop, deliver and evaluate the impact of a new intervention – a person-centred (generalist) model of mental health care.

- We described our initial intervention based on the AiW Health model, informed by current research
- We integrated our new intervention in to the primary care setting by working with local primary care stakeholders, using an approach informed by Normalisation Process Theory (Phase 1)
- We refined our description of the intervention based on what we learned from Phase 1 about the barriers and enablers to introducing our intervention
- We rolled out our revised intervention into new practices to deliver the service and evaluate the process of delivery, and the impact of the intervention (Phase 2)
- We shared our learning from the project with local GPs at a GP education event

Our translational research approach was key in generating new knowledge about an intervention, but did introduce limitations in terms of the nature of the evidence we were able to produce.

In this report, we describe the work we did in each of these steps and what we learnt. We conclude by drawing together our findings to describe:

- A new **
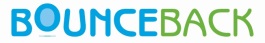
**Socio-Psycho-Bio model of mental health care
- Next steps needed to develop the formal evidence base for this way of working and so contribute to developing practice and policy.

**Contents**

|  |  | **Page** |
| --- | --- | --- |
|  | **Overview** | **2** |
|  | **Contents** | **3** |
| **1.** | **Introduction and Background** | **4** |
| 1.1 | The problem: why we need innovation | 4 |
| 1.2 | Our approach: establishing the BounceBack project | 4 |
| 1.3 | The original BounceBack care model | 6 |
| **2.** | **Project Aims and Objectives** | **7** |
| 2.1 | Aims | 7 |
| 2.2 | Ethics and governance | 8 |
| **3.** | **Phase one: Integrating BounceBack in to Primary Care** | **8** |
| 3.1 | Describing our approach: theory informing our work | 8 |
| 3.2 | A revised Integration Protocol using NPT toolkit – our method | 10 |
| 3.3 | Describing our results/Outcomes | 13 |
| **4.** | **Phase Two: Evaluating the impact of Bounce Back** | **17** |
| 4.1 | Overview and Aims | 17 |
| 4.2 | Evaluation data collection and Analysis plan | 17 |
| 4.3 | What we found | 21 |
|  | 4.3.1 Service integration and implementation | 21 |
|  | 4.3.2 Process of service delivery | 23 |
|  | 4.3.3 Impact of care | 26 |
| 4.4 | What we learnt | 27 |
| 4.5 | Implementing our learning | 27 |
| 4.6 | Conclusions | 30 |
| **5.** | **Dissemination** | **30** |
| 5.1a | Shared reflections | 30 |
| 5.1c  5.2 | Shared learning  Further planned dissemination | 30  31 |
| **6.** | **Overall Reflections** | **32** |
| 6.1 | Summary of outputs | 32 |
| 6.2 | Strengths and Limitations of the work | 32 |
| 6.3 | Next steps | 33 |
|  | **Author Details and contacts** |  |
|  | **References** | 33 |
|  | **List of Tables and figures**  **Appendices** | 35  35 |

1. **INTRODUCTION AND BACKGROUND**

**1.1 The problem: why we need innovation in primary mental health care**

Depression is a leading global cause of disability.^1^ In the UK, *No Health without Mental Health* (2011) set out the government’s commitment to improving mental health and addressing inequalities in mental health.^2^ The report acknowledges recent progress made in service provision but highlights the need to ‘go further’. In particular to address concerns that not everyone has benefited equally from service improvements to date.

Poor mental health disproportionately affects the most vulnerable members of society: e.g. those with long term conditions, from deprived socioeconomic backgrounds, and the unemployed. There is evidence of a persisting Inverse Care Law.^3^ A recent programme of research (the AMP project) provided evidence that issues of access to services contribute to inequalities and poor service provision.^4^ Importantly, access issues are related to the *nature* and not just the availability of care. Overreliance on a biomedical account of mental illness contributes to inequalities in health through problems with access that go beyond availability – described by Kovandžić (2012)^5^ as candidacy, concordance and recursivity (see Box 1). Authors have called for a need to recognise going beyond a medical approach to understanding mental health problems in order to improve care, and address inequalities.^5,6^

BOX 1: Understanding Access (adapted from ^5^)

Improving equity of access to care needs to ensure services are able to support three elements to necessary for equitable navigation of, and access to, care

- Candidacy – whether the individual recognises themselves as eligible/suitable for the service and vice versa
- Concordance – whether the individual is successfully able to work with the service to address their health problems (whether the service matches needs)
- Recursivity – whether the service leaves the individual with (enhanced) capacity to deal with similar problems in the future

Leaving us with two challenges: firstly to in describe how we deliver a demedicalised mental health care model , and secondly in evaluating the impact of change.

**1.2 Our Approach: establishing the
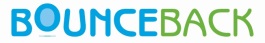
project**

Discussions amongst our team identified a shared goal to address the growing burden of mental health problems within the community by developing innovative approaches to access to care in the primary care setting. The result was the **
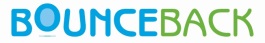
**project.

The project is a collaboration between the charitable sector, existing health services and the academic community. AiW Health is a charity with twenty years’ experience of delivering person-centred mental health support to people experiencing distress.^7^ They recognise that practical issues are often the primary factor(s) in many people’s mental health problems. Their approach to understanding and addressing mental health need flips the traditional medical model on its head. Rather than a health professional assessing/diagnosing mental illness and then referring on for support for wider concerns, AIW Health does the reverse. Care starts in AiW Health with a non-biomedical needs assessment delivered by an AiW Health worker. Only if mental health issues remain despite having addressed wider social support/practical issues is a biomedical model employed. Service users and members of the public have both reported that the AIW Health approach describes a service they would want to use. Anecdotal evidence from the Charity suggests that this approach might address highlighted concerns about access.

The AiW Health model resonates with the academic Self Integrity Model (SIM) developed by Reeve.^8^ SIM recognises disabling distress as resulting from an imbalance between the demands on and resources available to an individual in maintaining daily living. Mental healthcare need is understood as a the intervention necessary to address the health related imbalance. Within the SIM approach, medical accounts of depression and anxiety are one of several potential resources which can be used to understand and address distress. The role of the generalist medical practitioner is to understand when it is, or isn’t, in this individual’s best interests to medicalise their distress in order to restore health related capacity for daily living.^9^

AiW Health’s model is an empirical one, built from practical experience. SIM is a theoretical one, built from empirical research. Discussions between Harrington and Shaw (AIW Health) and Reeve (Liverpool University) recognised the overlap between the two models, and their potential to support a service redesign which addresses the highlighted issues about access. With both theoretical and practical support for a ‘flipped model’ of care, we successfully bid for NIHR IESDF funding to support the introduction into a primary care setting, and formal evaluation of this new way of working. Our proposition being that a flipped model might improve individual mental health outcomes, and mental healthcare through addressing access issues of candidacy, concordance and recursivity.^5^ The essential elements of our original **
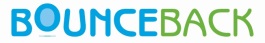
**model are shown in Box 2. Details of our original bid can be found in Appendix A.

**1.3 The original
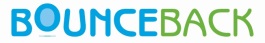
model of care**

| Box 2: The original **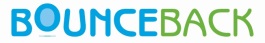**Intervention (version BB1)  APPROACH   - Adopts a person centred understanding of distress, resulting from an imbalance between resources and demands - Explored and understood through open conversation focused on the patients experience - Identifying potentially remediable gaps in (practical) support in order to identify action points   DELIVERY   - Delivered by AIW Health case workers embedded into the primary healthcare team - First assessment visit supports formulation of an action plan - Follow up until practical problems limiting daily living and engagement with meaningful occupation addressed - Resilience/forward planning meeting once immediate issues resolved, to consolidate learning (dealing with future problems), action plan for maintenance, and future contact route if needed. - Recorded in the practice records to support integration with the clinical team |
| --- |

1. **PROJECT AIMS & OBJECTIVES**

**2.1 Aims**

Our research question asked: can integration of an alternative demedicalised model of mental health care improve access to, and outcomes from, primary mental health care?

We had 3 overall project AIMS:

1. to *integrate* the **
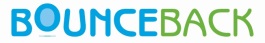
** model into a primary care (general practice) setting
2. to *apply* the model and deliver care over a 12month period, supported by an integrated evaluation to identify learning and evaluate benefit for patients, primary care teams, health services, and communities;
3. to *inspire* wider change in service design and provision through disseminating learning through conversations with commissioners, education programmes, community groups.

The project was planned in two phases. Phase One was concerned with embedding the new way of working into Primary Care (0-4months). Phase Two focused on delivering the innovative service to patients. Both phases included embedded evaluation (See Figure 1).

**Figure 1: Original timeline for the
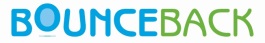
project**

|  | 0-3m | 3- | 6- | 9- | 12- | 15- | 18- | 21-24m |
| --- | --- | --- | --- | --- | --- | --- | --- | --- |
| **INNOVATION** | | | | | | | | |
| Integration | 0-4m: embed in practices, train GPs |  | | | | | | |
| Application | Train AiW staff, review protocols and support | Run service: 6 practices, 2 AiW case workers (see 2.3) - 5-17months | | | | | AiW staff - analysis and dissemination with service wind up | |
| Dissemination | Awareness raising |  | | | | | Share learning including conference | |
| **EVALUATION** | | | | | | | | |
| Evaluate embedding | Observation (JR) |  | | | | | | |
| Baseline practice data | Practice data (practices) |  |  |  |  |  |  |  |
| Pre and post intervention scores |  | Survey tool using described measurement tools delivered to clients by AiW staff | | | | | Continue data collection for reduced service | |
| Patient case studies |  | | Longitudinal observation of purposively selected case studies (by researcher) | | | |  | |
| Process service delivery |  | Comparative observational study of the process of service delivery (AiW and GP) – researcher | | | | |  | |
| Analysis outcome measures |  | | | | Analysis before-after change (JR and researcher) | | |  |
| Analysis process data (NPT) |  | | | | | | Process data evaluation (JR) | |
| Economic modelling |  | | | | | | | Health ec team |

- 1. **Ethics and governance**


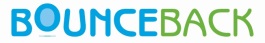
describes an innovative service development project – the introduction of a new service accompanied by an evaluation. As defined by the NHS Research Ethics guidance, the work is service development not research.^10,11^ ‘For the purposes of research governance, research means the attempt to derive generalisable new knowledge by addressing clearly defined questions with systematic and rigorous methods’.^10^ Our work describes an exploratory service development project with embedded evaluation of the service. The specific findings were not intended to be generalisable beyond the context (which would have required different sampling, introduction of a control phase and so on). Rather the project was committed to an active learning process through continuous critical review to maximise the learning from the project, and so inform development of a subsequent formal research proposal.

We confirmed our interpretation through consultation with Dr Peter Klimiuk (Chair, Northwest 10 Research Ethics Committee Greater Manchester North), and with Gabbie Marr (Research Governance manager Liverpool & Sefton PCTs). We approached Liverpool University Research Ethics Committee to seek review/approval for the work but were advised that NHS service development projects also fell outside of their remit. Nonetheless, both researchers were fully GCP trained and we followed strict rules of good research governance. Case workers were supported to adhere to clinical governance best practice by individual practices.

1. **PHASE ONE: INTEGRATING**
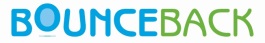
**INTO PRIMARY CARE**

Having described our model of care (Box 2, page 6) and secured funding, we set out to introduce **
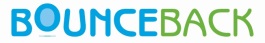
**care into General Practices in Liverpool. By working collaboratively with practices, we aimed to explore and address enablers and barriers to integration. Allowing us to refine our intervention and so describe a service delivery manual.

Goals for Phase One (see also Figure 1)

- To appoint staff: to appoint and train 2 case workers
- To identify partner practices (aiming for 6 in Liverpool)
- To establish case workers in practice
- To start delivering the **
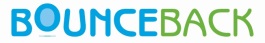
** service to patients

**3.1 Describing our approach: the theory informing our work**

We used the MRC Complex Interventions Framework^12^ and Normalisation Process Theory^13^ to help us develop our work.

We described our **
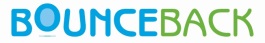
**model as a **Complex Intervention**.^12^ A complex intervention is one which has multiple component parts, including some core/constant elements (which make it distinct) and variable elements (which make it flexible/adaptable enough to work in practice – in different settings, and for different people).^14^

In our study, the CORE elements are those shown in Box 2, page 6 – the essential elements that (from theory and applied practice) we believed needed to be in the intervention. The VARIABLE elements are the context specific factors needed to make our approach work in applied practice. From experience, we already had some ideas of what these would be (as outlined in our original bid, see Appendix 1). The goal of Phase One was to systematically identify these variable components through a process of translational research, informed by Normalisation Process Theory.

- - 1. Introducing Normalisation Process Theory (NPT)

NPT is a theory used to understand how to implement a complex intervention in to a new setting.^13^ NPT tells us that for a new way of working to be successfully integrated into everyday/usual practice, there needs to be continuous investment in 4 areas of work: Sense Making, Engagement, Action and Monitoring (see Box 3).

We intended to use these four concepts to inform our conversations with practices about introducing our new service. Joanne Reeve (JR) would also use the framework to evaluate the progress of integration.

| Box 3: Describing the four key concepts in Normalisation Process Theory^13^  **S**ENSE MAKING: people must individually and collectively understand what the new way of working is; how it is different from what went before; and why it matters (what the benefits are)  **E**NGAGEMENT: people must agree to start doing the new model of care, and continue working at it  **A**CTION: people need to have the resources to work in the new way  **M**ONITORING: people need to get feedback that reinforces the new way of working |
| --- |

- - 1. Initial scoping work – early recognition of a need for change

Our original timeline included an ambitious plan to integrate the new service in just 4 months (Figure 1, page 7). This was based on earlier conversations between AIW Health and local GPs in which practitioners expressed a desire to have AIW Health come in to practices to deliver care. In the first months of the project, we appointed a case worker (Helen Washington, HW) who was able to start her training by seeing patients initially at AIW Health. HW also worked with Sean Harrington (SH) to identify potential practices in which we might implement the new service. HW and SH used the NPT principles to inform their work: in having a clear description of a new service (supporting sense making), in scoping sites expressing need (a desire to engage), and in offering resource to deliver the service (case workers).

Start-up was slow, with a number of unanticipated barriers to project integration. These included delay in appointing a second case worker, the start of a new primary care research project in Liverpool examining the impact of debt management on mental health, and the introduction of changes to local strategic planning in mental health care provision. Crucially it became clear that AIW Health would need more support in the integration side of the project – that maintaining separate Innovation and Evaluation streams (Figure 1) was not going to work. Rather than an evaluation stream working to monitor the integration stage, we instead recognised a need to use the evaluation approach to *inform and develop* implementation.

With recognition of a need for an expanded role for the evaluation team, the University was able to secure some additional time for a researcher, Lucy Cooper (LC), to support JR^[[1]](#footnote-1)^ in providing support to the whole project – thus integrating the evaluation and innovation streams to support successful delivery of Phase 1.

To do this, we switched to using the Normalisation Process Theory toolkit^15^ as a framework to support (rather than monitor/evaluate) integration.

- 1. **A revised Integration protocol using the NPT tool kit – describing our method**

3.2.1 Our approach: implementing the NPT toolkit

Drawing on Normalisation Process Theory (NPT), we could predict that successful integration would need all stakeholders* to be able to make sense of the new approach and engage with it, have (access to) the resources to deliver it, and receive feedback to support the ongoing work.

*Our recognised stakeholders included: practices, patients, and policy makers/commissioners. To this list we also added AIW Health, as it became apparent that lack of understanding of this project within the wider AIW team was also a barrier to working.

NPT includes an NPT Toolkit^15^ designed to support teams in systematically identifying progress in integration/implementation across the 4 areas of work (Box 3). Each area of work has 4 subthemes or specific areas of practice (see Figure 2). NPT thus describes 16 pieces of work we needed to explore across 4 groups of stakeholders. We had 64 areas of work which we potentially needed to explore and address in order to support integration of **
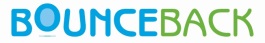
**into the primary care setting. We used the chart shown in Figure 2 as a framework to both support our project planning (to identify potential work we needed to do to support integration [2a], and to monitor our progress [2b]).

- 2a. PROJECT PLANNING: At team meetings, we discussed options for work in each of the target areas to support integration. For example, we recognised a need to be able to quickly and simply describe the aims of the project (sense making) to a range of different audiences (patients, practitioners, policy makers). We thus set a target to prepare briefing materials for each stakeholder group ready for the next project meeting
- 2b. PROJECT MONITORING: AIW staff fed back examples of work done (successfully or otherwise) in each of the themes – via email, at team meetings. LC also observed AIW staff in practice to record areas of progress and problems

Data sources therefore included: record of project meetings, observation of practice (by LC) including meetings with practices and working with patients, mini interviews with staff and patients during observation stage (LC), review of service database (record of patients numbers through the service held by AIW IT staff).

We used a traffic lights system to chart these data and monitor our progress in each area over time. Areas of work were coded as green if our data collection/observations documented successful progress in this area; red if we had no data/evidence of progress towards integration; and amber if the data were patchy.

We used the traffic lights framework to review our progress at monthly project meetings, where we flagged up areas of concern, feeding monitoring/evaluation data back in to the project planning process to support setting of monthly goals.

|  | (2a) Data/Evidence we need  (Used to plan monthly activities) | | | | (2b) Timeline: October 2013  (Charting progress using traffic light system) | | | |
| --- | --- | --- | --- | --- | --- | --- | --- | --- |
| **Figure 2: Traffic light framework template** | AiW | Practice | Patient | Policy | AiW | Practice | Patient | Policy |
| **SENSE MAKING** |  |  |  |  |  |  |  |  |
| 1. All stakeholders can describe what the service is and how it is new (innovative) |  |  |  |  |  |  |  |  |
| 2. All stakeholders have a shared understanding of what the service aims to achieve |  |  |  |  |  |  |  |  |
| 3. Each individual knows what they need to do: their own specific tasks and responsibilities |  |  |  |  |  |  |  |  |
| 4. All stakeholders know why a person-centred, non-medical approach to mental health needs assessment and care matters |  |  |  |  |  |  |  |  |
| **ENGAGEMENT** |  |  |  |  |  |  |  |  |
| 5. Project champions are in place and driving the project forward |  |  |  |  |  |  |  |  |
| 6. Other stakeholders agree to join in to drive the project forward |  |  |  |  |  |  |  |  |
| 7. Other stakeholders are willing and able to start doing the work |  |  |  |  |  |  |  |  |
| 8. Other stakeholders are willing and able to continue doing the work |  |  |  |  |  |  |  |  |
| **ACTION** |  |  |  |  |  |  |  |  |
| 9. All stakeholders are actually doing their individual work for the project (descriptive)? |  |  |  |  |  |  |  |  |
| 10. Is everybody working together to make the project happen? |  |  |  |  |  |  |  |  |
| 11. All stakeholders have the necessary skills and resources to do their part of the work |  |  |  |  |  |  |  |  |
| 12. The intervention is adequately supported by its host organisation |  |  |  |  |  |  |  |  |
| **MONITORING** |  |  |  |  |  |  |  |  |
| 13. All stakeholders get feedback about the impact of the intervention |  |  |  |  |  |  |  |  |
| 14. As a result of the feedback, there is a collective (all stakeholders) sense that the project is worthwhile |  |  |  |  |  |  |  |  |
| 15. As a result of the feedback, individuals think the project is worthwhile |  |  |  |  |  |  |  |  |
| 16. All stakeholders are able to learn from the feedback in order to modify and improve the future running of the project |  |  |  |  |  |  |  |  |

- - 1. Charting progress

The traffic light framework was presented at regular project meetings for the full team to review. As shown in Figure 3, initial progress was encouraging. However it quickly became apparent that we weren’t adequately engaging all stakeholders and in all the areas required. Our traffic lights started to turn from green/amber to red.


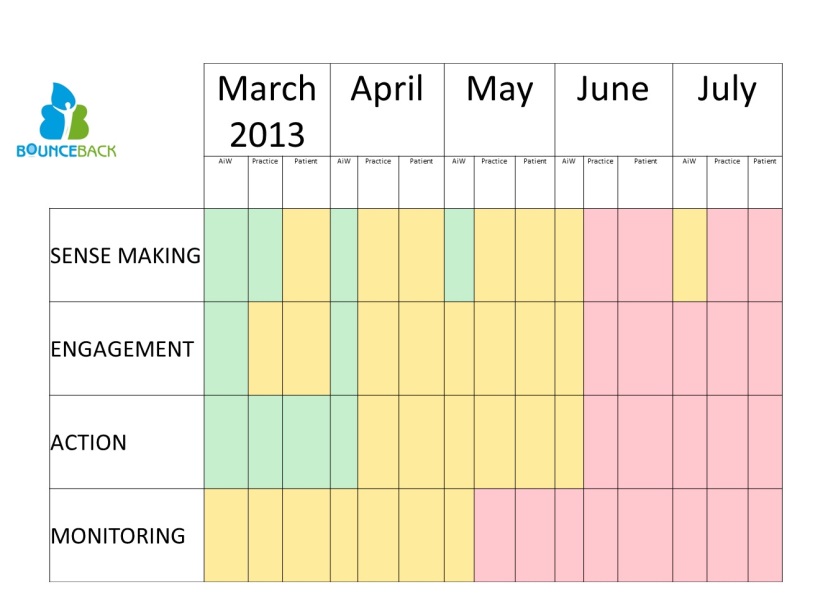
Figure 3: Progress March to July 2013 – progression into the red

We made a number of changes in response. We changed to weekly team meetings and planning reviews. Each meeting discussed progress (or lack of it) in each area, identified plans for actions to overcome barriers, and reviewed the impact of previous changes. Over time, our traffic lights once more returned to green (Figure 4).


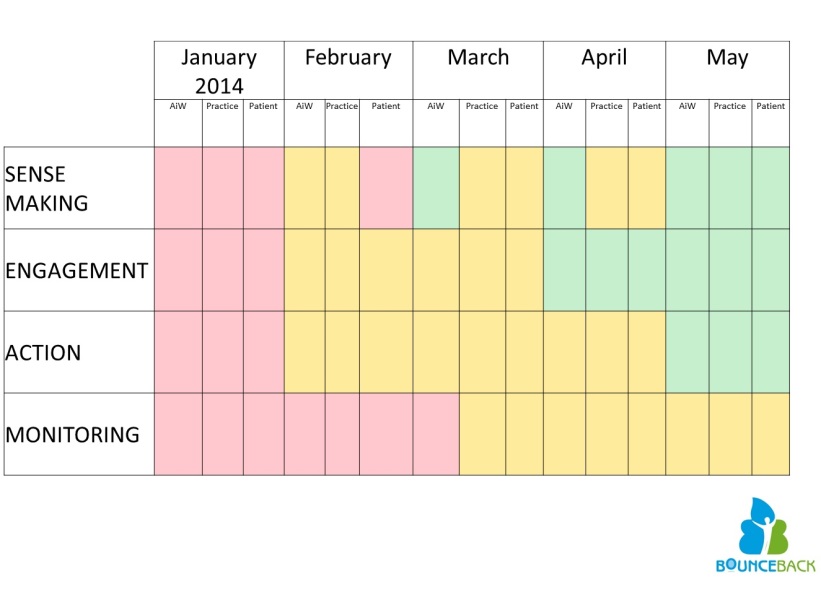
Figure 4: Progress 2014 – and back in to the green

3.3 **Describing our results**

Our full data set is large and complex. A detailed narrative of the way we made changes is given in APPENDIX B. Here we offer some examples from the full data set of situations coded as red or amber, to show specifically how we were able to identify and address the necessary changes and so transition to green. (BB = **
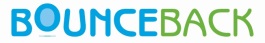
**)

**Table 1: Examples of actions arising from traffic light review (August 2013- August 2014)**

*(In italics – data sources used)*

|  | **Stake-holder** | **RED – problems identified** | **AMBER – actions and review** | **GREEN - outcome** |
| --- | --- | --- | --- | --- |
| SENSE MAKING | AIW | Nov-13: AIW case workers still report being focussed on practical case work – seeing their roles as service delivery rather than leading a change in approach/service (*LC observation of case workers in practice)* | Dec-13: AIW staff are actively involved in preparing ‘marketing’ materials – writing the text for study leaflet, podcast, press release. Text that succinctly describes what BB Is and why it is distinct. *(project team meetings)* | May-14: marketing materials done, website finalised. New case worker in post. Team have a clearer sense of what the project aims to do, and what their role is within it. *(project team meetings)* |
|  | Practice Team | Nov-13: Very limited patient referral – staff report (to LC and case workers) that they don’t understand how BB is distinct, don’t want to disrupt continuity for patients | Jan-14: still very limited referrals *(Project data base, team meeting reviews)*  May-14: referral process in place, but practice staff still confused how BB differs from another 3^rd^ sector service introduced during the project time period (PCAL). Moving to new practices | Aug-14: project functioning in 5 practices (*project data base, team meetings, observation LC)* |
|  | Patients | Nov-13: patients arriving at appointments not knowing why they are there (*case worker report, LC observation)* | Jan-14: working with Artemis to produce range of ‘marketing materials’ to explain to potential and current service users what the service is doing. Working with User group at AIW to suggest changes | Aug-14: reported findings from case workers that patients arriving with clearer sense of the service. Patients reported that the leaflets (especially the how BB helped me leaflet – C3, p14-15 - were helpful) |
|  | Policy | Nov-13: meeting with CCG postponed (again) | May-14: meetings with CCG ongoing. Recognition of shared areas of interest, no firm plan for integration as yet |  |
| ENGAGEMENT | AIW | Nov-13: case workers leading the project, driving forward. But resistance from wider AIW team (don’t understand how BB fits within broader portfolio of work at the charity (including PCAL project), seen as a threat) | Sept-14: wider AIW team have better understanding of the BB project, although resistance appears transiently (linked especially to funding changes and insecurity about other projects) | Sept-14: AIW case workers leading the service delivery and the evaluation side (blurring of roles) |
|  | Practice Team | Nov-13: lead GP engaged, other GPs enthusiastic in meetings but not referring patients. Like the idea but not the practice? | Jan-14: identified a local champion (receptionist) within the practice team to drive forward the service  Aug14: now in 6 practices – getting referrals from 5. Some confusion re PCAL still | Sept-14: In 6 practices (though little engagement from one. But given the evaluative nature of the project, decided to keep in the project to learn from) |
|  | Patients | Nov-13: no self referrals, much uncertainty when patients arrive at appointments about why they are there, what service is and is expected of them. | Aug-14: no self referrals, but growing interest from patients, understanding when attend | Sept-14: project is live with steady stream of patients being seen |
|  | *Data sources* | *LC observation of case workers in practice including mini interviews with staff. Project meetings* | *Reported by case workers at project meetings* | *Project data base* |
| ACTION | AIW | Nov-13: staff training needs identified from LC observation - not offering distinct intervention, rather AIW care. Need for ongoing clinical supervision identified  Dec-13: loss of one case worker, urgently need replacement | April-14: have recruited new case worker. Have started regular supervision for case workers. Case workers engaged in evaluation work – in defining/describing the intervention (supporting own practice) | Sept-14: case workers ready to deliver training to others at planned GP event (was postponed to January 2015) |
|  | Practice Team | Sept-13: GPs not recognising that there is a role for them in doing things differently | May-14: have produced practice/professional and patient leaflets that help people in using BB approach  Identified need for GP training event being planned for Sept 14 | Case workers report that GPs starting to recognise an alternative approach. *To follow in to phase 2* |
|  | Patients | Sept-13: patients arriving with little understanding of their role in care process |  | Case workers report patients working well with the BB model. *Toformally evaluate in phase 2 (case studies)* |
|  | *Data sources* | *LC observation including Case Study 2. Project team meetings including with practice staff* | |  |
| MONITORING | AIW | Not really happening yet except through project meetings | Project meetings: feedback of NPT traffic lights | *For phase 2* |
|  | Practice Team |  | Case workers feeding back at practice meetings | *For phase 2* |
|  | Patients |  | Patients reflecting back during follow up appointments | *For phase 2* |
|  | Data source | *Project meetings and the NPT traffic lights* | |  |

3.3a APPENDIX D summarises all the work done in Phase 1 which led to successful integration (set up) of the project in our pilot practice (P1) and hence roll out into all 7.

3.3b Key lessons learned from this process:

- The importance of developing a shared vision with all stakeholders involved in developing a new Complex Intervention: the collective process of developing the vision supports engagement, sense making and development of actions; the vision itself supports actions (delivery) and monitoring
- The need to modify expectations and actions of service, practice and evaluators to make change happen: negotiating a process of compromise – which takes time
- Feedback matters, and needs to be a continuing process: because circumstances change
- This complex process was achieved only by a process of *blurring^16^* the boundaries between service delivery and evaluation
- This work took longer than anticipated (15 months instead of 4)

3.3c The outcomes of this work was, however

- A revised description of the Complex Intervention (see Box 4)
- A manual describing how the service might be delivered in the General Practice setting (see APPENDIX E)

| **Box 4 – Revised Bounce Back Intervention – BB2**  The pilot and developmental stages of our project (Phase 1) helped to further develop and refine our understanding of the intervention. | |
| --- | --- |
| **The consultation model (core elements)**   - Biographical focus (on the story of disruption) - Unstructured initial assessment (no tools/questionnaires) - Explore and understand imbalance of resources and demands (both patient and practitioner) - Support client to identify opportunities for change and support them to do | **The organisational context (variable components)**   - S: use targeted resources to ensure all parties understand the service - E: Allow direct access (self-referral) and flexible referral patterns to enable patients and staff to engage with the service - A: Have trained and supported case workers in the practice to deliver the model of care - M: Feedback the process and impact of care to practice (eg case reports and monthly staff meetings) |
| **See Appendix E for manual describing specific delivery** | |

And so, later than anticipated, we were ready to undertake an evaluation of impact in Phase 2 of the project.

**PHASE TWO: EVALUATING THE IMPACT OF
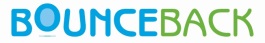
**

**4.1 OVERVIEW AND AIMS**

As the set up time for this project took much longer than anticipated, we had a much reduced time frame for Phase 2. We therefore rewrote our evaluation plan

Our revised aims were:

- to deliver care to clients over a 6 month period (Sept 2014-Feb 2015)
- to measure the impact of care on personal outcomes
- To evaluate the process of care using case study analysis to explain observed outcomes/change
- to describe the feasibility of integrating/running the intervention in new settings

All with a view to informing an application for funding for a pilot into full randomised trial.

**4.2 Evaluation data collection and analysis plan**

Figure 5 shows an overview of the revised evaluation plan for Phase 2, with 3 components to evaluate: the Service Integration; the Process of Delivery and the Outcomes of Delivery

**Figure 5: Evaluation strategy overview**

**
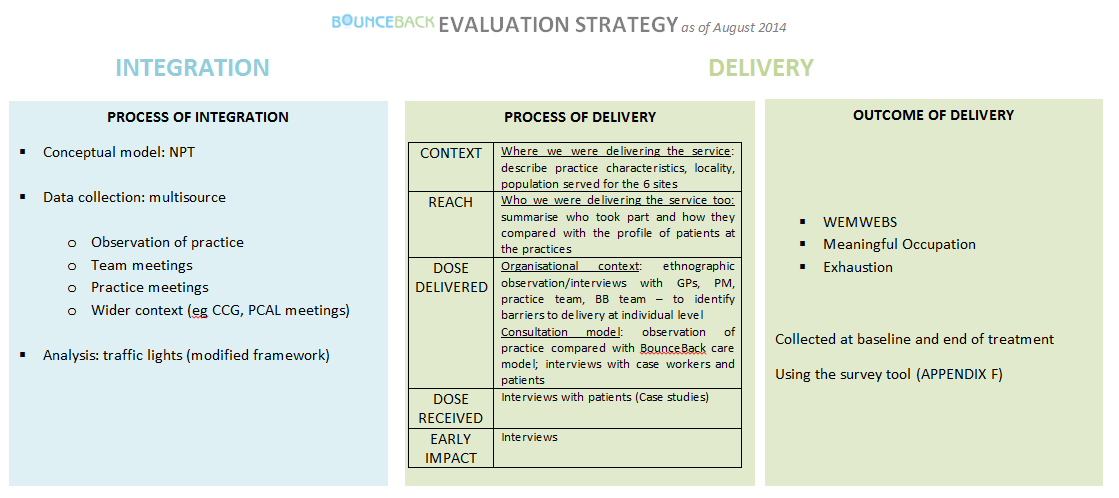
**

4.2.1 *Service Integration*

- Using the NPT framework^15^ described in Phase 1 to examine/identify new (unexpected) barriers to service roll out from our set up practice to the additional 6 practices
- Using data collection methods including observation, mini-interviews, and email survey in the 6 practices
- Analysed using the framework approach described in Phase 1

4.2.2 *Process of Service Delivery*

- Using the case study method described by Yin^17^ and summarised in Figure 5.
- Details of planned data collection are shown in Table 2 (next page)
- Thematic analysis to describe:
  - who we delivered the service to (Context/Reach)
  - what service was delivered: did the caseworkers deliver the described Bounce Back model to clients? Did the service context support or inhibit the delivery of Bounce Back care to the individual? (Dose Delivered)
  - what service did the patient perceive they had received: did the patient report having received Bounce Back care (Dose Received)
  - what impact did the patient report: Early Impact

And so describe whether we had been able to deliver the service, and what if any further enablers/barriers to delivery were identified

4.2.3 *Impact of Service Delivery*

Our hypothesis was that our new model of care could support improvement in mental health, capacity for daily living (resilience and reduced fatigue) and so engagement with meaningful occupation. In Phase 1, we piloted the use of a number of outcome tools including PHQ9 (mental health score, used commonly in the primary care context), SF12 (health and wellbeing measure), Meaningful Activity Participation Assessment (MAPA), and CIS20R (a measure of fatigue). Feedback from the pilot stage, including from User group review, was that the survey tool (incorporating all of these scores) was too long.

During the time of the project, there was a shift in local mental health services to a greater use of the Warwick-Edinburgh Mental Well Being Scale (WEMWEBS) as a mental health outcome measure.

By this stage, we also had reduced researcher time available to support outcome data collection.

We therefore significantly revised the outcomes data collection survey tool to collect 3 measures: of mental health status, of exhaustion (a risk factor for not being able to engage in daily activities), and of meaningful occupation. The final 3 measures selected were (see APPENDIX F):

- Warwick-Edinburgh Mental Well Being Scale (WEMWEBS)^18^
- Meaningful Activity Participation Assessment (MAPA)^19^
- Checklist Individual Strength (CISR20) ^20^

Case workers were asked to give the survey tool to their clients at the first meeting, and at the final meeting – if necessary posting out to clients. Results were to be returned to a named worker at AiW Health who would maintain a secure database with details of all clients who were seen, demographic data (age, gender, postcode *to determine socioeconomic status*, practice, employment status).

**Table 2: Planned data collection for evaluation of the process of delivery**

| **WHAT** | **HOW** | **WHO AND WHEN** |
| --- | --- | --- |
| **CONTEXT: Where we were delivering the service**  Describe practice characteristics, locality, population served for the 5(6) sites | - Produce summary description of each site: location and description of the area (urban industrial, suburban etc etc); Practice characteristics – population size, no of GPs, other services offered; population served (local public health stats for area – deprivation, inequalities etc) | - JW/PR re location and practice characteristics – start now - LC(JR) to help with local public health data |
| **REACH: Who we were delivering the service too**  Summarise who took part and how they compared with the profile of patients at the practices | - Describe the characteristics of the patients using the service: data base to include age, sex, postcode (SES), ethnicity, employment status - Collect routine data from the practice on age, sex, ever had recorded mental health history (what else) | - JW/PR collecting on existing database - LC/JR to provide categories based on evidence to date* |
| **DOSE DELIVERED:**  **Organisational context**: ethnographic observation/interviews with GPs, PM, practice team, BB team. Can we see barriers to delivery (using SEAM framework)  **Consultation model**: observation of practice vs described characteristics; interviews with case workers and patients | - Observation of service integration within the practice to look for evidence of SEAM with all stakeholders. Including informal interviews with key stakeholders to describe/ seek evidence of enablers/barriers to the stated organisational parameters   *JW/PR to collect data at end of each consultation – what type of intervention delivered in this contact*   - Observation of ~10-20 consultations to observe against each of the stated characteristics. *Observation of case workers action, not patients. Needs info leaflet for patients to explain that no data recorded about them. Consent from Jane and Peter only* | - LC as soon as *discuss with JW/PR to choose 2-3 sites. Needs written agreement from Practice Manager* - LC: between sept and dec. For analysis Jan 2015 |
| **DOSE RECEIVED**  Interviews with patients (Case studies) | - Case studies with patients following them through the ‘treatment journey’: identifying a new attendee and sitting in for all contacts, x1 contact outside of the therapeutic contact - To observe/explore impact of dose delivered on wellbeing/mental health and recognition, reciprocity, resilience - 2 or 3 at most in this depth | - LC to set up with JW/PR – at least one case with each - *Consent from patient for service evaluation interview. Update prompt list – LC* |
| **EARLY IMPACT**  Interviews | - Interviews: purposive sampling (explanatory variables as above); before and after interviews; exploring impact on recognition, reciprocity and resilience; capacity to manage demands-resources. Analysis – phenomenographic, why do people experience things differently | - JW/PR collect data, Antony on DB: ongoing until end of Feb 2015. *Anticipate n~300* - JR/LC do analysis March 2015 - LC interviews autumn 2014 (n~15) - JR/LC analysis Jan 2015 |

Planned analyses included:

*Who used the service (had access to the service)*

- Clients referred in to the service: total number, demographics, baseline scores
- Comparison of characteristics of clients completing or dropping out of the service

*Impact of care*

- Change in WEMWEBS^18^ scores from baseline to discharge from the service: with regression modelling to describe effect of age, sex, employment status, past history of MH problems, ethnicity, CISR20^20^
- Change in Meaningful Occupation^19^ scores from baseline to discharge: with regression analysis to determine the impact of age, sex, CISR20^20^

The revised timeline for the evaluation is shown in Table 3.

**Table 3: Revised timeline for Phase 2**

|  | Sep-14 | Oct-14 | Nov-14 | Dec-14 | Jan-15 | Feb-15 |
| --- | --- | --- | --- | --- | --- | --- |
| Service Delivery | Case workers | | | | | |
| Outcomes data collection (quantitative) | Case workers collect baseline and end of service data | | | | | |
| Evaluation of process of delivery (qualitative data) |  |  | LC observation data (see Table 2) | | |  |
| Evaluation of process of integration | LC observation data (using NPT framework – see Figure 5) | | |  |  |  |

**4.3 What we found**

4.3.1 *Service Integration and implementation*

Key findings under each of the NPT^15^ headings are shown in Table 4. (See also data appendix I)

Table 4: Key findings from the Phase 2 integration analysis

|  | **Learning points identified** |
| --- | --- |
| **Sense Making** | Case workers understanding of the distinct BounceBack approach continued to develop over the project time  Patients continued to arrive at appointments with limited understanding of the approach, but quickly picked up the model through using the service  GPs (those referring the patients in) had not necessarily grasped the purpose of the intervention and so were not starting sending patients with an understanding of their needs that matched the BounceBack approach  Policy makers liked the idea in principle but were still focused on other models |
| **Engagement** | Most practices engaged, and referral rates improved. Lack of clarity about how BounceBack fitted within a broader model of primary care and primary mental health care remained an obstacle (for example, competing service models being introduced during the timeframe of the project). The wider organisational context (including other priorities) seemed to be the main reason for lack of engagement of practices with the new service. DNA rates (see 4.3) were around 30% suggesting that some patients were still not all recognising themselves as candidates for the service/the service as appropriate for them |
| **Action** | Caseworkers skills continued to develop. The introduction of regular clinical supervision was recognised as important. Opportunities for resources to support patients outside of the consultation were identified (eg leaflets reinforcing the work described within the consultation). The need for skills as well as understanding of the approach to be shared across the practice team was identified – to support continuity of approach across the practice team and consistency of care for patients |
| **Monitoring** | The project was significantly under resourced (both a lack of resource and mismatch in terms of type of resource) to support this element. Case workers spent a lot of time feeding back to practice teams, but with limited opportunity to demonstrate impact and so support sense making, engagement and action. But case workers were therefore limited in their capacity to collect key outcomes data. Despite this, feedback from patients and practice staff was positive about the service. (appendix H) |

The data generally supported a view of moderately successful integration into the new practices. We noted that many practice staff and patients reported understanding and supporting the goals of the new service, and being willing to engage. However we found that this didn’t necessarily translate into action in the form of referrals and attendance. Two practices in particular (P4 and P5) did not engage despite extensive input from case workers. External factors played a part in this (for example practices were upgrading to a new version of the practice software EMIS in October 2014 – during the middle of our phase 2 delivery. A significant service change which limited capacity for engagement).

Our findings suggest that roll out of the intervention requires:

- Early and extensive input especially to patients and GPs about the nature and purpose of the service: using targeted leaflets, and videos to support understanding from the outset
- Contextual changes significantly impact on capacity to engage: any service change needs to be a whole systems driven change (integrated with external policy/performance management as well as internal practice systems)
- Case workers need training from the outset in the distinct model. Our case workers were involved in developing and refining the model (phase 1). This ‘blurring’ of roles^16^ was important in helping develop an appropriate and deliverable model of care, but potentially contributed to some loss of fidelity in delivery of the model in the phase 2 evaluation stage. GPs also need training in the approach to support continuity of approach within a whole practice team
- Robust data collection systems that can be integrated into usual practice systems, or undertaken by externally funded researchers are needed. Case workers struggled to collect the service evaluation data needed for the project, as well as deliver the service.

4.3.2 *Process of Service Delivery*

In keeping with good practice in clinical research studies, our database collecting service use, baseline and follow up data was held by a project administrator at AIW, not by the evaluation team. We planned a data analysis of the completed data set at the end of February 2015.

Unfortunately on reaching the end of data collection, it became apparent that we had a number of problems with the data collection and collation process. It was only at the end of the project that we identified the problems with an incomplete data set. We present below the data that we had available to us in the evaluation period.

4.3.2.1 Who received care

Table 5: Total number of patients referred in to the service, n=247

| **Practice reference number** | **Number of patients referred** | **Number of patients DNA’d** | **Number (%) of patients attending 1^st^ appointment** |
| --- | --- | --- | --- |
| P1 | 78 | 10 | 68 (87%) |
| P2 | 22 | 8 | 14 (64%) |
| P3 | 106 | 31 | 75 (71%) |
| P4 | 1 | 0 | 1 (100%) |
| P5 | 2 | 1 | 1 (50%) |
| P6 | 27 | 11 | 16 (59%) |
| P7 | 11 | 2 | 9 (82%) |
| TOTAL | 267 | 63 | 184 (69%) |

Table 6: Demographics of patients who attended /dna’d (incomplete data sets)

|  |  | Attended 1^st^ appointment | Dna’d 1^st^ appointment |
| --- | --- | --- | --- |
| SEX* | Male (n= 80) | 62 (40% of attendees) | 18 (36% of dnas) |
|  | Female (n= 125) | 93 (60% of attendees) | 32 (64% of dnas) |
| AGE (years)** | <20 | 2 | 2 |
|  | 20-29 | 17 (24.6% of attendees) | 10 (35.7% of dna’s) |
|  | 30-39 | 17 (24.6%) | 10 (35.7%) |
|  | 40-49 | 12 | 5 |
|  | 50-59 | 17 (24.6%) | 1 (3.5%) |
|  | 60- | 4 | 0 |
|  | TOTAL | 69* | 28* |

* data from all practices up to December 2014 (incomplete data set)

** data from P3 only up to December 2014 (incomplete data set)

From our service use data, we noted

- High first appointment attendance rates (69%). Supporting our qualitative data in suggesting that people understood and were engaging with the service.
- Characteristics of people we were seeing: an expected higher % of women than men used the service, but we also noted that a high proportion of our service users were men. Suggesting that both men and women were experiencing mental health need problems, and that men also recognised our service as appropriate for them.
- Service users from across a full age range, evenly distributed, were noted to use **
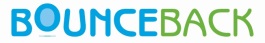
**
- Patients who DNA’d their first appointment had similar gender characteristics to those who attended, supporting our interpretation that the service was recognised as appropriate by both sexes. We noted a possible reduction in DNA’s amongst older patients, but this could simply be an artefact of small numbers.

Baseline scores for outcome measures

For the reasons highlighted, we had a very limited data set available for patients seen. However in the 44 (out of 184) patients where we had baseline scores (Table 7) our data showed:

**Table 7: Baseline scores for 44 participants**

| **ID** | **WEWEBS** | **MAPA** | **FATIGUE** | **ID** | **WEWEBS** | **MAPA** | **FATIGUE** | **ID** | **WEWEBS** | **MAPA** | **FATIGUE** |
| --- | --- | --- | --- | --- | --- | --- | --- | --- | --- | --- | --- |
| #1 | 24 | 108 | 58 | #16 | 31 | 126 | 61 | #31 | 40 | 90 | 63 |
| #2 | 31 | 56 | 24 | #17 | 21 | 93 | 53 | #32 | 42 | 82 | 68 |
| #3 | 31 | 63 | 55 | #18 | 26 | 65 | 58 | #33 | 26 | 77 | 60 |
| #4 | 48 | 90 | 58 | #19 | 25 | 67 | 55 | #34 | 20 | 55 | 82 |
| #5 | 21 | 88 | 39 | #20 | 39 | 90 | 41 | #35 | 29 | 80 | 45 |
| #6 | 39 | 90 | 41 | #21 | 29 | 73 | 55 | #36 | 32 | 63 | 47 |
| #7 | 19 | 51 | 54 | #22 | 19 | 107 | 61 | #37 | 34 | 100 | 58 |
| #8 | 42 | 88 | 63 | #23 | 22 | 57 | 44 | #38 | 26 | 101 | 62 |
| #9 | 23 | 95 | 61 | #24 | 37 | 91 | 53 | #39 | 30 | 79 | 58 |
| #10 | 25 | 87 | 63 | #25 | 44 | 87 | 64 | #40 | 51 | 77 | 69 |
| #11 | 31 | 50 | 54 | #26 | 26 | 72 | 56 | #41 | 50 | 119 | 60 |
| #12 | 28 | 86 | 57 | #27 | 42 | 82 | 54 | #42 | 44 | 109 | 59 |
| #13 | 41 | 84 | 69 | #28 | 14 | 117 | 52 | #43 | 31 | 88 | 56 |
| #14 | 38 | 122 | 66 | #29 | 25 | 62 | 56 | #44 | 28 | 76 | 59 |
| #15 | 24 | 120 | 53 | #30 | 27 | 55 | 58 | **MEAN** | **10.6** | **29.0** | **18.5** |

From Table 7, we note that:

- Mean baseline WEMWEBS score was 31.5
  - The UK mean score from the Health Survey for England 2012 was 51.^21^ Suggesting that our service uses had lower mental health and wellbeing scores than the general population.
- Mean baseline MAPA score 84.5
  - In a US validation study, the mean score in a general population was 214, suggesting that our service users have reduced engagement with meaningful activity
- Mean CISR20 score 56.4
  - Malekzedah defined a non-fatigued population as those with a score of <34.^20^ On this scoring, only one of our patients was identified as non-fatigued
  - In the same study of patients with multiple sclerosis, those who were identified as being fatigued had a mean CISR20 fatigue score of 46.2 (SD 5.0). Our baseline mean score was 56.4. Suggesting that participants in our project had fatigue levels on a par with those suffering from a significant physical illness.

4.3.2 What service we delivered

The average no of appointments per patient = 2.6

*Dose delivered by case workers*

Analysis considered to what extent case workers were delivering the **
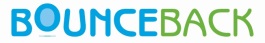
**model of care (Box 3) consisting of: use of a biographical lens, an unstructured (exploratory) consultation, exploring the (im)balance of resources and demands, identifying opportunities for modification.

Analysis of case observation data (appendix I ) suggests that:

- We were partially delivering the described **
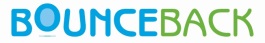
**intervention: case workers were good at using a biographical lens, and often collected broad data to inform their interpretation of need. Case workers often explored (im)balance of resources and demands. Although supporting the patient in identifying opportunities for change was less consistent
- But caseworkers struggled when the patient brought/started with a strong medical agenda (highlighting the need for additional training of caseworkers and/or a need to address patients expectations too – possibly an element of patient education, possibly also to include changing the conversation/narrative started with the GP)
- Caseworkers were also observed to struggle to go beyond trying to ‘fix’ problems for their clients – the AIW model of practical problem solving. To also see their role as helping patients understand their problems differently. This improved over time as case workers gained understanding of the **
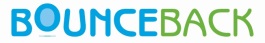
**model, and indeed the project team refined our description of the intervention. The observations highlight the importance of having a clear vision of what the intervention is, and possibly a need for further training of caseworkers in the skills needed to adopt a more exploratory approach^9^ to consulting with patients.
- LC’s role was envisaged as an objective observer of delivery of the intervention. In light of her skills and experience in mental health care, the observer role became ‘blurred’^16^ as she was also able to reflect back to the case workers on how they could do things differently. LC thus became involved in shaping the delivery of the intervention, not just as a neutral observer. Whilst this enhanced the development of the complex intervention, it has implications for the nature of the evaluation. In subsequent work, we would recommend having academic roles supporting development and integration, but with separate roles for process and outcome evaluation.

What service did the patient perceive they received

Based on informal mini interviews, observation, and case study interviews with patients (conducted by LC) (see APPENDIX H, I), patients and practitioners described receiving the essential elements of the Bounce Back approach and experiencing positive effects from the service process

- HOW THE SERVICE HELPED: by providing support, helping them make sense of things, being prompt and accessible, helping people develop strategies for doing things differently, offering practical solutions
- THE IMPACT: was in helping people make progress, feel better

The qualitative data demonstrated that we had addressed our goal to help people understand their distress differently, work collaboratively to identify barriers/difficulties in daily living, and so support them to overcome the difficulties.

4.3.3 *Impact of Care*

Case study data in Appendix I demonstrates that patients recognised the service as relevant for them and themselves for the service; were able to work with the service (reciprocity) to manage their mental health. There was limited, but early suggestion of potential development of resilience

On outcome measures

We had complete data set for only 11 (6% of our patients who attended a first appointment) of our participants. We present the full data set in the table below, with only some very broad brush comments on our observations.

Table 8: Showing baseline and end of project scores for n=11 (6%) of our service users

| **Patient id** | **WEMWEBS Baseline** | **WEMWEBS**  **End** | **Change in wemwebs** | **MAPA Baseline** | **MAPA End** | | **Change in MAPA** | | **FATIGUE Baseline** | **FATIGUE End** | **Change in fatigue** |
| --- | --- | --- | --- | --- | --- | --- | --- | --- | --- | --- | --- |
| #34 | 20 | 33 | +13 | 55 | 100 | | +45 | | 82 | 55 | **-17** |
| #35 | 29 | 48 | +17 | 80 | 106 | | +26 | | 45 | 68 | **+23** |
| #36 | 32 | 29 | -3 | 63 | 87 | | +25 | | 47 | 53 | **+6** |
| #37 | 34 | 50 | +16 | 100 | 119 | | +19 | | 58 | 50 | **-8** |
| #38 | 26 | 47 | +21 | 101 | 108 | | +7 | | 62 | 51 | **-11** |
| #39 | 30 | 40 | +10 | 79 | 79 | | 0 | | 58 | 57 | **-1** |
| #40 | 51 | 63 | +12 | 77 | 100 | +23 | | 69 | | 72 | **+3** |
| #41 | 50 | 54 | +4 | 119 | 122 | +3 | | 60 | | 64 | **+4** |
| #42 | 44 | 50 | +6 | 109 | 120 | +11 | | 59 | | 67 | **+8** |
| #43 | 31 | 40 | +9 | 88 | 91 | +3 | | 56 | | 61 | **+5** |
| #44 | 28 | 46 | +18 | 76 | 91 | +15 | | 59 | | 66 | **+7** |
|  | Mean change in wemwebs | | +11.2 | Mean change MAPA | | +16.1 | | Mean change in fatigue | | | **+4.8** |

- A trend of an increase in WEMWEBS score suggests a positive improvement in mental health
- A trend of an increase in MAPA score suggests an increase in time spent on Meaningful Activities (although overall scores are still low compared with the elderly population that was validated on;^19^ where mean score was 214 ± 83 (a sample of well adults aged 65+ recruited from supported care home setting)
- A trend of an increase in fatigue scores – suggesting greater fatigue

**4.4 Summary: what we learnt**

Despite the limitations of our outcome measurement, overall our project demonstrated the development of a new complex intervention (a ‘flipped model’ of mental health care employing a Socio-Psycho-Bio model), which we were able to partially integrate into practice; with data providing evidence that we had successfully offered the model of care to patients with some positive impact on their recognition of the service as being useful, their capacity to engage with this way of working (reciprocity), and possibly with developing resilience. We have early signals that we have a new intervention, associated with positive outcomes that may be attributed at least in part to the intervention.

Stufflebeam argues that we need to go beyond viewing evaluation as synonymous with assessment of performance.^22^ Rather that an evaluation study should assist an audience to ‘assess an object’s merits and worth’. Our findings support a conclusion that the new complex intervention that is BounceBack potentially offers value. Our work supports a conclusion that the translational research should continue to the next stage of formal impact and economic evaluation.

- 1. **Implementing our learning - refining the intervention and the evaluation model**
     1. **The Intervention: revising our model of** **
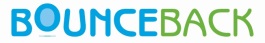
**

Our findings highlighted the importance of a clear description and a shared understanding of the care model amongst all members of the patient-practice team, in order to facilitate appropriate engagement (access), actions and monitoring. We have therefore described a new Socio-Psycho-Bio (SPB)model of primary mental health care (Figure 6) – a contrast to the traditional BioPsychoSocial model. Which first understands and addresses the (im)balance of wider demands and resources on experiences of distress before seeking psychological or medical explanation for experience. A core goal for the service is to support patients in making sense of their distress using the SPB model.


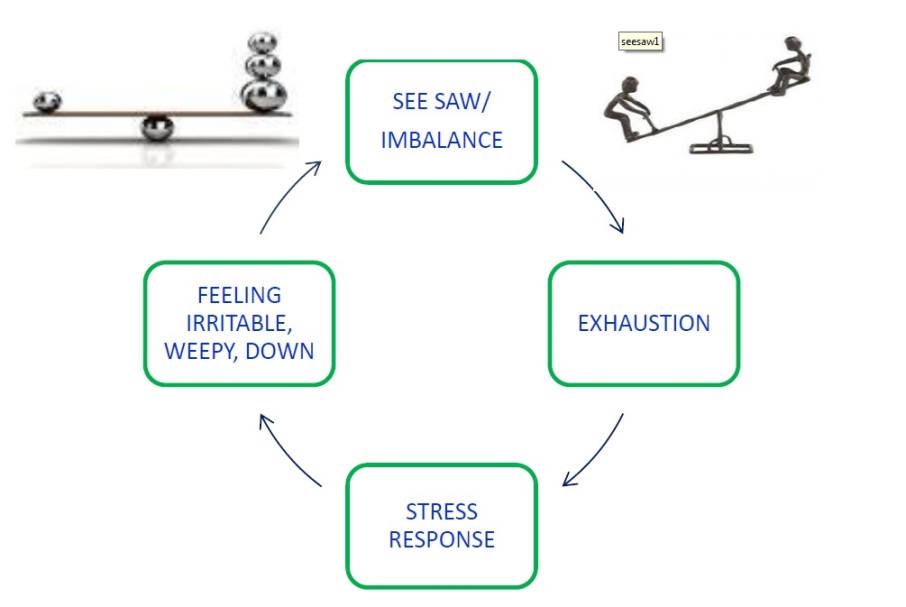
**Figure 6: The
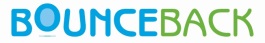
model: a SocioPsychoBio (SPB) model of distress**

We also recognise the importance of continuity of approach across the team to facilitate appropriate and timely referral in to the service, and so promote patient engagement with the SPB approach. Patients recognition of the suitability of the service for them is shaped by how the wider health team initially help them make sense of their mental health needs. It is therefore key that the whole practice team understand the model in order to support recognition and reciprocity – the building blocks for resilience.

We originally described using an unstructured consultation approach – to contrast our model with the structured model used for example in current Integrated Access to Psychological Therapy (IAPT) services. (Local IAPT services require patients to complete multiple mental health scores at their initial assessment). Observation of practice described instead that case workers were involved in an exploratory model of practice, collecting multiple data sources used to make sense of and interpret a patient’s distress. The consultation approach uses data describing patient and practitioner experiences and perceptions as well as mental health concepts and theories to construct a new individualised narrative of distress. This approach maps to the model of interpretive practice described by Reeve^23^ and is an essential component in the generalist model of practice.^24^ The **
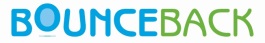
**approach thus describes (articulates) a generalist (interpretive) approach to understanding individual health care needs.

Based on these observations, we have further refined the **
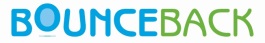
**care model – see Box 5

| **Box 5: The 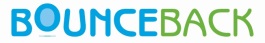care model version 3 – BB3** | |
| --- | --- |
| **The core components – the consultation**   - Informed by a SPB model of distress (see Figure 6) - Using an exploratory consultation approach, collecting multiple data sources to patient and practitioner make sense of distress using the SPB model - Used to identify potentially modifiable factors - Trial and review to refine individually tailored story of distress | **The variable components – the service model**  S: use targeted resources to ensure all parties understand the approach  E: Direct and flexible access/referral  Train GPs in the SPB model (to support Sense Making and improve referral (E)  A: trained case workers to deliver care  -Ensure ongoing supervision for case workers to maintain and develop skills  - signposting to resources that support resilience (accessible by the whole practice team)  M: Feedback process and impact of care  Record process and progress of care in patient notes to support continuity of approach across the primary care team |

- - 1. The impact of the **
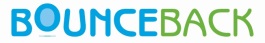
**service

**
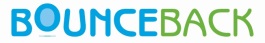
**care was delivered to 184 patients , 60% female. Data demonstrated that we successfully delivered the care model, with some perceived impact on recognition and reciprocity, contributing to an early signal on enhanced resilience. Our quantitative data was insufficient to draw meaningful conclusions but did hint at an improvement in wellbeing andmeaningful occupation, albeit with no impact on fatigue

- - 1. The process of evaluating the **
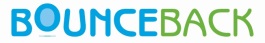
**service

Integrating the service and evaluation teams (the ‘blurring model’ described by Evans^16^) was supportive of, indeed necessary to, develop and refine the complex intervention (To unlock the red traffic lights of phase 1). The use of this approach to develop a successful intervention is supported by our early evaluation findings from phase 2.

However our integrated approach, and the general level of resource available for evaluation across the whole project, was insufficient to support the quantitative data collection needed for the originally planned evaluation in Phase 2. We propose that in future work, the integration stage should be undertaken using the blurred model we describe. However there should also be an additional researcher role dedicated for data collection – particularly outcome data collection. We also recognised a lack of administrative support for the formal evaluation of the project (capacity that was not available within the AIW administrative team). Stronger outcome data in future evaluation of innovation products would need a larger focus of resource on the evaluation side than was possible in our project.

The changes to the project meant we were unable to undertake the planned economic evaluation but believe this would be more appropriate in a pilot study

- 1. **Conclusions**

Despite these limitations, we have successfully:

1. Completed the first two stages in the development of a complex intervention (as described by the MRC framework^25^), having developed an intervention (from theory and practice) and shown that it is feasible to deliver
2. Demonstrated a need and desire for the service

We are ready to undertake the next steps in implementing our innovation: a pilot study to provide preliminary data needed to scale and cost a full randomised clinical trial.

1. **DISSEMINATION**

This report makes clear that our project changed significantly during the 2 years. We therefore also needed to change our dissemination strategy. Recognising the action research elements in our development and initial testing of the complex intervention that is the **
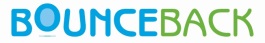
**model of care, our dissemination strategy was revised to a stronger emphasis on shared reflection and learning.

**5.1a Shared reflections on the process of change**

We presented a critical review of our Phase 1 work at two meetings over the course of the project:

- The Society for Academic Primary Care annual Conference 2014 (Edinburgh, oral presentation)
- Manchester University Department of Primary Care (invited speakers, Departmental seminar)

Both presentations focused on the process of developing and integrating a new complex intervention, including the development of a methodology of Translational Scholarship as a tool to support change. Slides from the meetings are shown in APPENDIX J. The work was well received . We are continuing academic conversations with partners about developing novel methodological approaches to support innovation in practice through the development of practice-based evidence.^26,27^

**5.1b Shared learning**

As highlighted in Box 5, a finding from our evaluation was the need to describe and share our new model of care with the whole service team - notably practitioners

- who were potentially referring in to **
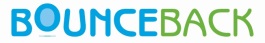
**(to maximise appropriate referrals and so efficacy of the service)
- who were providing ongoing support for patients (supporting continuity of approach and so optimising impact of the intervention)

We therefore ran a GP Education event for 60 GPs in conjunction with the local Family Doctors Association: as an opportunity to share our learning, support local GPs in understanding and engaging with the approach, and gain further feedback on the model of care we have developed.

APPENDIX J gives the timetable for the event, and the follow up learning points circulated to the GPs

GPs reported to us that they found the event interesting and helpful, but also that

- the **
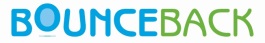
**model helped them think differently about mental health consultations (and referrals)
- the **
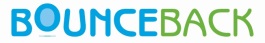
**approach gave them practical tips to consult differently
- the **
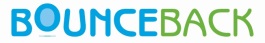
**model was relevant to the needs of patients in their daily practice and offered a useful alternative to the more biomedical focused model (a way to avoid overmedicalisation)
- hey wished they could have the **
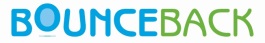
**service in their own practices

The feedback from the event resonates with and reinforces the evaluation data described in section 4. More details of the feedback are shown in APPENDIX L.

**5.2 Further planned dissemination**

We are currently preparing two papers for publication

- On the process of Complex Interventions development (phase 1)
- Describing the new socio-psych-bio model

We will also be preparing a bid for Research for Patient Benefit funding to run a pilot trial of the **
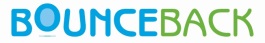
** service in new practices, in order to test and refine outcome measures and generate the data needed to support a full Randomised Controlled Trial proposal.

1. **OVERALL REFLECTIONS**

**6.1 Summary of outputs**

- a new Complex Intervention: the SPB model of mental health (Figure 6) and the **
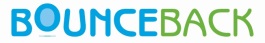
**model of delivery (Box 5)
- a described methodology for translational research using NPT
- demonstrable impact of a service on a local community (patients, practices and GPs - via the GP education event)

**6.2 Strengths and limitations of our work**

6.2.1 *Strengths*

- We have developed/described and feasibility tested a new complex intervention, ready for pilot and then full trial
- Through a translational research approach to generate practice based evidence^26^ that produces a model of care that fits within the practice context
- We have supported significant developments in the work and working of a local charity. AiW Health will continue to benefit from the educational tools devised by the **
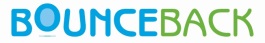
** project. Including in raising awareness of the generalist, interpretive approach – which can be applied also in other areas of work within the charity. Caseworkers have also developed better understanding of the processes and procedures involved in the development and delivery of a new way of working in clinical practice, and in supporting innovation and change. This will impact on the organisation and ensure that AiW Health continues to develop by exploring further research opportunities. AiW Health has also identified a need to continue to work closely with Primary Care Teams and throughout our local community in order to ensure best outcomes for individuals; for example – we have identified that there is a lack of knowledge of local communities/resources and this may contribute to inequity of access to services. AiW Health will explore opportunities to maintain our well established links with Wirral group practices.

6.2.2 *Limitations*

- This was a service development project and so informs research development, but doesn’t directly generate evidence of impact.
- As highlighted, outcomes evaluation work was underfunded/understrength to deliver some of the hoped for data.

**6.3 Next steps**

The **
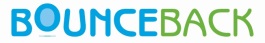
**project has successfully developed, described and feasibility tested an innovative new model of mental health needs assessment for use in primary care. The project has also provided valuable new understanding of the approach of translational scholarship – collaborative generation and implementation of evidence and learning through partnership between the charitable and academic sectors. The work will now directly inform further research and service development work which will support translation of local impact into wider, national impact

**Author details and contacts**

Report prepared by: Joanne Reeve, Lucy Cooper, Jane Watkins, Peter Rosbottom

Corresponding Author:

Dr Joanne Reeve

Warwick Medical School. University of Warwick, Coventry CV4 7AL

j.reeve.1:@warwick.ac.uk

**References**

1. World Health Organisation 2011. Global status report on non-communicable diseases 2010. Available at www.who.int/nmh/publications/ncd_report_full_en.pdf
2. Department of Health 2011. No health without mental health: a cross-government mental health outcomes strategy for people of all ages – a call to action. Available at www.gov.uk/government/uploads/system/uploads/attachment_data/file/213760/dh_123990.pdf
3. Chan WS, Whitford DL, Conroy R, Gibney D, Hollywood B. 2011. A multidisciplinary primary care team consultation in a socio-economically deprived community: an exploratory randomised controlled trial. BMC Health Serv Res; 11: 15.
4. The AMP project. www.amproject.org.uk/
5. Kovandžić M, Chew-Graham C, Reeve J, Edwards S, Peters S, Edge D, Aseem S, Gask L, Dowrick C. 2011. Access to primary mental healthcare for hard-to-reach groups: from 'silent suffering' to 'making it work'. Social Science and Medicine; 72(5): 763-772.
6. http://www.mentalhealth.org.uk/content/assets/pdf/publications/starting-today-background-paper-3.pdf
7. www.aiw.org.uk/about-us-2/
8. Reeve J, Lloyd-Williams M, Payne S, Dowrick CF. 2009. Towards a re-conceptualisation of the management of distress in palliative care patients: the Self-Integrity Model. Progress in Palliative Care; 17(2): 51-60.
9. Reeve J. 2010. Interpretive Medicine: supporting generalism in a changing primary care world. London: Royal College of General Practitioners Occasional Paper Series, 88.
10. www.hra.nhs.uk/research-community/before-you-apply/determine-whether-your-study-is-research/
11. Research Governance Framework for Health and Social Care (2005) Available at www.gov.uk/government/uploads/system/uploads/attachment_data/file/139565/dh_4122427.pdf
12. Medical Research Council (MRC) Developing and evaluating Complex Interventions. 2008. www.mrc.ac.uk/documents/pdf/complex-interventions-guidance/
13. Normalisation Process Theory. www.normalizationprocess.org/ NPT framework website
14. Reeve J, Blakeman T, Freeman GK, Green LA, James P, Lucassen P, Martin CM, Sturmberg JP, van Weel C. 2013. Generalist solutions to complex problems: generating practice-based evidence - the example of managing multi-morbidity. BMC Family Practice; 14:112. DOI: 10.1186/1471-2296-14-112.
15. NPT Toolkit http://www.normalizationprocess.org/npt-toolkit.aspx
16. Evans S, Scarborough H. 2014. Supporting knowledge translation through collaborative translational research initiatives: ‘bridging’ versus ‘blurring’ boundary-spanning approaches in the UK CLAHRC initiative. Social Science and Medicine 2014, 106, 119-127
17. Yin RK. Case study research. Design and methods. London, Sage Publications ,2003
18. Warwick-Edinburgh Mental Well Being Scale (WEMWEBS) http://www2.warwick.ac.uk/fac/med/research/platform/wemwbs/
19. Eakman AM, Carlson ME, Clark FA. 2010. The meaningful activity participation assessment a measurement of engagement in personally valued activities. Int J Aging Hum Dev 70(4): 299-317.
20. Malekzadeh A, Van de Geer-Peeters W, De Groot V, Tenuissen CE, Beckerman H, TREFAMS-ACE study group. 2014. Fatigue in patients with multiple sclerosis: is it related to pro- and anti-inflammatory cytokines? Disease Markers. http://dx.doi.org/10.1155/2015/758314
21. www2.warwick.ac.uk/fac/med/research/platform/wemwbs/researchers/interpretations/wemwbs_population_norms_in_health_survey_for_england_data_2011.pdf
22. Stufflebeam DL. 2001. Evaluation Models. New Directions for Evaluation; 89: 7-98.
23. Reeve J. 2010. Interpretive Medicine: supporting generalism in a changing primary care world. London: Royal College of General Practitioners Occasional Paper Series, 88.
24. Reeve J. Supporting Expert Generalist Practice: the SAGE consultation model. British Journal of General Practice 2015; 35:207-208
25. MRC Complex Interventions www.sphsu.mrc.ac.uk/research-programmes/ev/methop/evalci.html
26. Green LW. 2009 Making research relevant: if it is an evidence-based practice, where’s the practice based evidence? Family Practice; 25: i20-i24.
27. Reeve J, Blakeman T, Freeman GK, Green LA, James P, Lucassen P, Martin CM, Sturmberg JP, van Weel C. 2013. Generalist solutions to complex problems: generating practice-based evidence - the example of managing multi-morbidity. BMC Family Practice; 14:112. DOI: 10.1186/1471-2296-14-112.

**Appendices**

| 1. **Original Bid** | Page 36 |
| --- | --- |
| 1. **Phase 1 data** | Page 40 |
| 1. **Project materials produced** | Page 46 |
| 1. **Phase 1 outcomes summary** | Page 51 |
| 1. **Manual describing service delivery** | Page 54 |
| 1. **Data collection tools** | Page 59 |
| 1. **Contextual information for case analysis** | Page 67 |
| 1. **Service feedback** | Page 68 |
| 1. **Impact case studies (Phase 2)** | Page 71 |
| 1. **Slides from presentations** | Page 73 |
| 1. **Information to delegates GP event** | Page 75 |
| 1. **Feedback from delegates** | Page 80 |

**List of Tables and Figures**

| **Figure/Table/Box** | | **Page** |
| --- | --- | --- |
| Box 1 | Understanding Access | 4 |
| Box 2 | The original Bounceback intervention (BB1) | 6 |
| Figure 1 | Original timelines for the BounceBack project | 7 |
| Box 3 | Describing the 4 Key concepts in NPT | 9 |
| Figure 2 | Traffic light framework template | 12 |
| Figure 3 | Progress March-July 2013 | 13 |
| Figure 4 | Progress 2014 – and back in to the green | 13 |
| Table 1 | Examples of actions arising from the traffic light review | 14 |
| Box 4 | Revised Bounceback Intervention BB2 | 16 |
| Figure 5 | Evaluation strategy overview | 17 |
| Table 2 | Planned data collection for process delivery evaluation | 19 |
| Table 3 | Revised timeline for phase 2 | 21 |
| Table 4 | Key findings from the phase 2 integration analysis | 21 |
| Table 5 | Total number of patients referred in to the service | 23 |
| Table 6 | Demographics of patients who attended/dna’d | 23 |
| Table 7 | Baseline scores | 24 |
| Table 8 | Showing baseline and end of project scores | 26 |
| Figure 6 | The Bounceback model: a SocioPsychoBio model of distress | 28 |
| Box 5 | The Bounceback care model version 3: BB3 | 29 |

**Appendix A: The bid to NIHR IESDF**

***A1. Summary***

This project aims to address the growing burden of mental health problems within the community through developing innovative approaches to access to care in the primary care setting.

A recent NIHR funded programme grant (0606/1071) provides evidence that issues of access to services contribute to inequalities and poor service provision. Importantly, access issues are related to the *nature* and not just the availability of care.

Primary care is the main point of access to formal health care for most people; being the setting in which needs are assessed and, where appropriate, acts as a gatekeeper for access to further care. Current models of care assess clinical levels of mental distress which may involve referral to support services, including 3^rd^ sector organisations, for help with practical problems that can limit recovery (HM Government 2011).

Our project differs from existing models of care through demedicalising the mental health needs assessment process. We recognise that nonmedical, practical problems impact not only on mental health recovery (HM Government 2011), but also on creation of need. The Advocacy in Wirral (AiW) model recognises that practical issues are often the primary issue in many people’s mental health problems; viewing clinical assessment/pathways of care as a follow-on for when health problems persist despite practical problems having been addressed. We have had demonstrable impact on individual people’s health and wellbeing in 20 years of delivering our service, particularly in communities we know have difficulty accessing other services. These include groups with complex mental health issues (Attention Deficit Hyperactivity Disorder, ADHD; personality disorder; and medically unexplained symptoms); groups with comorbidity; and vulnerable groups from socioeconomically deprived communities, including the unemployed.

We now seek to address concerns related to wider inequity within these communities through integrating our service into the primary care setting: to support innovation in the access to care in terms of how needs are assessed. This involves 2 elements – influencing how GPs discuss mental health issues with patients presenting to them; and providing ready access to the non-medical needs assessment process that is the AiW model.

This innovation project will deliver on 3 aims:

1. to *integrate* the AiW model into a primary care (general practice) setting – being the context in which we know our 3 target groups often seek help (as above);
2. to *apply* the model and deliver care to 500 clients over a 12 month period, supported by an integrated evaluation run on action-learning principles to identify learning and benefit for patients, primary care teams, health services, and communities;
3. to *inspire* wider change in service design and provision through disseminating learning through conversations with commissioners, education programmes, community groups.

The project will offer immediate benefit to local service users and primary care teams. But most importantly, the learning from the project will benefit communities (addressing inequalities in mental health); health services (improved targeting of patients for services); and policy makers/commissioners (deciding future shape of services).

***A2. How we will achieve this:***

1. **By Improving access to integrated care**

We will improve access to appropriate, quality care through improving the route of access to non-medicalised needs assessment for people experiencing distress. By providing a route to available care:

1. Embedding AiW case workers within primary health care practices/teams: with local champions and advertising used to raise local awareness of the service, including self-referral options;
2. GPs in participating practices offered an educational support package to encourage safe and appropriate referral to the AIW case worker for all patients where exhaustion or resilience issues are identified as playing a significant part in their mental distress (see Reeve et al 2009) [i and ii particularly target complex mental health and comorbidity groups];
3. Vulnerable groups from deprived communities eg the unemployed may not already be accessing general practice services. They will be targeted through outreach with local partners from the Department for Work and Pensions (DWP), back to work programmes, and other community services identified through partnership working with Liverpool Clinical Commissioning Group (CCG) primary mental health care pathway.

By providing quality de-medicalised assessment of mental health needs and delivery of care:

1. First appointment with an experienced AiW generalist Needs Assessor/case worker for holistic assessment of need focusing on practical problems which limit (mental) health as a resource for living;
2. Formulation of action plan: including referral for specialist support (eg debt counselling) via existing AiW networks; ongoing support with case worker; agreed action points for the individual. With clear emphasis on educational/empowerment – helping the client but also enabling them to learn how they could do it next time (building resilience);
3. Follow up until practical problems limiting daily living & engagement with meaningful occupation addressed (*experience suggests average contact = 3-4 visits;can be 10-12 for larger debt problems);*
4. Resilience/Forward Planning meeting: once immediate issues resolved, to consolidate ‘learning’ (dealing with future problems), action plan for ‘maintenance ‘, info about support groups, and how to contact us again if you need.

At any stage if the patient or worker identifies other needs not being addressed by the service, the patient will be referred back to their GP. Experienced AiW staff are trained in recognising red flags that need urgent referral back to clinical care.

Anticipated outcomes from this stage will include - improved mental health, improved resilience (capacity to deal with situations in future), reduced exhaustion scores and reduced use of GP/medical care.

1. **By evaluating outcomes**

We will measure

1. Baseline (0-4months) - Baseline data collection from participating practices including mental health consultation rates, antidepressant prescribing, secondary care and IAPT service (Improving Access to Psychological Therapy) referral rates;
2. Delivery (5-17months) - Individual patient outcome data collected: before and after individual scores for depression (PHQ-9), health-related quality of life (SF-12v2), resilience (Resilience Scale for Adults), exhaustion (CIS20R); consultation rates in primary care and referrals for additional care; engagement in meaningful occupation; service satisfaction (general practice assessment questionnaire instrument); and prescribing data (number and type of medication);
3. Analysis (18-24months) - data analysis to derive change in individual scores on depression, resilience, exhaustion, service use (including medication and referral), service satisfaction. Along with practice level impact (changes from baseline in consultation rates, prescribing and referrals).

We estimated seeing 500 service users per annum (based on past experience of AiW service demand, service delivery and capacity).

***Our project Milestones***

**0-4months: Integration (embed the project).** Educate and integrate with PC teams (6 practices, covered by 2 case workers) through training sessions and meetings within practices as well as with wider GP community (GP Forum, see [www.primarycarehub.org.uk](http://www.primarycarehub.org.uk)). Develop wider community links (via Liverpool PC mental health network), job centres etc to “communicate the availability of acceptable services to the community” (Kovandzic 2011). Set up and pilot evaluation tools: both process and outcomes.

**5-17months: Application (demonstrate impact of the intervention).** Deliver service outlined in 2.3. Evaluation as outlined in 3.4

**18-24months: Inspiration (disseminate learning).** Including negotiate service continuation with local commissioners. Host stakeholder conference to share findings and explore how integration into their own context. Reports and publications including social media. Develop educational outreach including resource for commissioners in how to start service in their area; educational programmes for GPs (through GP Forum, CPCD and Royal College of General Practitioners).

***A3. Risks and Mitigation Strategy***

Risk: Capacity – service is overwhelmed. Mitigation – pilot restricted to 6 practices. Extensive experience at AiW in service delivery and how to accommodate some increase in demand (eg though using volunteer support in AiW setting);

Risk: Dealing with ‘red flags’ – acute severe mental health need placing patient or others at risk. Mitigation - trained experienced AiW staff with protocols already in place to deal with this. Service will be integrated into primary care;

Risk: changing external context (new commissioning structures, economic downturn creating additional need, reduced resource). Mitigation - AiW and primary care working in this context for a long time – integration of the two will strengthen capacity to deal with variability and change, not diminish it;

Risk: Support of workers, especially with move to Liverpool (away from Wirral based AiW hub). Mitigation – longstanding experience at AiW of supporting workers in dealing with complex and vulnerable populations through outreach, training. Will introduce an AiW team leader as mentor /supervisor to the 2 case workers.

***A4. Timescales for evaluation***

0-4 months: action learning evaluation of the process of embedding; baseline data collection for practices.

5-17months: baseline, end of treatment and 6-12 month follow up of individual patients to assess scores as described.

Follow up assessment of practice level data (service use, referrals etc). Description of process and experience of care for individual case studies.

18-24months: analysis of data, including economic modelling of predicted costs and savings based on costs of service delivery and identified outcomes.

The evaluation will be led by the partner organisation, the Centre for Primary Care Development (CPCD, lead – Joanne Reeve). JR will be responsible for evaluation of the ‘integration’ phase. The project will employ a full-time postdoctoral researcher to lead the field work and analysis during the 12 month implementation stage. AiW staff and volunteers will also actively be involved in both the data collection and analysis stages as per our participatory evaluation approach. CPCD is part of the department of Health Services Research at Liverpool University – which has extensive experience of evaluation of health projects. JR is an NIHR Clinician Scientist in Primary Care with dual clinical training in both primary care and public health. She has extensive experience in the area of mental health research and has previously been directly responsible for the evaluation of a community health intervention project (see Reeve & Peerbhoy

***A5. Dissemination Strategy***

1. Negotiation with local commissioners for continuation of the local service (building on existing links, keeping commissioners informed throughout the project, and discussing final findings);
2. Hold a stakeholder conference/workshop to discuss innovation in needs assessment with national stakeholders: at which we will share project findings (process and outcomes) and explore how stakeholders can integrate findings into their own context;
3. Outreach: development of educational packages. For potential future service providers (outlining how to develop, deliver and integrate the service; disseminated through known contacts, websites, direct awareness raising to local and national mental health groups). And for GPs (on how to assess mental health needs using a non biomedical approach; disseminated through CPCD/GP Forum, local and hence national offices of the Royal College of General Practitioners);
4. Reports and publications disseminated through clinical and academic journals; on AiW and CPCD websites; through social media; press release.

**APPENDIX B: PHASE 1 INTEGRATION**

**Supporting data for work under the 4 NPT headings**

**B1: SENSE MAKING**

OUR GOAL: to ensure that all stakeholders knew about the **
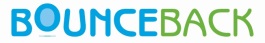
** project and understood

- The aim of the **
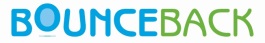
** project
- How **
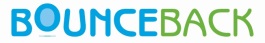
** is different from other services in primary care
- Why **
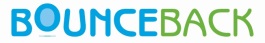
** mattered
- How they could get involved

**What we did**

*Establishing our service & project within the community*

In the local community, caseworkers distributed project information within local amenities such as libraries, post offices, church groups, pharmacies and jobcentres

In a practice setting, caseworkers attended team meetings to introduce the service. Where practices signed up to the project, repeat visits ensured that all staff (notably reception) members were provided with adequate information about the service and how to access it. Caseworkers liaised with patient user groups within group practices and attended meetings and contributed to Newsletters and practice websites.

Caseworkers arranged meetings (and in some cases cold-called) community and church groups; e.g. Beechwood Community Centre as this has an outreach network which serves Fender Way Health Centre area.

They visited local third sector groups such as Women’s Enterprise Breakthrough, Tomorrow’s Women and Wirral Change – all groups which provide support to those affected by poverty, unemployment, domestic abuse and issues which are likely to contribute to anxiety and exhaustion.

*Raising community awareness & liasing with stakeholders*

Wirral CCG communications team contacted AiW Health to offer their services in helping to promote services and publicise information across Wirral. Caseworkers arranged a meeting with the team to identify the **
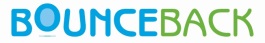
** project’s needs/target audience and to discuss the methods available for our use (there was no charge for this service.) This was a very prompt and helpful service which facilitated the promotion of **
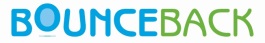
** events to Primary Care Teams and this is a resource which we can continue to use for dissemination of materials.

We attended Wirral Mental Health Forum in order to promote **
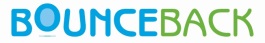
** to statutory and third sector mental health support services.

Caseworkers visited local jobcentre plus to discuss the **
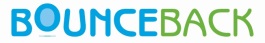
** project with staff and subsequently received further queries from DWP staff about the referral process and service. We were able to describe to DWP staff some of the constraints to some of our shared service users receiving their welfare benefits and, as a result, DWP will review how medical information is received in the future, i.e. they will accept supporting information from a caseworker rather than a GP.

We attended meetings with Liverpool CCG to explore how the **
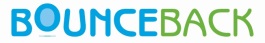
**project fitted with a changing policy and commissioning context. Liverpool CCG were seeking to develop a new model of integrated mental health services providing a Single Point of Access to services for patients. Our work resonated with their goals – in recognising a need for a person (rather than service) centred model of care. However local reorganisation and new initiatives meant that we recognised early on that integrating **
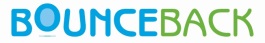
**into Liverpool practices was going to be hard. Especially as a new nationally funded pilot of placing Citizens Advice Bureau (CAB) workers into practices started at around the same time as our project.

**What we found**

Our outreach work identified much interest in our project and ways of working in principle, but also a number of outstanding problems including:

- A lack of clarity on how our model of care was DISTINCT from other local initiatives – including the CAB pilots in Liverpool and Mersey (creating barriers to engagement both in terms of uncertainty over appropriate referral and competitive anxieties between projects)
- A need to provide readily accessible, multi-level descriptions of the project suitable for different audience/stakeholder needs (from quick descriptions, to fuller justifications of the why and the how)

**What we did in response**

To progress our traffic lights from red to green, we engaged the services of a local community Public Relations firm to help us develop the resources we needed to address the identified concerns.

Scoping work led by AIW Health identified Artemis Media as our preferred contractor. Artemis is recognised as a leading 3^rd^ sector media relations expert and had worked for organisations such as National Youth Advocacy Scheme and Everton in the Community.

Artemis worked with us using marketing type approaches to facilitate a clearer description of the model of care we sought to deliver and a more focused engagement strategy. Including

- Developing a branding strategy for the service and project (how it is distinct)
- Developing a project logo and materials including a website [**www.bouncebackproject.co.uk**](http://www.bouncebackproject.co.uk) and practice materials including postcards, pop ups, leaflets (APPENDIX C), and a video describing the BounceBack project which was downloaded onto screens in group practice waiting rooms in order to provide a visual and interactive description of the service.
- Working with the press through the preparation of press releases
- Using social media (Twitter)**@BounceBackproj** to link with other research, campaigns and organisations for example Suicide Awareness.

**Figure x: Examples of
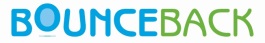
profile raising materials**


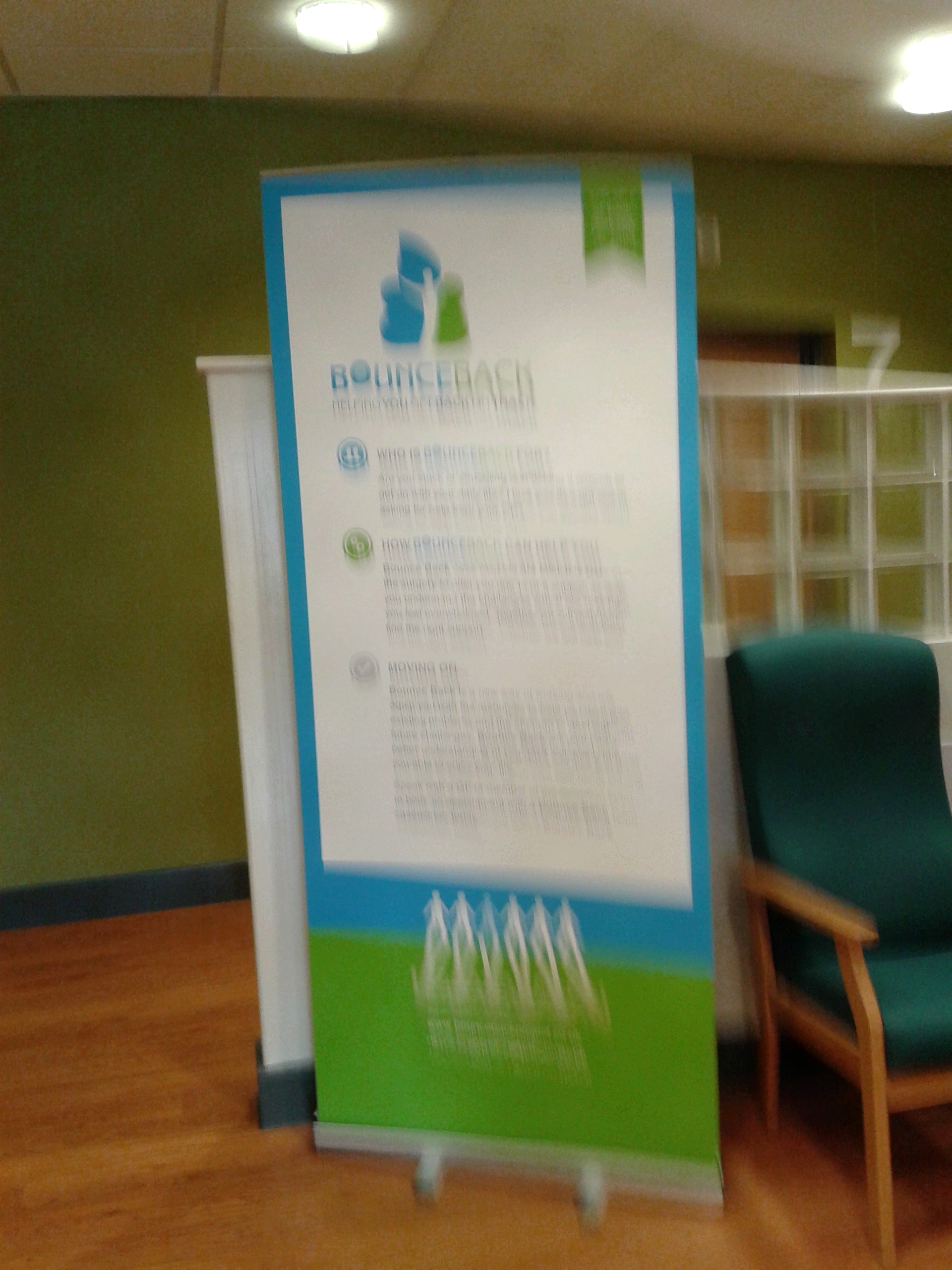


A pull up banner used in surgeries and at meetings


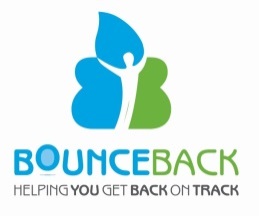


A logo used across all our materials

**What we learnt**

Preparing materials for different audiences required us to think very carefully about the project – what it is and why it is distinct. The process helped us refine our description of the core elements of our work (refine our understanding of the complex intervention), supported us in engaging with a wider body of stakeholders, as well as raising the profile of the project and so supporting engagement.

Lessons learned:

- Take time to describe a project to different stakeholders: what makes sense to you may not to them. Their uncertainties can help strengthen your proposal
- It takes longer than you think – factor it in to the project
- Work with staff who are used to writing for multiple audiences – they will help you do things differently, and often - better

**B2. ENGAGEMENT**

**Our goal:** to engage practices, patients, community staff and commissioners in our project – working with us to deliver, use and evaluate the new service

**What we did:**

*Engaging* *with the community:* see the outreach work described in B1. In March 2014, JR recorded an interview with Dr. James Kingsland (from our pilot group practice) on BBC Radio Merseyside’s Late Show which explored the need to move away from traditional models of care for those with mental health problems and described the model that **
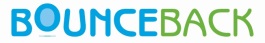
** sought to deliver. This interview resulted in a large volume of calls from listeners and was made available on the **
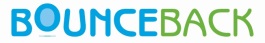
** website.

*Engagement with practices – initial engagement of a pilot site:* Because of local service changes, we moved the project back over the the Wirral. We were struggling to identify practices able to partner with the project. Eventually we identified a single pilot sight – SHB. The project team initially met with the practice team to discuss implementation and a single case worker has started working on site.

Caseworkers met first with Practice Managers to provide information about the project and the service, highlighting the practical requirements to deliver such a service; for example room availability, access to online medical reporting , telephone availability as well as issues such as how Primary care staff wished to refer people to the project and how they wished to be kept informed of the intervention.

We ensured that confidentiality was maintained and signed agreements within each practice which also allowed us access to medical systems such as EMIS and VISION, this also allowed caseworkers some flexibility in making appointments and allowed patients the option to self refer.

Caseworkers then met with Primary Care Teams in order to describe how and when the service would be delivered. We agreed regular session times, for example; starting with one morning per week which could be re-assessed and increased to meet with demand for appointments. We could also offer flexibility in order to fit in with the needs of the individual or of the group practice.

Initially we received a number of referrals of patients who merely required practical support such as employment law advice, help with benefit claim forms etc. We highlighted this to the Practice Manager and suggested a meeting with the other agencies already integrated into the practice: alcohol support services, Trent Psychological Service and PCAAL. It became apparent that there was some uncertainty about these agencies and their presence at SHGP. Caseworker attended regular clinical meetings at SHGP as this was the best opportunity to meet with GPs and Nursing Staff in order to explain the referral process and also to identify suitable candidates for referral and to address any referral issues we had. Meetings also took place with PCAAL in order to highlight how BounceBack project differed from PCAAL service. The caseworker identified that female GPs (in particular S2 trainees) appeared to have a better understanding of the project and were more open to discussion and referral (either face to face, by telephone or by e-mail), there were several telephone consultations with these GPs.

*Introduction of new practices*

Caseworkers identified (using census, OFSTED, JSNA reports and their own local knowledge & understanding,) areas across Wirral where **
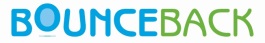
** project would benefit local populations e.g. regeneration areas such as Tranmere (which serves Whetstone, Greenway Practices) with higher ethnic and unemployed populations. We wrote to these Practices (sent information initially via Wirral CCG communications team to GPs) and wrote to Practice Managers to request meetings. In the case of Whetstone Lane Group Practice, we “cold called” and spoke to the Practice Manager to arrange a further meeting. AiW Health had previously worked in association with West Kirby Health Centre and therefore, the service offered (however, this was agreed though never pursued further by WKHC)

We provided new practices with information about the project (A4 leaflet and Postcard), describing the core components of the intervention, our therapeutic model and details of group practice requirements such as room availability, patient consent, access to electronic case notes etc. We used previous experience in other practices to ensure that referrals were appropriate – for example; we requested that caseworkers be provided with a brief overview of a new referral (or could contact the patient directly to discuss) rather than appointments being made without consultation/prior information as this would reduce the incidence of inappropriate referrals (these individuals could be signposted to other services)

Caseworkers met with Practice Managers and requested to attend clinical meetings so that the service could be discussed in detail with GPs and any questions answered. We provided information about the timeline of the project (in particular to practices such as Miriam and Earlston who were the last to engage) as we were still able to provide a service for a number of months.

At Whetstone we had a situation where the practice decided they didn’t want the caseworkers to have access to EMIS. Withdrawing EMIS meant that the caseworker was not able to make appointments – reception staff made appointments without consultation (e.g. a patient who had failed to engage, a patient who required practical help that could have been provided by PCAAL service or reception staff), it also meant that caseworker was not able to record an brief overview of the intervention as she had previously. Therefore, there was a lack of continuity. The caseworker reported any matters of concern either to the Practice Manager or to GPs via NHS e-mail account.

SHGP had a more informal (but effective) approach to clinical meetings which are held frequently and are well attended by all members of the primary care team. Reception staff were more proactive in contacting the caseworker with any queries/issues for discussion.

**What we learnt**

Across all our work in practice we identified a number of key areas of importance to successfully integrate.

1. The importance of having access to clinical/practice staff to encourage open discussion and communication about patients/practice related issues. To be fully integrated into a practice you need to be part of practice team and allow for a 2 way process. Build trust over time by getting to know caseworkers and how they work with patient and practice alike and keep up to date local knowledge
2. Continue to engage with potential new practices and local groups and services e.g. community outreach including statutory organisations such as the Department of Work and Pensions, Job Centre Plus, social housing partnerships, Citizens Advice Bureau & PCAAL, Retail outlets regarding debt. – we considered that visiting community outreach groups may help the referral process in practices such as Fender Way Health Centre were referral numbers were lower.
3. We engaged where appropriate with secondary care services – we advised GPs (e.g. in Whetstone Lane) of the need to refer individuals for secondary care services following assessments such as STORM, disclosure of alcohol abuse, in view of diagnosis of Personality Disorder etc. (AiW facilitated swifter referral to some groups due to their ongoing association with secondary mental health services)
4. We maintained a visual presence as much as possible in practices e.g. both physically in person and using marketing materials developed particularly in our initial site at SHGP – we were invited to Patient User Group Meetings, circulated newsletters and continued to maintain contact by e-mail with SU group chair.
5. Caseworkers kept in touch with all relevant service user groups and patient user groups – some service users agreed to use of their own case studies for our leaflets, for use on our radio broadcast, and one took part in person at our G.P. training event and they were consulted about a number of issues such as health questionnaires.
6. Recruitment of a second case worker - UoL and AiW worked together throughout the entire recruitment process. We re-visited the existing case worker job description and person specification and made the necessary changes to both documents, first analysing the qualifications and experience necessary to undertake the role. We wrote the job advertisement for local newspapers (Liverpool Echo, Metro) and devised questions to be asked at interview (first identifying the type of answers that we hoped to receive,) we devised a two week induction plan for the new case worker to ensure that learning and development needs were addressed. The second caseworker was appointed and commenced employment on 14^th^ April 2014.
7. Keep a record of ongoing issues the caseworkers faced and any areas of concern. This information was then fed back to the practice or the project team, where appropriate.
8. We developed a practice pack (describing our intervention, our policies and procedures, our therapeutic intervention and a list of practice requirements/practical things we needed) )Appendix E)
9. One of our caseworkers established a case discussion meeting within AiW that included the opportunity for peer support/supervision. This gave everyone involved an opportunity to share best practice and engage with others who didn’t have prior knowledge of the BounceBack service.

**B3. ACTION AND MONITORING**

We are able to describe the process of integration/embedding of a new needs assessment approach in primary care: including enablers and constraints action learning study of normalisation process, informed by NPT in order to describe how others could adopt the intervention. This information was then used to inform the development of next stage of integration plan.

Upon reflection, we recognise the importance of taking time to embed the project in a pilot practice. Especially as the ‘evidence needs’ have changed and commissioners want to know less about impact and more about ‘how to’

1. There was a change in context with regards to the CCG and the new CAB scheme, which led to ongoing discussions
2. Personnel issues: We faced a change in personnel with subsequent delays in appointing a second case worker.
3. There was a need to strengthen our project management and systems of internal reporting which addressed by increasing the frequency the team met face to face.
4. We faced a number of engagement issues in our pilot practice: this included lower than expected referral rates and confusion as to how this service fits in with and is different to others. Practical issues relating to room availability/appointment booking system also impeded progress and where addressed as they arose.

**APPENDIX C: PROJECT MATERIALS/LEAFLETS PRODUCED**

*
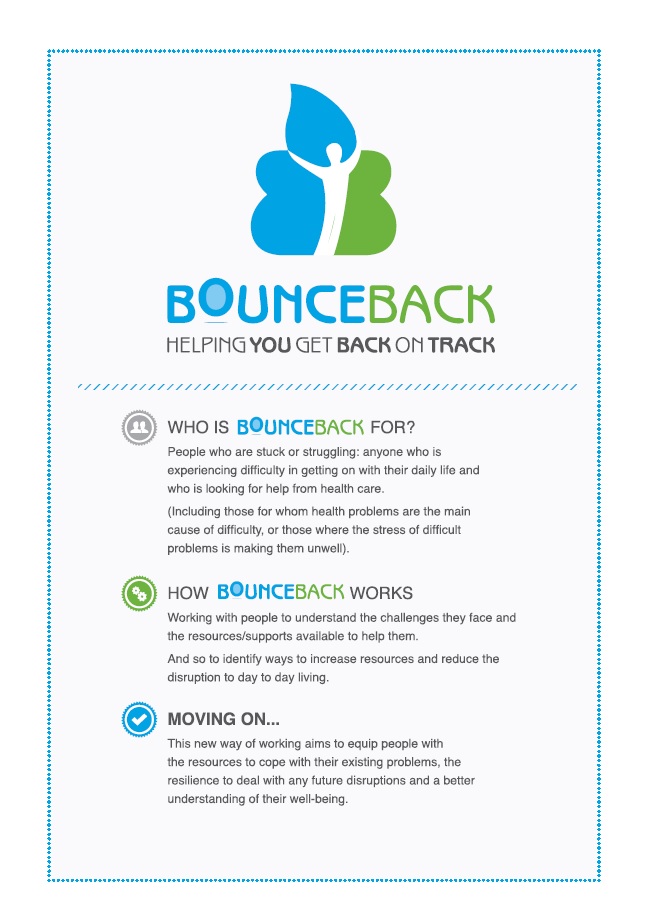
***C1: Project Leaflet**  *aimed at patients and Practice staff to explain the BounceBack service*

*
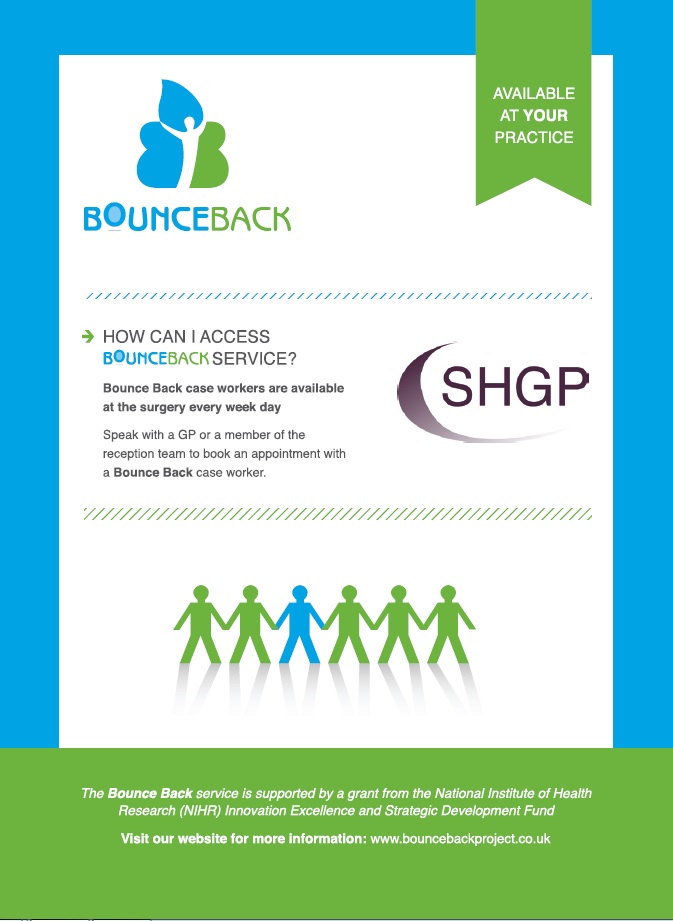
*

**C2: Service Leaflet *aimed at commissioners and policy makers to explain why the project mattered***

**
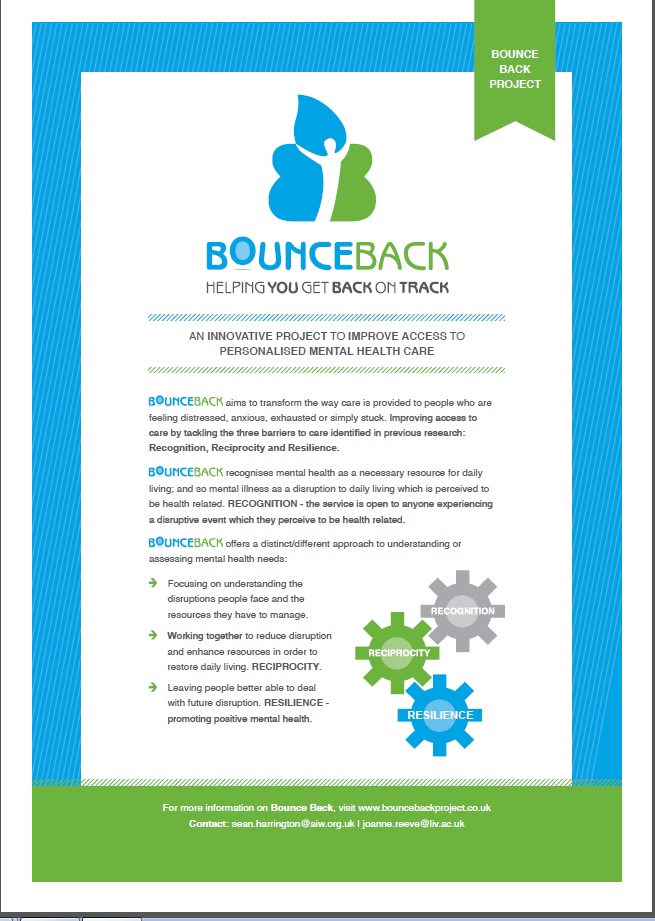
**

**
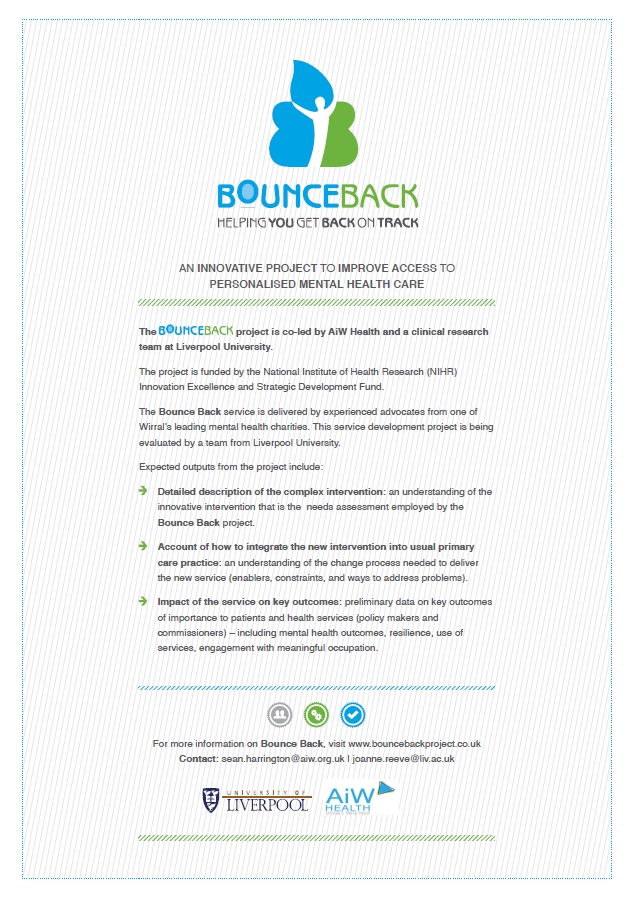
**

| \| - WHAT? BounceBack works with you to understand the disruption you are facing & the resources you have to manage. Leaving you better able to deal with future disruption.   **C3. Patient feedback leaflet**   - WHO? BounceBack caseworkers work within your GP practice and at AiW Health. - WHEN? BounceBack caseworkers are based at your GP surgery on at least one day per week – ask at reception for further details. - HOW?You can refer yourself to BounceBack (by @phone or e-mail *see contact us), or speak to your GP or member of the reception team. Appointments can be made at a time to suit you. \| \| --- \| \|  \| \|  \| \|  \| |  |  | *Contact us  [**jane.watkins@aiw.org.uk**](mailto:jane.watkins@aiw.org.uk)  **T:07847250396**  [**peter.rosbottom@aiw.org.uk**](mailto:peter.rosbottom@aiw.org.uk)  **T: 07948141087**  **St.Hilary Group Practice**  **Greenway Surgery**  **Whetstone Medical Centre**  **Fender Way Health Centre**  **Miriam Health Centre** |  | \| 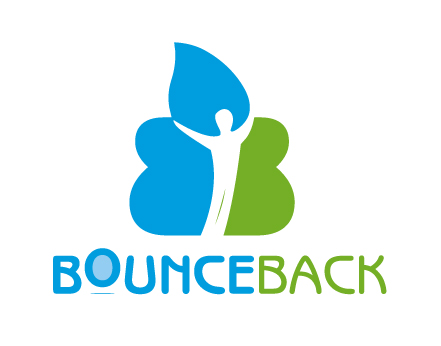 \| \| --- \| \|  \| \| **HELPED US GET BACK ON TRACK** \| \|  \| \|  \| |
| --- | --- | --- | --- | --- | --- | --- | --- | --- | --- | --- | --- | --- | --- | --- |

| **Carol…….**  **I was not coping very well with a few things: my**  **living arrangements and finances , it was a struggle**  **for me to stay alcohol-free and I felt really low.**    **My GP referred me to BounceBack - I told a**  **caseworker how I had been feeling and how**  **this had affected my ability to cope with everyday**  **things.**  **The caseworker helped me to identify and plan the**  **changes I wanted to make; we bid online at the surgery**  **for other properties and she helped me to apply for**  **other benefits that I didn’t know I was entitled to.**  **We also talked about what I enjoyed doing and how to**  **do more of these things.**  **The caseworker told me that some of these issues**  **might take more time than others so we kept in touch**  **mostly by phone or I could make an appointment to**  **talk to her at the surgery**  **I was re-housed to a lovely flat and my income has**  **increased - I don’t have to worry about how I’m going**  **to manage. Knowing that there was light at the end of**  **the tunnel helped me to cope and encouraged me to stay well.**  **I feel much more confident about managing things**  **myself .** | 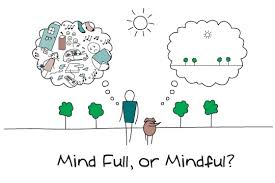 |  | Jonathon…..  I found BounceBack very helpful. I was  referred to the service through my GP for  help with my anxiety.  My caseworker helped me identify the  triggers around my anxiety. We prioritized  these triggers and put plans together. These  plans made me feel more able to tackle my  anxiety, because I felt that things were in  place.   It also helped me become aware of  allowing time to do things that I liked or  were of benefit to me,  like gardening, reading and relaxing, and  I began to exercise more.  I found the frequent appointments, and  the opportunity to talk things through very  beneficial. I was also able to contact the  caseworker by phone or email if I needed  to.  Over time, my anxiety was not  overwhelming me anymore, I looked at   things much more positively, and I was  able to return to work.  With the support from BounceBack  I now feel more able to cope with my  Anxiety and get on with my everyday life. |
| --- | --- | --- | --- |
|  |  |  |  |
|  |  |  |  |

**APPENDIX D Summarising total progress we made across the whole project in Phase 1.**

|  | **How we will integrate our model into primary care? (What do we need to do?)** | **What we have achieved?** | **Setbacks/Issues to date** |
| --- | --- | --- | --- |
| **SENSE MAKING/VISION**  How does GP practice* understand mental health (MH) and mental health needs assessment (MHNA) and how does this fit with the principles of our model  (*at whole system level) | Key outcome of Phase 1: The need for a clear description of the model of care: including why it is different  We also need to establish:   1. How does the **GP** practice understand MH & MHNA and how does it compare to our new way of doing things? 2. How does the **patient** understand MH & MHNA and how does it compare to our new way of doing things 3. How does the **organisational system** understand MH & MHNA and how does it compare to our new way of doing things | Description of the **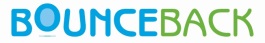** service and project that includes how it differs from any existing services and why it is innovative**✔**  Branding strategy developed for the service and project which includes a project logo and postcards **✔**  A pilot site was identified and approached (St Hilary Brow primary care practice, Wallasey) **✔**    We can describe the process of integration/embedding of a new needs assessment approach in primary care: including enablers and constraints **✔**  Framework has been developed to use in Phase One to monitor the service development stages. **✔**  Project management and reporting systems have been discussed and established **✔**  A database has been set up to record the relevant data **✔**  Develop and implement a marketing strategy. **✔**  We revisited our description of our new model in order to ensure it continues to make sense to PATIENTS, PRACTITIONERS and the SYSTEM **✔**  We understand the patient perspective: what their presentation and response tells us about mental health, needs assessment and care. Explore the role of the practice patients’ forum & do an educational session **✔**  With professional primary care staff: we have a greater understand of how they assess mental health need & how that influences action. We are aware of how that relates to our model, and the implications for changing practice. **✔**  Create a map of referral services available **✔**  **With GP’s**  1. Observation of different GP’s (with different approaches/at random/attitudes to MH) – observe consultations: sit in with GPs and observe how they deal with distress **✔**  2. Run a training session and observe practice responses in addition to questioning them. **✔**  **With Patients:**   1. Observe consultations: how they present and how they respond **✔** 2. Explore the role of the patients’ forum & and see if we are able to do a session with them. **✔** 3. Look at patient expectation and how that matches with their experience e.g. why they were there, did they get the care they wanted? Provide feedback for the GP. **✔** | Staffing delays: getting people in post & sickness. We currently only have one case worker, but there are plans to recruit a second in the near future  The has been a change in context with regards to the CCG and the new CAB scheme, there have been ongoing discussions  There has been a need to strengthen our project management and systems of reporting, now resolved  Unrealistic original deadline: We recognise the importance of taking time to embed the project. Especially as the ‘evidence needs’ have changed and commissioners want to know less about impact and more about ‘how to’ |
| **ENGAGEMENT**  are there people able and willing to initiate and continue a new way of working | We need to generate interest from Primary Care community  We need to engage with local CCG’s  We need to engage the Practice on a team level, a patient level and an organisational level to affirm they are both willing and able to accommodate change. | Five practices signed up to the model/need for change and taking part in the project **✔**  CCG recognise need for change to improve MH/service access **✔**  We are able to show that this project offers something innovative and additional to current services available **✔**  The practice manager in our pilot practice will report monthly outlining the number of appointments filled, number of DNA’s etc. to show how the service is used. **✔**  Record barriers to engagement (able and willing to accommodate change) and the work we have done to overcome this **✔**  Target GPs/clinicians: host educational event to explain new way of working **✔**  Scoping work to identify further practices interested in working with us and approach to invite **✔**  Prepare materials needed and invite pack for new practices: service/project flyers, protocol for practice **✔**  Engage with pilot practice to iron out any practical issues **✔** | Practical issues in pilot practice. |
| **ACTION**  RESOURCES: Does the practice have the resources to do things differently | By having two caseworkers in post  Having resources in place to deal with issues identified in client discussions  Establish monitoring/recording mechanisms in place to capture the learning;   - Practice/Team level - Patient Level - Organisational Level | One caseworker in post, has seen a small no. of clients **✔**  Practice referral/contact systems in place *if not fully operational* **✔**  Identify enablers and constraints to a new way of working **✔**  -Skills  -training needs  -time  -practical e.g. rooms  -referral/appointment set up  -continuity of care  For practice, patient and organisation  Produce shared project calendar detailing individual commitments/ actions **✔**  Prepare job spec for new case worker; review existing job spec. and advertise **✔**  Review project management systems: including user group **✔**  Observation of practice meetings **✔**  Survey/feedback from Caseworker/practice/patient **✔**  Case Study interviews for evaluation purposes **✔** | Personnel issues: one case worker short and project management aspects under resourced  -Small no. of clients being seen |
| **MONITORING & FEEDBACK:**  Does the practice get the feedback it needs to continue to do things differently | Evaluation strategy needs to be in place to collect data  Mechanism to share and reflect on data/learning on a;   - Practice/Team level - Patient Level - Organisational Level: having regular meeting with the practice | Project management group to meet monthly – set dates, recording processes etc **✔**  Service delivery and outcome monitoring tools prepared (survey tools and database) **✔**  Project evaluation framework in place and being used (traffic lights against NPT headings) **✔**  Observation of practice meetings **✔**  Collate scores from Questionnaires **✔**  Survey/feedback from BB Caseworker/practice/ patient **✔**  Record what are the positives and negatives to delivery **✔**  Prepare observation framework **✔** |  |

**APPENDIX E: MANUAL DESCRIBING BOUNCEBACK SERVICE DELIVERY**

The project describes a different approach to understanding mental health need that seeks to engage patients in being part of the solution to their problems - building resilience.

Our approach focuses on:

- *Understanding* the disruptive situation a person is in which contributes to their **distress** (an **imbalance** between the **demands** on them and the **resources** they have available to manage)
- *Working together* to find practical solutions to **reduce demands, enhance resources and so build resilience.**
- Leaving people better able to deal with similar problems arising in future. *Promoting positive mental health*.

**Getting the service started:**

**Partnership with other agencies and organisations:**

1. Promotion of service:
   1. Contacting practices, attending events and local service meetings, presentations in seminars and events, creating leaflets and flyers to send out via post and email, leaving information about the service in relevant local services, include service information in local media i.e. newspapers, radio etc.
   2. Increases both knowledge and awareness of the service, in turn increasing interest from potential practices and also options for self-referral.

**Requirements prior to Caseworker beginning in the practice:**

1. Meeting with lead GP/ practice manager:
   1. Ensures that the service being provided is fully understood. Describe core components and issue with information pack
   2. Allows a discussion on methods of communication, referral methods, room allocation and required environment to be able to work in a safe and therapeutic manner, hours of work, and level of flexibility at appointments.
2. Introduction to group practice staff and services:
   1. This includes identifying and liaising with any local community groups.
   2. Ensures understanding of the service amongst the primary care team.
   3. Promotes/facilitates referrals and signposting to both the service and other relevant professionals.
3. Access to electronic system for appointment logs and casenote recording i.e. Vision/ Emis:
   1. Allows best method for record keeping.
   2. Increases level of flexibility
   3. Increases effective communication between primary care team in turn improving continuity of care.
4. Access to NHS email account:
   1. Facilitates direct referrals to service via GP or member of primary care team.
   2. Allows caseworker to provide and/or review any relevant information regarding referral.
   3. Caseworker is able to book in any follow-up appointments/ self-referrals when necessary.

**Ongoing Requirements:**

1. Contact details of all practice staff:
   1. Facilitates direct contact between the caseworker and members of the primary care team
2. Regular review of the service:
   1. Through access to staff meetings and patient user groups.
   2. Helps to identify any operational issues.
   3. Allows feedback to and from all stakeholders.
   4. Provides information for evaluation purposes.
   5. Supports team in sharing best practice and continual improvement.
3. Continual Professional Development:
   1. Relevant and ongoing training opportunities to be provided and accessed through group practice, service provider, and external bodies.

**Caseworker job role and requirements:**

1. Job Role:
   1. To promote and support the integration (and evaluation) of the innovative approach to understanding and assessing mental health need into primary care practice.
   2. To work with clients to reduce their disruption and to enhance resources to restore daily living.
   3. To help clients build their resilience and a better understanding of well-being and positive mental health.
2. Duties and Responsibilities:
   1. Integrating into Primary care teams to deliver and monitor the service.
   2. Working in partnership with the client- provide information, advocacy and practical support.
   3. Acting on behalf of clients, where consent and agreement has been granted, taking action as directed by the client and consistent with good practice and the law.
   4. Communicating with individuals and other agencies to represent the client’s views, whilst keeping the client informed on the progress of their issues.
   5. Maintaining continued professional and personal and sharing best practice in order to promote and deliver the service in other group practices.
   6. Promoting the service t clients and relevant stakeholders by raising awareness and understanding of the innovation.
3. Caseworker Person Specification:
   1. Educated to A level / NVQ 3 standard with advocacy and/or health & Social Care qualification.
   2. An understanding of mental health illness and current trends/ issues around mental health.
   3. Experience of working in a mental health setting.
   4. Previous experience of working with vulnerable groups.
   5. Previous experience of providing practical support, advice and advocacy.
   6. A commitment to Equal Opportunities & anti-oppressive practice.
   7. Commitment to continued professional development.
   8. Excellent written and verbal communication skills – ability to produce written letters, reports and maintain client records.
   9. Ability to work independently managing time and caseload by prioritising tasks and meeting deadlines etc.
   10. Ability to work sensitively with clients & maintain confidentiality.
   11. Flexible and adaptable approach to work.

**Service Delivery:**

1. Initial Consultation:
   1. Understanding the disruptive situation a person is in which contributes to their distress
   2. Looking into the imbalance between demands on them and the resources they have available to manage.
   3. Signposting and referring to relevant services and professionals when necessary.
2. Follow-up consultations:
   1. Working together to find practical solutions to reduce demands, enhance resources, and so build resilience.
   2. Creating individually tailored decisions about care; creating a personalised explanation of the person’s distress and so suggestions for actions.
   3. Leaving people better able to deal with similar problems arising in the future, in turn promoting positive mental health.
3. Who BounceBack is designed to help:
   1. Individuals ages 16-65.
   2. People who are stuck or struggling: anyone who is experiencing difficulty in getting on with their daily life and who is looking for help from healthcare (including those for whom health problems are the main cause of difficulty, or those where the stress of difficult problems is making them unwell.
4. Who BounceBack doesn’t work with:
   1. Children and adolescents
   2. Individuals under secondary care for the problem/s that they require help with.
   3. Any individual with suicidal intent.
5. What types of services we refer and/or signpost to:
   1. Age UK
   2. AiW debt team
   3. AiW Drug and Alcohol Early Interventions Team
   4. Beating the Blues (cCBT)
   5. CAB/ PCAAL (Primary Care Advice and Advocacy Liaison)
   6. Cruse Bereavement Counselling
   7. Involve Northwest- Benefits advice
   8. MIND- Counselling and Group Services
   9. Response- Child and Adolescent Service providing information, advice and guidance to young people aged 13-19 years (linked with CAMHS)
   10. Tomorrow’s Women- Support for women who have been in the criminal justice system
   11. Wirral Women’s Refuge- Domestic Violence
   12. [www.get.gg](http://www.get.gg) – self-help website with information, tools and resources
   13. [www.llttf.com](http://www.llttf.com) – online self-help using CBT methods

**APPENDIX F: DATA COLLECTION TOOLS**

**WEMWEBS: Warwick-Edinburgh Mental Well Being Scale**^18^

The Warwick-Edinburgh Mental Well-being scale was developed to enable the monitoring of mental wellbeing in the general population and the evaluation of projects, programmes and policies which aim to improve mental wellbeing.

WEMWBS is a 14 item scale with 5 response categories, summed to provide a single score ranging from 14-70. The items are all worded positively and cover both feeling and functioning aspects of mental wellbeing.

**MAPA: Meaningful Activity Participation Assessment**^19^

Consists of 28 diverse activities. For which people indicated how often they take part and the degree of personal meaningfulness associated with each activity. Scores are combined multiplicatively: sum of the frequ3ency rating x meaning rating for each of 28 items. Giving a possible score range of 0-672. Higher scores indicate greater meaningful activity participation.

In a recent validation study in the US, the mean MAPA score was 214 (range 47-426) with reliable internal consistency and test-retest reliability.^19^

**CISR-20 (subscale fatigue)**^20^

A multidimensional questionnaire that consists of 20 items divided into 4 subdomains of fatigue and related behavioural aspects (subjective experience of fatigue, reduction in motivation, reduction in physical activity, reduction in concentration). The scale focuses on fatigue n the last 2 weeks. Scores range from 8-56, with the latter being the maximum level of fatigue.

**STUDY OUTCOME DATA COLLECTION**

Name ………………………………………………

Date of Birth ………………………………………………

Gender: Male/Female

We hope**
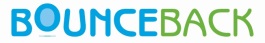
** will help you deal with the problems you are struggling with at the moment. We want to make sure that what we do is as useful as it can be. To help us make our service better, we need your help.

By answering some questions for us now, and at the end of your time working with your Bounce Back caseworker, you can help us keep improving the service we offer to patients

The following pages ask you a range of questions about your health and well-being, your feelings and your emotions. Unless otherwise stated please choose only ONE response from the options available.

We understand that it is not always easy to choose an option that describes exactly what you are feeling, and if you are unsure of which response to give, please choose the response that comes closest to how you feel.

If possible, we’d like you to fill in the questionnaire now whilst you are still at your GP practice. If you haven’t got time, you can take it home with you and post it back to us. If you forget, we will ring you up to remind you.

Please answer all the questions, there are no right or wrong answers.

Please enter today’s date 🗖🗖/🗖🗖/20🗖🗖

*These questions ask you about your thoughts and feelings*

Here are 14 statements which describe the way people might be feeling. For each one, Please circle one number that best describes how *you’ve* been feeling over the last **2 weeks**

**Example:** For question 1, if you have been feeling optimistic about the future *often,* please circle number *4*.

| None of the time | Rarely | Some of the time | Often | All of the time |
| --- | --- | --- | --- | --- |
| I’ve been feeling optimistic about the future | | | | |
| 1 | 2 | 3 | 4 | 5 |
| I’ve been feeling useful | | | | |
| 1 | 2 | 3 | 4 | 5 |
| I’ve been feeling relaxed | | | | |
| 1 | 2 | 3 | 4 | 5 |
| I’ve been feeling interested in other people | | | | |
| 1 | 2 | 3 | 4 | 5 |
| I’ve had energy to spare | | | | |
| 1 | 2 | 3 | 4 | 5 |
| I’ve been dealing with problems well | | | | |
| 1 | 2 | 3 | 4 | 5 |
| I’ve been thinking clearly | | | | |
| 1 | 2 | 3 | 4 | 5 |
| I’ve been feeling good about myself | | | | |
| 1 | 2 | 3 | 4 | 5 |
| I’ve been feeling close to other people | | | | |
| 1 | 2 | 3 | 4 | 5 |
| I’ve been feeling confident | | | | |
| 1 | 2 | 3 | 4 | 5 |
| I’ve been able to make up my own mind about things | | | | |
| 1 | 2 | 3 | 4 | 5 |
| I’ve been feeling loved | | | | |
| 1 | 2 | 3 | 4 | 5 |
| I’ve been interested in new things | | | | |
| 1 | 2 | 3 | 4 | 5 |
| I’ve been feeling cheerful | | | | |
| 1 | 2 | 3 | 4 | 5 |

*These questions aim to get an idea of what activities you are able to do at the moment, and how much time you spend on them*

Please rate the amount of **Time** that you spent on the following activities during the last few months. Circle one number.

Example: For question a. If you spend time on home making and maintenance *once a week*, please circle number *5*.

| Not at all | Less than once a **month** | Once a **month** | 2-3 times a **month** | Once a **week** | | Several times a **week** | **Every day** |
| --- | --- | --- | --- | --- | --- | --- | --- |
| Home Making & Home Maintenance | | | | | | | |
| 1 | 2 | 3 | 4 | 5 | 6 | | 7 |
| Personal Finances | | | | | | | |
| 1 | 2 | 3 | 4 | 5 | 6 | | 7 |
| Driving | | | | | | | |
| 1 | 2 | 3 | 4 | 5 | 6 | | 7 |
| Using Public Transportation | | | | | | | |
| 1 | 2 | 3 | 4 | 5 | 6 | | 7 |
| Medical Visits | | | | | | | |
| 1 | 2 | 3 | 4 | 5 | 6 | | 7 |
| Socialising | | | | | | | |
| 1 | 2 | 3 | 4 | 5 | 6 | | 7 |
| Writing Letters/Cards | | | | | | | |
| 1 | 2 | 3 | 4 | 5 | 6 | | 7 |
| Helping Others | | | | | | | |
| 1 | 2 | 3 | 4 | 5 | 6 | | 7 |
| Gardening | | | | | | | |
| 1 | 2 | 3 | 4 | 5 | 6 | | 7 |
| Physical Exercise | | | | | | | |
| 1 | 2 | 3 | 4 | 5 | 6 | | 7 |
| Crafts/Hobbies | | | | | | | |
| 1 | 2 | 3 | 4 | 5 | 6 | | 7 |
| Cultural Activities | | | | | | | |
| 1 | 2 | 3 | 4 | 5 | 6 | | 7 |

| Not at all | | Less than once a **month** | | Once a **month** | | 2-3 times a **month** | Once a **week** | | | Several times a **week** | | **Every day** |
| --- | --- | --- | --- | --- | --- | --- | --- | --- | --- | --- | --- | --- |
| Musical Activities | | | | | | | | | | | | |
| 1 | | 2 | | 3 | | 4 | 5 | 6 | | | | 7 |
| Taking Courses | | | | | | | | | | | | |
| 1 | | 2 | | 3 | | 4 | 5 | 6 | | | | 7 |
| Creative Activities | | | | | | | | | | | | |
| 1 | | 2 | | 3 | | 4 | 5 | 6 | | | | 7 |
| Travelling | | | | | | | | | | | | |
| 1 | | | 2 | | 3 | 4 | 5 | 6 | | | | 7 |
| Talking on the telephone | | | | | | | | | | | | |
| 1 | | | 2 | | 3 | 4 | 5 | 6 | | | 7 | |
| Reading Magazines/Newspapers | | | | | | | | | | | | |
| 1 | 2 | | | | 3 | 4 | 5 | | 6 | | 7 | |
| Other reading | | | | | | | | | | | | |
| 1 | 2 | | | | 3 | 4 | 5 | | 6 | | 7 | |
| Playing Games | | | | | | | | | | | | |
| 1 | 2 | | | | 3 | 4 | 5 | | 6 | | 7 | |
| Watching TV/Listening to the radio | | | | | | | | | | | | |
| 1 | 2 | | | | 3 | 4 | 5 | | 6 | | 7 | |
| Religious Activities | | | | | | | | | | | | |
| 1 | 2 | | | | 3 | 4 | 5 | | 6 | | 7 | |
| Prayer/Meditation | | | | | | | | | | | | |
| 1 | 2 | | | | 3 | 4 | 5 | | 6 | | 7 | |
| Community Organisation Activities | | | | | | | | | | | | |
| 1 | 2 | | | | 3 | 4 | 5 | | 6 | | 7 | |
| Volunteer Activities | | | | | | | | | | | | |
| 1 | 2 | | | | 3 | 4 | 5 | | 6 | | 7 | |
| Pet Care/ Activities | | | | | | | | | | | | |
| 1 | 2 | | | | 3 | 4 | 5 | | 6 | | 7 | |
| Computer Use for email | | | | | | | | | | | | |
| 1 | 2 | | | | 3 | 4 | 5 | | 6 | | 7 | |
| Other computer use | | | | | | | | | | | | |
| 1 | 2 | | | | 3 | 4 | 5 | | 6 | | 7 | |
| Paid Work | | | | | | | | | | | | |
| 1 | 2 | | | | 3 | 4 | 5 | | 6 | | 7 | |
| Any comments? | | | | | | | | | | | | |

These questions ask about how you feel your energy levels are at the moment

Please circle one number that best describes your experience of each question over the last **2 weeks**

| Yes that is TRUE |  |  |  | No, that is NOT TRUE |
| --- | --- | --- | --- | --- |
| I feel tired | | | | |
| 1 | 2 | 3 | 4 | 5 |
| I feel very active | | | | |
| 1 | 2 | 3 | 4 | 5 |
| Thinking requires effort | | | | |
| 1 | 2 | 3 | 4 | 5 |
| Physically I feel exhausted | | | | |
| 1 | 2 | 3 | 4 | 5 |
| I feel like doing lots of nice things | | | | |
| 1 | 2 | 3 | 4 | 5 |
| I feel fit | | | | |
| 1 | 2 | 3 | 4 | 5 |
| I think I do a lot in a day | | | | |
| 1 | 2 | 3 | 4 | 5 |
| When I am doing something I can keep my thoughts on it | | | | |
| 1 | 2 | 3 | 4 | 5 |
| I feel powerless | | | | |
| 1 | 2 | 3 | 4 | 5 |
| I think I do very little in a day | | | | |
| 1 | 2 | 3 | 4 | 5 |
| I find it easy to focus my mind | | | | |
| 1 | 2 | 3 | 4 | 5 |
| I feel rested | | | | |
| 1 | 2 | 3 | 4 | 5 |
| It takes a lot of effort to concentrate on things | | | | |
| 1 | 2 | 3 | 4 | 5 |
| Physically I feel like I am in bad form | | | | |
| 1 | 2 | 3 | 4 | 5 |
| I have lots of plans | | | | |
| 1 | 2 | 3 | 4 | 5 |
| I tire easily | | | | |
| 1 | 2 | 3 | 4 | 5 |

| Yes that is TRUE |  |  |  | No, that is NOT TRUE |
| --- | --- | --- | --- | --- |
| I get little done | | | | |
| 1 | 2 | 3 | 4 | 5 |
| I don’t feel like doing anything | | | | |
| 1 | 2 | 3 | 4 | 5 |
| My thoughts easily wander | | | | |
| 1 | 2 | 3 | 4 | 5 |
| Physically I feel I am in an excellent condition | | | | |
| 1 | 2 | 3 | 4 | 5 |

Overall, how **satisfied** do you feel about the care you have received from your BounceBack worker?

**1 2 3 4 5**

(1 = unsatisfied, 2 = somewhat satisfied, 3 = rather satisfied, 4 = quite satisfied and 5 = very satisfied).

Thank you for your help and time

**APPENDIX G: CONTEXTUAL INFORMATION**

(Background info informing case study analysis)

| **Practice id** | **GPs in practice*** | **Number of registered patients** | **Nature of local population** | **Practice quality markers** |
| --- | --- | --- | --- | --- |
| P1 | 2M, 2F | 5155 | Residential area, in 5^th^ most deprived decile in Engalnd | QOF score = 974, 90.9% patient satisfaction (PS)** |
| P2 | 3M, 4F | 8811 | Ethnically diverse (high level english as 2^nd^ language), 1^st^ decile deprivation (most deprived) | QOF=980, PS = 79% |
| P3 | 1M, 4F | 7956 | Less ethically diverse, in 2^nd^ most deprived decline | QOF=995, PS=91.7% |
| P4 |  |  |  |  |
| P5 | 1M, 3F | 3692 | Former council owned, low owner occupation | QOF=892, PS=78.5% |
| P6 | 1M, 5F | 5158 | Residential area, 1^st^ most deprived decile | QOF 997, PS 89.2% |
| P7 | 1M, 5F | 4232 | Less ethncally diverse, 4^th^ most deprived decile | QOF 997, PS 78.9% |

* M=male, F=female

** QOF = quality outcomes framework score for practice (maximum =1000); patient satisfaction = proportion of patients who would recommend practice to others

**APPENDIX H: PROJECT FEEDBACK (anonymised)**

**Sent:** 18 February 2015 13:27
I just wanted to thank you for all your help, and supporting me through a difficult time in my life. Also I want to apologize for not being in touch. I have made loads of progress and I am managing to stay on top of my rent. I didn't return to work at Cambrian house it wasn't the place for me any more. I am currently waiting for contracts to be drawn up for me to go and work as an au pair in Sussex for a lovely family in a lovely and peaceful setting and I will be earning a good wage . I was thinking about you last night and thought it wasn't very polite of me to not have been in touch with you at all and I want to apologize for that.Yours sincerely,

**Sent:** 09 February 2015 18:56
I'm sorry the project is ending as I found it really helpful. Can you fit me in tomorrow?  I am at home at the moment but possibly starting work next week or going on a visit to my daughter.

**Sent:** 15 January 2015 07:52
Thank you once again for your time on tuesday, and thank you for helping me through a very difficult time I really appreciate it. Anyway I will speak to you about the outcome next tuesday, and in the meantime, thank you once again for your support.

**Sent**: 2 Jan 2015, at 13:23

Thanks for that. I wanted to thank you for putting things into perspective for me. Our chat gave me a real boost and I'm really grateful.

**Sent:** 17 December 2014 22:08
It is great to feel that I have someone to help me through the anxiety I feel about my current situation,

**Sent**: 28 October 2014 10:15
Just wanted to say thanks for seeing patient KR today, he feels a lot better after talking to you.

**Sent**: 10 October 2014 06:19
Thank you so much for seeing A O so quickly and so flexibly closer to his home. I am glad that he has involved his wife in the discussion too, it was my hope that he would do so as this will augment his resolve to manage his situation. Swimming sounds to be an excellent exercise intervention that will not aggravate his knee problem, being a non load-bearing activity. I would be delighted to help with supporting medical evidence, pending his consent, that I feel sure will be given

**Sent:** 20 May 2014 15:41
Received a letter from the income tax saying they have accepted my appeal. Great stuff Thanks again for your expertise  and patience

**Sent**: 23 January 2014 16:36
I felt really positive after our appointment yesterday, Don't want to get ahead of myself but big step, regards T

**Sent:** 28 February 2014 11:35
Many thanks for your help with her. I saw her this morning and she was a lot less anxious after you had explained a few things.

**Sent:** 02 June 2014 22:32
Thank you so much for letting me know the outcome I am delighted for him. Thanks again for your help in ensuring I did the correct report !

**Sent:** 08 October 2014 08:29
That's brilliant , I am most grateful for your prompt and fantastic input.

Sent: 10 November 2014 22:43
Just thought I would email you to say I am feeling a lot better than I have been feeling recently cbt has really helped a lot I have learnt how to control things and situations a lot better so thank you see you soon

**Sent:** 27 February 2015 16:16
I attended a meeting with KT this week on the 25^th^ February. The meeting was held at [practice name] and was a Patient Participation Group. During the meeting the Practice Manager spoke about the good work that has been done by the Bounce Back Team. [S/he] spoke about how valuable this service has been.

**Sent: 27 February**

I was referred by my GP.  I went to see my GP with stress and depression. At the time I was feeling overwhelmed by the demands made on me from work and caring responsibilities to my elderly mother. I would normally turn to my partner for support; he will often help with some of the demands made by my mother.  I was particularly overwhelmed by work at the time and felt unable to carry out my responsibilities in social care when faced with major cutbacks to funding and an ever increasing case load.  I had also been unfairly treated by a senior manager and had to face a grievance hearing which subjected me to further stress.  I was managing my mother’s financial affairs and having to take legal action against tenants in her property who were not paying the rent which was funding part of her care. I have a daughter and granddaughter who live over 200 miles away and I try to make time to visit them and offer practical help.  Although I enjoy the visits it is difficult to fit them around work and caring commitments.  I had no practical difficulties of my own.

I wanted the service to provide an alternative to medication that would improve the way I felt and my ability to cope.  The service offered someone to talk to outside of my immediate family and other ways of looking at managing my problems. I took practical advice from my caseworker as well as support to complete the CBT course.

Since my last appointment my mood has improved and i recognise and challenge negative thoughts if i start to feel low.  I have been offered and taken voluntary redundancy.  I have a new job and, although still demanding the hours and responsibilities are less. I am dealing with my mother's caring and financial responsibilities better.  I don't feel I have had any setbacks.  I cope better now I understand my own thinking around these better.  I have managed problems by making some "me" time and planning practical ways of dealing with what I can change and changing ways of thinking about what I cannot.

The most useful thing about the service was offering strategies to change thought processes, see things differently and taking some practical action to improve my situation. The least helpful aspect initially was the further demand of the CBT course.  I would recommend the service because it achieved its aim for me of offering an alternative and practical solution to medication.


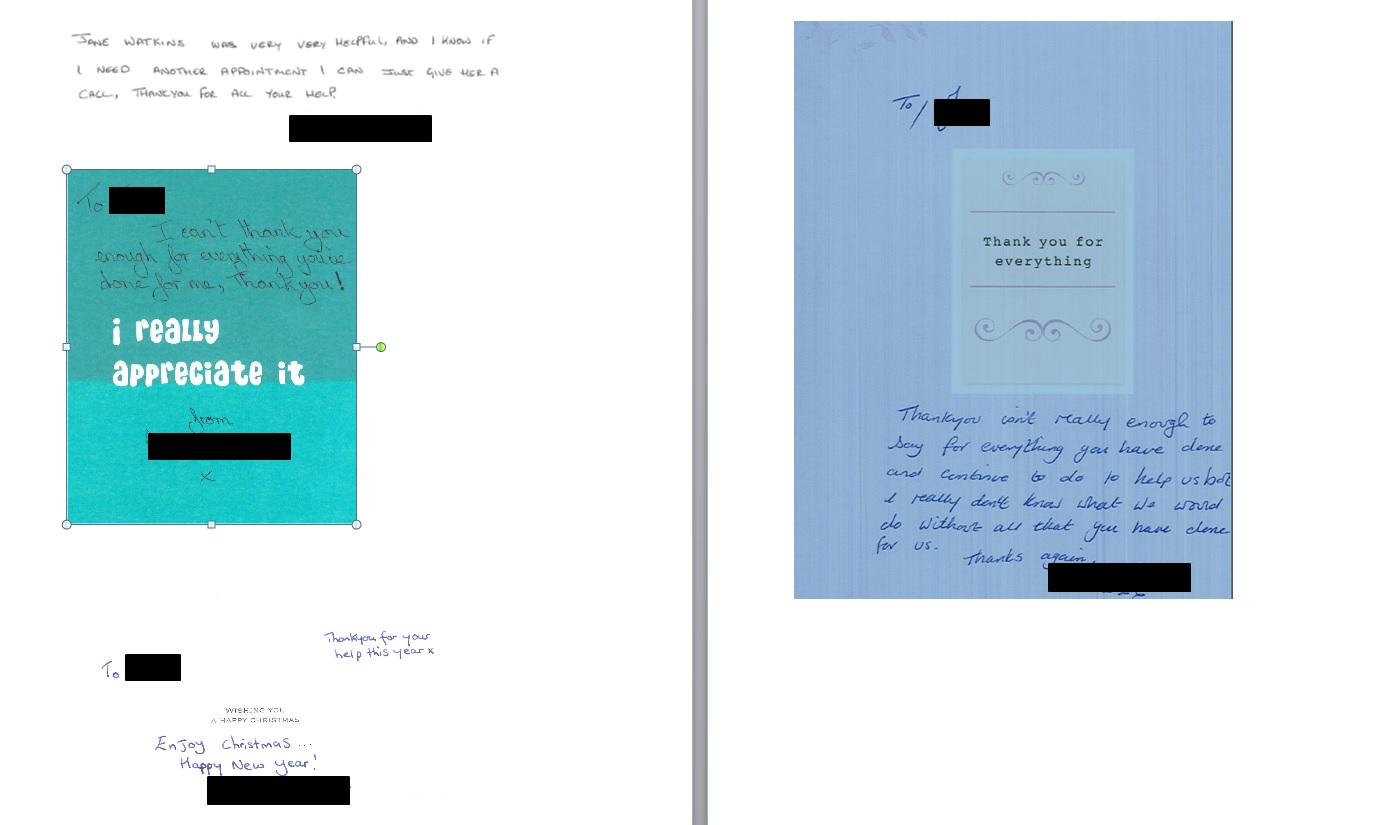


**APPENDIX I: PHASE 2 CASE STUDY DATA**

**Dose delivered:** Observation data October 2014. Observer = LC (mental health training, plus researcher observation), assessed care against the described BounceBack model

| **Observed Characteristics** | JW (1-4 are in chronological order over a period of about a month (sept-oct 2014) | PR |
| --- | --- | --- |
| Unstructured consultation (data collection doesn’t privilege one type) | JO1; steered by pt, includes service and patient story factors (no tools/measures but more implicit agenda from the patient?)  JO2: Good use of open questions to create a lot of data to work with  JO3: labels (medical – AD and CBT) brought by the patient  JO4: CBT, Ads brought up by patient again – they are structuring the consultation | PO1: tried but struggled. Pt not talkative and passive  PO2: open questions, pt driven (f on appt)  PO3: with use of curious questions to drive/support this  PO4: pt came I with biomedical agend (having a depression blip, stopped ads and feeling awful etc). Found it hard to open out |
| Biographical lens | JO1: focus on family, pressures and associated consequences  JO2: strong biographical lens: family and work for them  JO3: open questions, focus on work and beyond (the patients initial agenda being Ads and CBT)  JO4: again, well covered | PO1: exploring impact of work and sexuality  PO2: exploring context of daily life – impact and drivers  PO3: with use of curious questions  PO4: covers wider context to an extent but striggles to come away from medicine |
| Exploration (im)balance demands and resources | JO1: more focus on demands, not on the (potential) resources  JO2: not really exploration of see saw – stayed on open conversation of feeling stuck and overwhelmed with limited creation of story of how/why and so change  JO3: a bit more see saw evident now (3 weeks on from JO2)  JO4: subtle start of exploration of these issues | PO1: unable to do – pt not engaging?  PO2: good exploration of imbalance and use of resources – impact of resource use (positive effects from previously identified changes)  PO3: exploration reveals that more balanced now as a result of previous work  PO4: again – some, but with pt saying what already does, limited exploration of new opps |
| Identifies opportunities for modification | JO1: limited exploration of opps for self – more on what service could do for her (waiting for CBT etc)  JO2: limited  JO3: goal identified – to work on self esteem  JO4: patient not there yet | PO1: referral on to debt and alcohol services but not offering any ways forward herself  PO2: further modifications identified  PO3: at feedback stgae – feeing back on impact of modifications hav previously identified  PO4: struggled to get away from medical agenda? |
| Candidacy, concordance, resilience | JO1: Candidacy and concordance – willing/able to access service but less resilience focus, on building own capacity etc. Could be that just early in the process (follow on apt)  JO2:  JO3: seeing shift to resilience focus now  JO4: still at the candidacy stage as pt is making sense of it? | PO1: hard to judge. Resistant patient? Seeking fix, pt not engaging or Peter’s skills lacking? (and/or just too early to observe). Engagement issues so –ve on all 3  PO2: seeing as candidate, is trying to work with, resilience not yet but  PO3: has reached resilience stage – pt identifying things has tried, and will continue to do to maintain wellbeing  PO4: probably not there yet |
| Patient characteristics | JO1: 27yr old female follow on appt. Limited resilience, coping. Looking for answers/fixes. Awaiting psychology appt  JO2: female, 42 yrs, feeling stuck. Family and work commitments, overwhelmed  JO3: female, age?, single, living with mum and dad, new job as a stepping stone to better. Depression and anxiety picture. “GP wont increase Ads”  JO4: single male, 39 yrs, depression and anxiety. Been referred for CBT and Ads increased. Holding referral? Social isolation – very passive/dependent (mum at consultation) | PO1: 22yr old female. Work and sexuality related distress  PO2: female, age?, 2^nd^ appt, anxiety issues  PO3: ?gender, age, returner (had 7-8 appt, final one)  PO4: first appt, female. Withdrawal symptoms from citalopram (medical agenda at start)m |

**APPENDIX J: SLIDES FROM PRESENTATIONS**

J.1 SAPC CONFERNECE


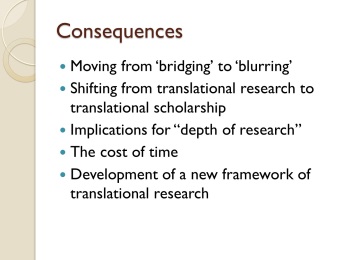

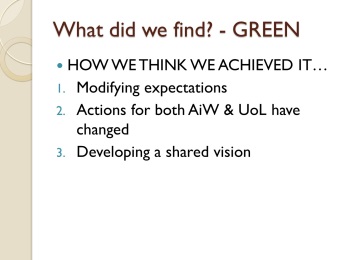

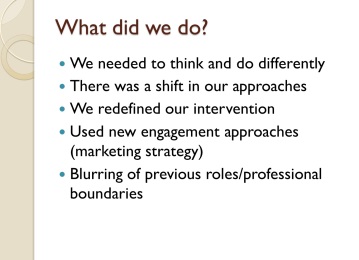

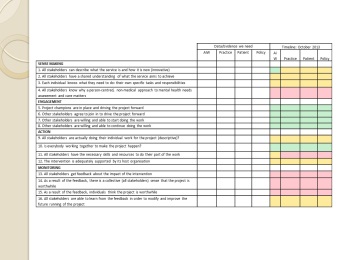

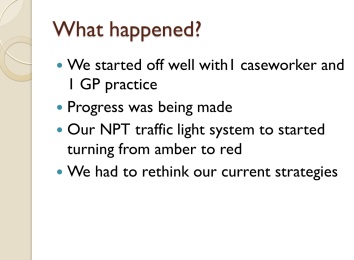

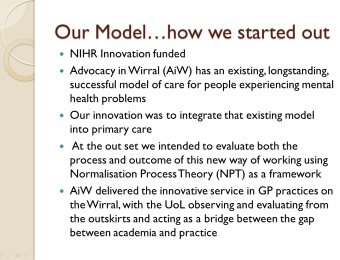

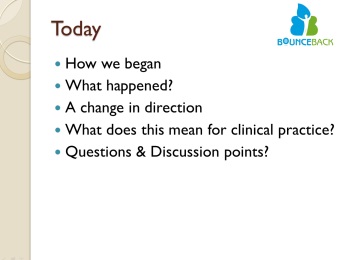

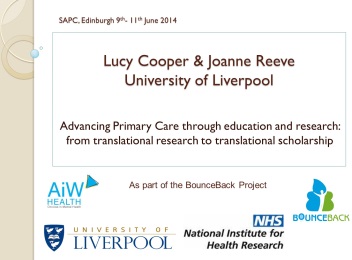


J.2 Manchester Seminar


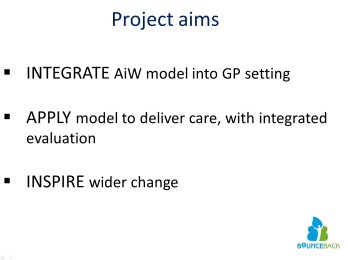

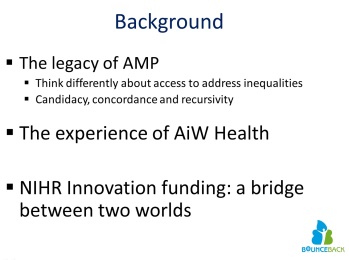

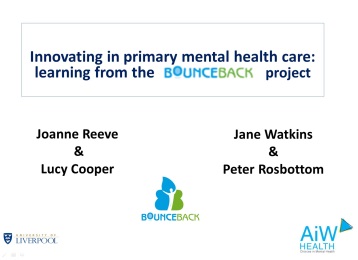


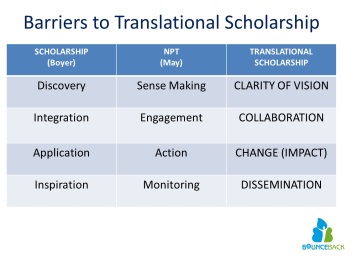

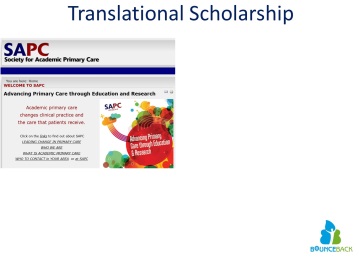

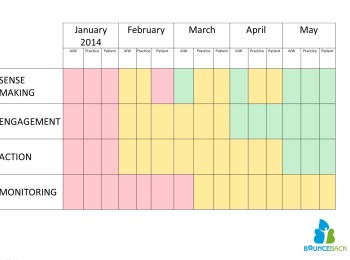

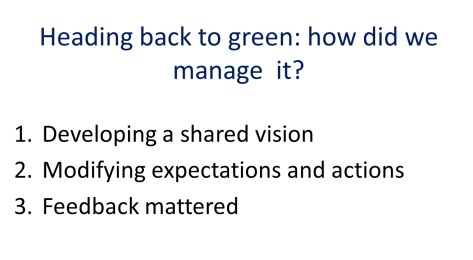

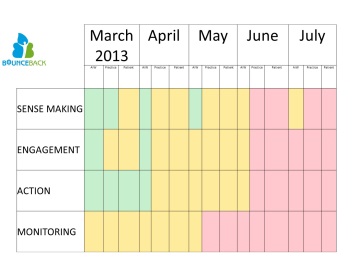

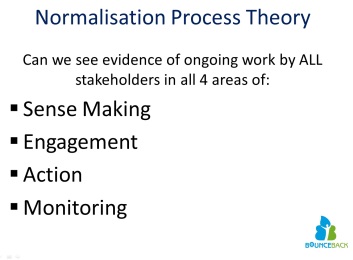

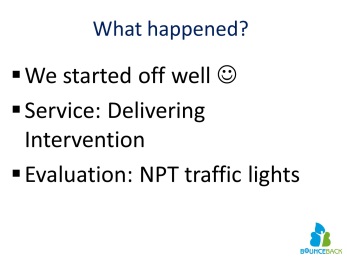


**APPENDIX K: INFORMATION PACK TO GP DELEGATES**

**Thinking Differently about mental health consultations**

**A reminder why this matters**

As generalists, we would all agree that people need to be able to access the right mental health care for their individual needs.

Previous research, including work led by people in Liverpool^[[2]](#footnote-2)^, has shown that getting this wrong means that we can over diagnose depression in some people^[[3]](#footnote-3)^. Whilst in others, we underdiagnose – and fail to recognise and manage their distress. Getting it wrong contributes to inequalities in health and health care^1^.


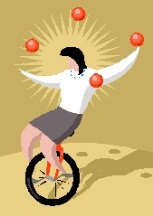
The **
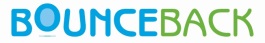
**project describes a different approach to understanding mental health need that seeks to engage patients in being part of the solution to their problems - building resilience.

Our approach focuses on

-
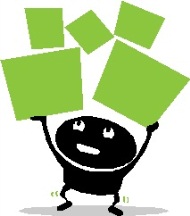
*understanding* the disruptive situation a person is in which contributes to their **distress**

(an **imbalance** between the **demands** on them and the **resources** they have available

to manage)

- *working together* to find practical solutions to **reduce demands, enhance resources and so build resilience.**
- Leaving people better able to deal with similar problems arising in future. *Promoting positive mental health*.

**Some resources for you to use**

Based on your feedback from our morning together, we have put together some ‘Top Tips’ – ideas for you to try out in practice. We welcome your feedback on whether they are useful.

Our Top Tips include:

- Helping the patient make sense of their distress: the **
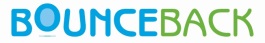
** story
- A 5 step consultation model (based on the SAGE generalist consultation model) – with ideas for each step to try out

**Making sense of distress: a story to use**

The story we have in our head when we talk with patients matters. It changes the way we interpret what they tell us. And so it changes the stories we tell with our patients.

At our FDA event, we talked about changing our way of thinking about distress – moving from a biopsychosocial model to a **socio-psycho-bio** model.^[[4]](#footnote-4)^

Many of us were trained in a biopsychosocial approach: to check whether someone has biomedically defined depression, and then explore the psychological and social implications of that diagnosis to help decide treatment (antidepressants or counselling)

Our approach turns this the other way round: starting with a ‘social’ story of disruption – from which we understand the psychological responses/impact; only coming to the medical if we need to…


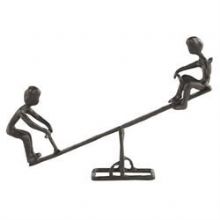

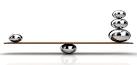


Next time you speak with a patient who is distressed, instead of starting with a medical story, you might try using this framework to help you explore and understand their distress.^[[5]](#footnote-5)^

*Practice Tips*:

- Try discussing this cycle with the next patient you think it would be suitable. Note down their response, questions.
- Try using both stories (remember the role plays) with patients and observe the impact on the consultation…
- Try using your see saw postcard as a prompt for you and your patient

**Talking with patients: a consultation model**

You asked us for some practical suggestions about putting this into practice: examples of questions we use, ways we frame the story. Drawing on the best traditions of GP training, we put together a consultation model for you to have a think about.

Five steps to use in a consultation^[[6]](#footnote-6)^

1. The lens through which we view the consultation: Disruption to daily living (see saw)


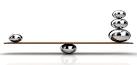

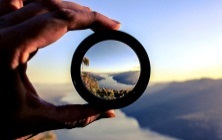
When a patient comes to see us, our goal is to work with them to make sense of the disruption/distress that they are experiencing. The explanation we construct will depend on the position from which we view a problem, or an experience. We can see it as a medical (biopsychosocial) problem or a **disruption to daily living** (sociopsychobio problem – see above).

1. Info gathering (question to explore the imbalance and understanding)

Our goal is to understand the (im)balance of demands and resources: **how out of balance are things?** How long has it been like that for? What are the resources and demands on each side of the see saw? What impact are these having in their everyday life?

Examples of questions and statements you might use to explore these questions

“You sound exhausted” or “That sounds exhausting” *to open up a conversation about current imbalance*

*Exploring resources:* “When was the last time you did something that made you smile?” Getting the basics right – eating, sleeping etc. Who is at home/work/etc. with you, have you had any of these problems before? If so, how did you manage then?


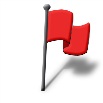
*Exploring drains:* Ask explicitly about work, home, money, and caring roles, any significant life events. Feeling overwhelmed (red flags)

Describe the ‘social story of depression’ and ask (if they haven’t already interrupted you) “*Does that sound like what you’ve been experiencing?”*

Through exploring these questions you will find you have also covered the biopsychosocial

1. Individually Tailored decisions about care

The expertise of the generalist is to weigh up all the info available to us to create a **personalised** explanation of this person’s distress and so suggestions for actions. So if the social model feels right for this individual…

- 1. Describe model and the specific Action plan arising – specific tasks for the patient
  2. Follow up is important

1. Checking in: have you missed something, or has something changed?

Safety netting for yourself. Ask yourself the questions: *does my explanation offer a reasonable understanding of everything the individual has told me? Would I make the same decision with someone else and if not, why not? Is my decision really based on this patient’s unique perspective, or has something else got in the way - my own views, preferences, prejudice even?*

1. Following up – reviewing impact

Gabby and le May^[[7]](#footnote-7)^ remind us that shared professional reflection is important for generalists who are working ‘beyond a standard protocol’. Two main sources for this

- 1. With the patient – bring them back, review the plan
  2. With colleagues – e.g. in practice team, groups like GP Forum meetings. Or even a Balint group(see resource list below)

**Other resources to look at**

<http://www.wellbeingliverpool.org.uk/index.php>

<https://www.futurelearn.com/courses/mental-health-and-well-being>

<http://www.amazon.co.uk/Beyond-Depression-approach-understanding-management/dp/0199545294>

Wirral Mind: Provides services for people experiencing mental health problems including: befriending, supported housing, counselling etc

[www.wirralmind.org.uk](http://www.wirralmind.org.uk)

Age Uk: Working with and for older people: financial advice, health & well-being, home and caring www.ageuk.org.uk

Involve North-West: benefits/employment advice, mediation, reach out, debt service etc.

[www.involvenorthwest.org.uk](http://www.involvenorthwest.org.uk)

Wirral Change: Black and racial minotities outreach service offering information, advice, education/training & health promotion www. wirralchange.org.uk

Women’s Enterprising Breakthrough: A community organisation developed by women for women: support, empowerment, skills –building

[www.womensenterprisingbreakthrough.org](http://www.womensenterprisingbreakthrough.org)

AiW Health: Wirral’s best known charitable organisation serving those with mental health or long term health problems. Providing advocacy and support in drug and alcohol use, employment, debt, caring also provides therapeutic interventions such as Cognitive Behavioural Therapy.

[www.aiw.org.uk](http://www.aiw.org.uk)

PCAAL (Citizen’s Advice Bureau) within Group Practices – Wirral & Liverpool CCGs.

WIRED (carer’s support)Working with and for disadvantaged people and carers across the North-West.

[www.wired.me.uk](http://www.wired.me.uk)

The Livewell Programme: Supporting people to live longer, healthier lives – be more active, eat healthier, smoking cessation, relax and unwind.

wwwwirralct.nhs.uk

The **GP Forum** is a local professional peer support group now run by Kath Jones. See [www.primarycarehub.org.uk/gp-forum](http://www.primarycarehub.org.uk/gp-forum). Contact Kath for more information:

[kathjones@doctors.org.uk](mailto:kathjones@doctors.org.uk)

Professor Chris Dowrick is exploring interest in establishing a **Balint group^[[8]](#footnote-8)^** in Liverpool. The group would be facilitated by Chris and Sue Martin, meeting monthly for an hour and a half on a Saturday morning at a venue in North Liverpool. There would be a small charge. If you would be interested in finding out more, you can contact Chris directly – [cfd@liv.ac.uk](mailto:cfd@liv.ac.uk)

**APPENDIX l: FEEDBACK FROM GP EDUCATIONAL EVENT**

Full report available from the authors. Highlights from GP feedback is given below

**What did you learn from this event?**

*Different way of thinking*

- Inspired me to try a different approach
- Offered a wider perspective
- Looking at the balance of resources and demands

*Different way of doing*

- Useful and practical tips
- Good to get a chance to try out different styles of consulting
- Practical tips, new skills and ways of working
- On how to do patient centred consulting

1. Funding for JR’s time was dropped to 5%FTE to allow an extension of project funded time for LC (although JR continued to offer >10%FTE time to this project throughout). Initially LC joined the project on a part-time basis, funded by non-project funds (from JR’s Clinician Scientist Award). Once IESDF funding for LC’s time started, LC continued to work half time on the project, eventually moving to full-time. In this way we were able to secure support from LC for the project for the full 2 years. These details were explained to NIHR in the end of Year 1 report. [↑](#footnote-ref-1)
2. www.amproject.org.uk [↑](#footnote-ref-2)
3. www.economistinsights.com/healthcare/opinion/global-crisis-depression-reality-or-hype [↑](#footnote-ref-3)
4. Mark Gilman spoke eloquently about a social model of recovery. Also see Dowrick, Beyond Depression (2009); Emmy Gut & John Bowlby, Productive & unproductive depression: its functions and failures, 1989. [↑](#footnote-ref-4)
5. Instead of a more biomedical model of symptom questions and PHQ9 [↑](#footnote-ref-5)
6. Based on Reeve J. Supporting Expert Generalist Practice: the SAGE consultation model. British Journal of General Practice. *In press* [↑](#footnote-ref-6)
7. Gabbay J, le May A. Practice based evidence for healthcare: clinical mindlines. 2010 [↑](#footnote-ref-7)
8. http://balint.co.uk/about/ [↑](#footnote-ref-8)
